# Synthesis and Antileishmanial Activity of Cinnamic Acid–Amantadine Amides

**DOI:** 10.1021/acsomega.5c11247

**Published:** 2026-03-11

**Authors:** Érika Basílio Fernandes, Camila Simões de Freitas, Luciana Pereira Silva Viana, Cleiton Moreira da Silva, Natália Assis Guedes, Wanderson Romão, Valdemar Lacerda Jr, Nayara Araújo dos Santos, Fabrício Marques de Oliveira, Danilo Aniceto da Silva, Cristiane Isaac Cerceau, Mariana Belizario de Oliveira, William dos Santos Belarmino, Quesia Helena Campos Serpa, Osmair Vital de Oliveira, Adílson Vidal Costa, Eduardo Antônio Ferraz Coelho, Róbson Ricardo Teixeira

**Affiliations:** † Grupo de Síntese e Pesquisa de Compostos Bioativos (GSPCB), Departamento de Química, Universidade Federal de Viçosa, Viçosa 36570-900, MG, Brazil; ‡ Programa de Pós-Graduação em Ciências da Saúde: Infectologia e Medicina Tropical, Faculdade de Medicina, 28114 Universidade Federal de Minas Gerais, Belo Horizonte 30130-100, MG, Brazil; § Grupo de Estudos em Química Orgânica e Biológica (GEQOB), Departamento de Química, Instituto de Ciências Exatas, Universidade Federal de Minas Gerais, Belo Horizonte 31270-901, MG, Brazil; ∥ Laboratório de Petroleômica e Forense, Departamento de Química, Universidade Federal do Espírito Santo, Vitória 29075-910, ES, Brazil; ⊥ Instituto Federal de Educação, Ciência e Tecnologia de Minas Gerais, Ouro Branco 36420-000, Minas Gerais, Brazil; # Grupo de Estudo Aplicado em Produtos Naturais e Síntese Orgânica (GEAPS), Departamento de Química e Física, Universidade Federal do Espírito Santo, Alegre 29500-000, ES, Brazil; ∇ Instituto Federal de São Paulo, 15808-305 Catanduva, SP, Brazil

## Abstract

A novel series of cinnamic acid–amantadine amides was designed, synthesized, and evaluated for antileishmanial activity against *Leishmania amazonensis*, *Leishmania braziliensis*, and *Leishmania infantum*. The target compounds were obtained via amidation of cinnamic acid derivatives with amantadine, using EDC as a coupling reagent, and their structures were confirmed by IR, NMR, and HRMS analyses. Preliminary screening identified five derivatives (**13**, **16**, **18**, **19**, and **20**) with >90% inhibition of promastigote viability at micromolar concentrations. These five compounds exhibited IC_50_ values in the low micromolar range and favorable selectivity indices (SI > 9). Notably, compound **20**, a brominated derivative, displayed the highest activity (IC_50_ = 11.70–18.40 μM; SI up to 33.6), followed by compound **19** bearing a trifluoromethyl substituent (IC_50_ = 17.30–26.70 μM; SI up to 24.6). In assays with infected macrophages, compounds **16**, **19**, and **13** significantly reduced intracellular amastigote burdens (≥60% reduction), with moderate efficacy against *L. braziliensis*. Quantum chemical analyses suggest that compounds **13**, **16**, and **18** may function as reducing agents, while compounds **19** and **20** may act as oxidizing agents in redox reactions. ADMET evaluations indicate that compounds **19** and **20** possess higher hydrophobicity (reflected by the highest LogP values) and lower topological polar surface area (TPSA) than the other compounds, implying enhanced membrane permeability. These *in silico* findings suggest that increased lipophilicity and the presence of electron-withdrawing groups enhance potency, likely by improving membrane permeability and redox activity. Overall, these results highlight cinnamic acid–amantadine hybrids as promising scaffolds for developing new antileishmanial agents with improved safety profiles.

## Graphical abstract

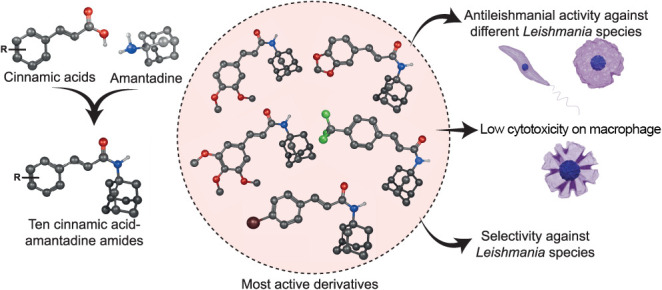

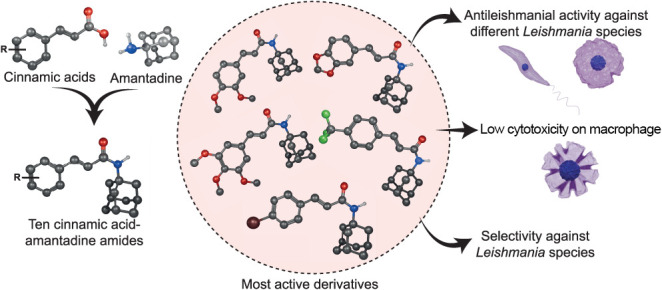

## 1 Introduction

Leishmaniasis comprises a group of diseases caused by obligate intracellular protozoa of the genus *Leishmania*.^1^ Transmission to mammalian hosts occurs through the bites of infected female sand flies of the genus *Phlebotomus* and *Lutzomyia*.^2^

The diseases are endemic in nearly 100 countries, with an estimated 0.7 to 1 million new cases annually and over 1 billion individuals at risk.^3^ Globally, they are the second most significant parasitic disease after malaria.^4^

Clinically, leishmaniasis manifests primarily as cutaneous or visceral forms. The cutaneous form is characterized by ulcers on exposed skin, frequently leading to permanent scarring and disfigurement.^5–7^ The visceral form, the most severe presentation, is potentially fatal if left untreated.^8–10^

In the absence of an effective vaccine, chemotherapy remains the primary treatment option. Pentavalent antimonials are considered first-line drugs,^11^ whereas amphotericin B (AmpB), paromomycin, miltefosine, and pentamidine (Figure 1) serve as alternative options.^12–16^ However, their clinical use is limited by severe toxicity, high cost, and the emergence of drug-resistant *Leishmania* strains.^12–16^ These drawbacks highlight the urgent need for novel, low-cost therapeutic agents with improved efficacy and safety profiles.^4^

**Figure 1:**
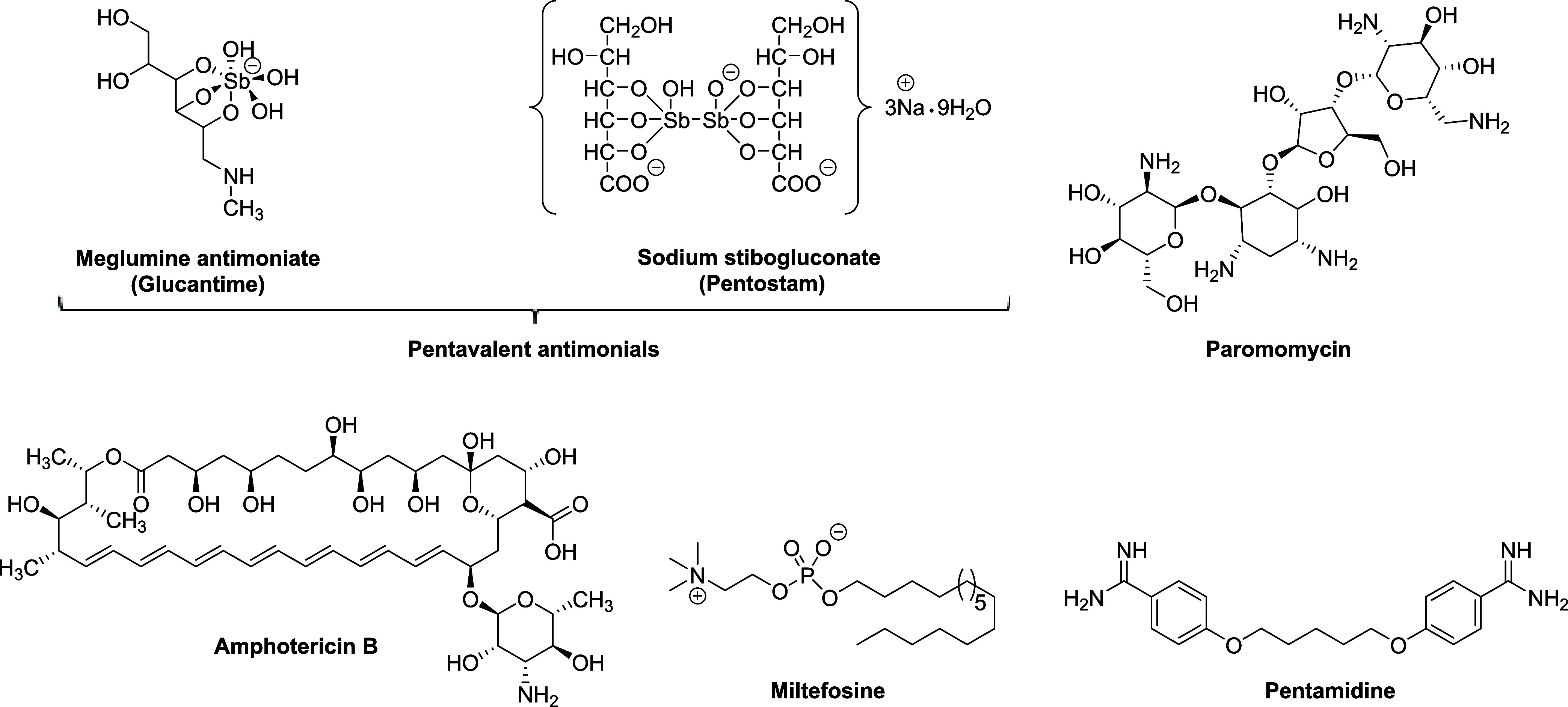
Main drugs currently used to treat leishmaniasis.

Natural products are invaluable sources of bioactive scaffolds for the discovery of antileishmanial drugs.^17–20^ Some, such as Amp B, are used directly as antileishmanials, while others provide chemical templates for the development of optimized derivatives.

Cinnamic acid (Figure 2), a naturally occurring α,β-unsaturated carboxylic acid, exhibits diverse biological activities,^21–23^ including antileishmanial effects.^24^ Our research group has previously reported cinnamic acid derivatives bearing 1,2,3-triazole and isobenzofuranone moieties (Figure 2) with significant antileishmanial activity.^25^ The derivatives 1-oxo-1,3-dihydroisobenzofuran-5-yl (*E*)-3-(3,4,5-trimethoxyphenyl)­acrylate (a cinnamate containing an isobenzofuranone moiety), (1-(3,4-difluorobenzyl)-1*H*-1,2,3-triazol-4-yl)­methyl cinnamate, and (1-(2-bromobenzyl)-1*H*-1,2,3-triazol-4-yl)­methyl cinnamate (cinnamates containing 1,2,3-triazole moieties) (Figure 2) were the most effective, showing more than 80% toxicity against *L. braziliensis* amastigotes.

**Figure 2:**
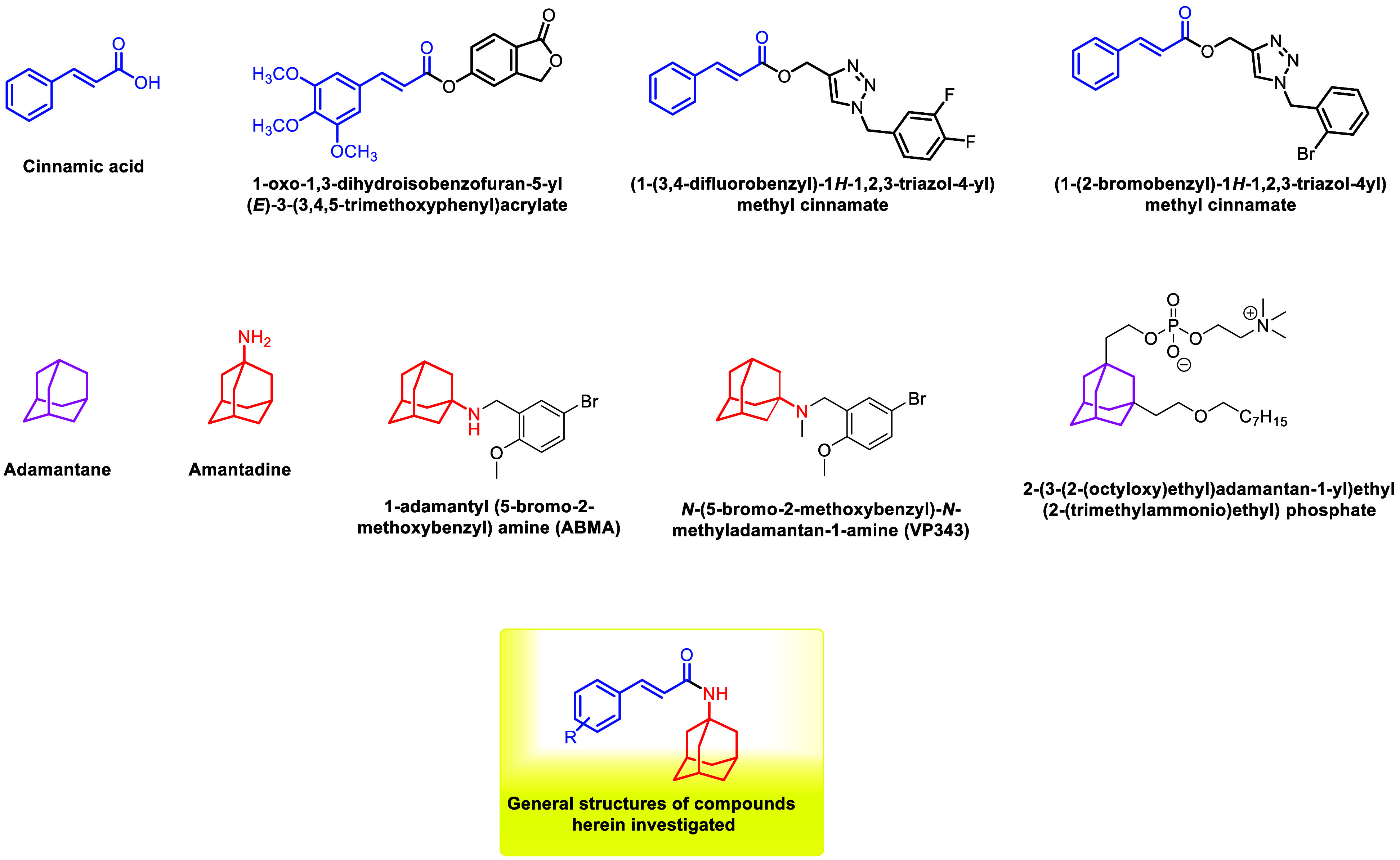
Structures of cinnamic acid (and derivatives), adamantane, amantadine (and derivatives), adamantane ether phospholipids, and general structure of compounds herein investigated.

Conversely, amantadine (Figure 2), an adamantane derivative, is well-known for its antiviral^26–30^ and antiparkinsonian^31^ properties and has been employed as a scaffold for the design of antileishmanial agents. In a 2017 study, Wu and collaborators reported that the compound 1-adamantyl (5-bromo-2-methoxybenzyl) amine (ABMA) (Figure 2) was able to inhibit the development of *L. infantum* intramacrophage amastigotes, with an EC_50_ value of 7 μM.^32^ Inspired by this result, Pomel and collaborators screened 142 compounds from a targeted library of analogues, aiming to identify molecules that act on the parasite indirectly through the host cell rather than directly on the pathogen, intending to reduce the risk of resistance development by the parasite. In *in vitro* assays, VP343 (Figure 2), a methylated analogue of ABMA, exhibited activity against the intramacrophage amastigote form of *L. infantum* with IC_50_ value of 0.32 μM, approximately 10 times more potent than miltefosine, and a selectivity index around 200, compared to miltefosine’s selectivity index of about 8. Additionally, *in vivo* assays showed a 59% reduction in liver parasite burden in mice following oral administration of VP343 at 10 mg kg^–1^ day^–1^ for 5 days.^33^

Papanastasiou and collaborators also reported the synthesis of a series of new adamantane ether phospholipids substituted with 2-[3-(2-alkyloxy-ethyl)-adamantan-1-yl]-ethoxy, whose biological activity was evaluated against the amastigote form of *L. infantum*. The results indicated that these compounds containing the adamantane core were significantly less cytotoxic than miltefosine, the positive control. The most active compound, 2-(3-(2-(octyloxy)­ethyl)­adamantan-1-yl)­ethyl (2-(trimethylammonio)­ethyl) phosphate (Figure 2), exhibited an IC_50_ value of 16.2 μM against the intracellular amastigote form of *L. infantum* field strain MK-1. Furthermore, the compound showed no cytotoxicity at a concentration of 50 μM.^34^

The adamantyl group is a rigid, bulky, cage-like hydrocarbon substituent that offers several advantages in medicinal chemistry. Its hydrophobic and lipophilic nature tends to increase membrane permeability, including penetration through the blood–brain barrier. The steric bulk and rigidity of the group contribute to restricting conformational flexibility, properly orienting substituents, and protecting nearby functional groups from metabolic enzymes or hydrolysis, which often enhances metabolic stability and prolongs the plasma half-life. In addition, its three-dimensional structure, unlike the flat phenyl ring, allows substitution with adamantane to move beyond flat-land and explore 3D chemical space, potentially improving selectivity and binding within protein targets.^35,36^ Due to these properties, this functionality is observed in several compounds exhibiting a range of activities,^36^ including those with antileishmanial effects.^32–34^

Given the proven antileishmanial potential of cinnamic acid derivatives and the antileishmanial activity of adamantyl-based compounds, we hypothesized that hybrid molecules combining these two pharmacophores could display enhanced antileishmanial activity. Herein, we describe the synthesis and biological evaluation of a series of cinnamic acid-amantadine amides (Figure 2). Several derivatives exhibited moderate activity against three *Leishmania* species, with notable selectivity toward the parasites over mammalian host cells.

## 2 Materials and Methods

### 2.1 Generalities

Reagents of analytical grade (P.A.) were used for the synthesis of the compounds. The following substances were purchased from Sigma-Aldrich (St. Louis, MO, USA) and used without prior purification: 1-adamantylamine (amantadine), (*E*)-cinnamic acid, (*E*)-4-methoxycinnamic acid, (*E*)-3,4-dimethoxycinnamic acid, 4-*N*,*N*′-dimethylaminopyridine (DMAP), 1-ethyl-3-(3-(dimethylamino)­propyl)­carbodiimide (EDC). The acids (*E*)-4-fluorocinnamic, (*E*)-4-chlorocinnamic, (*E*)-3-(benzo­[*d*]­[1,3]­dioxol-5-yl)­acrylic, (*E*)-4-nitrocinnamic, (*E*)-3,4,5-trimethoxycinnamic, (*E*)-4-trifluoromethylcinnamic, and (*E*)-4-bromocinnamic were synthesized as previously described.^37^ Solvents, including acetone, hexane, and tetrahydrofuran, were obtained from Química Moderna (Barueri, São Paulo State, Brazil). At the same time, ethyl acetate and dichloromethane were purchased from Labsynth Produtos para Laboratórios Ltd.a (Diadema, São Paulo State, Brazil). Anhydrous sodium sulfate was sourced from Dinâmica Química Contemporânea Ltda (Indaiatuba, São Paulo State, Brazil).

Thin-layer chromatography (TLC) analyses were conducted using silica gel plates coated on aluminum supports. After elution, the plates were visualized under ultraviolet light (λ = 254 nm) and developed using either a potassium permanganate solution (3 g KMnO_4_, 20 g K_2_CO_3_, 5 mL 5% NaOH w v^–1^, 300 mL distilled water) or a 10% w v^–1^ phosphomolybdic acid solution in ethanol.

Column chromatography separations were performed using silica gel 70–230 mesh (Sigma-Aldrich, St. Louis, MO, USA) as the stationary phase, with solvents for the mobile phase distilled before use.

Melting points were measured using an MQAPF-302 apparatus (Microquimica, Palhoça, Santa Catarina State, Brazil) and are reported without correction.

Infrared (IR) spectra were recorded using the attenuated total reflectance (ATR) technique on a Varian 660-IR spectrometer equipped with a GladiATR accessory (Varian, Palo Alto, CA, USA).

Nuclear magnetic resonance (NMR) spectra were obtained on a VARIAN MERCURY 300 spectrometer (Varian, Palo Alto, CA, USA) operating at 300 MHz for ^1^H and 75 MHz for ^13^C. Deuterated solvents chloroform (CDCl_3_) and dimethyl sulfoxide (DMSO-*d*_*6*_), obtained from Sigma-Aldrich, were used. Scalar coupling constants (*J*) are given in Hertz (Hz). The signal multiplicities in the ^1^H NMR spectra are denoted as follows: s (singlet), brs (broad singlet), d (doublet), t (triplet), m (multiplet), dd (doublet of doublets), and app t (apparent triplet), and hept (heptet).

The mass spectrometry (MS) analyses were performed with an Orbitrap Exploris 120 mass spectrometer (Thermo Scientific, Bremen, Germany) coupled to a Vanquish ultrahigh-performance liquid chromatography (UHPLC) system (Thermo Scientific, Bremen, Germany) for automatic direct infusion. A 5 μL aliquot of the sample was injected into a flow of 150 μL min^–1^ of methanol (0.1% formic acid, Sigma-Aldrich Chemicals, USA). High-resolution mass spectra were acquired with an *m*/*z* range of 100–1100 at a resolution of 120,000. Data-dependent acquisition was carried out using a HCD Collision Energy of 35% and a resolution of 15,000, with dynamic exclusion. The ESI parameters included: (i) ion transfer tube temperature: 320 °C; (ii) vaporizer temperature: 275 °C; (iii) sheath gas: 35 (arbitrary units); (iv) auxiliary gas: 7 (arbitrary units); and (v) source voltage: 3.5 kV in positive ionization mode (+).

### 2.2 Synthesis of Cinnamic Acid-Amantadine Amides

#### 2.2.1 Synthesis of (*E*)-*N*-(adamantan-1-yl)­cinnamamide (**11**)

In a 50 mL round-bottom flask equipped with a magnetic stirring bar, cinnamic acid (0.100 g, 0.675 mmol), 1-adamantylamine (0.102 g, 0.675 mmol), EDC (0.186 g, 1.20 mmol), DMAP (0.0120 g, 0.0980 mmol), and dichloromethane (8.00 mL) were added. The reaction mixture was stirred at room temperature, and the progress of the reaction was monitored by thin-layer chromatography (TLC). After 1 h, the reaction mixture was transferred to a separatory funnel. The organic phase was washed sequentially with cold water (10 × 15.0 mL) and 0.1 mol L^–1^ hydrochloric acid solution (1 × 15.0 mL), then dried over anhydrous sodium sulfate, filtered, and concentrated under reduced pressure. The resulting crude product was purified by column chromatography on silica gel, eluting with a hexane/ethyl acetate (2:1 v v^–1^) mixture, to afford compound **11** as a solid in 75% yield (0.143 g, 0.509 mmol).

TLC: *R*_f_ = 0.84 (hexane/ethyl acetate 2:1 v v^–1^); mp 207.0 – 208.0 °C; IR (ATR) ν/cm^–1^ 3318, 3278, 3060, 2903, 2849, 1656, 1617, 1543, 1448, 1347, 1310, 1221, 1093, 1009, 980, 869, 813, 764, 724, 652; ^1^H NMR (300 MHz, CDCl_3_) δ 2.08–1.70 (2 × brs, 15H), 5.43 (s, 1H), 6.34 (d, 1H, *J =* 15.5 Hz), 7.31–7.35 (m, 3H), 7.44–7.52 (m, 2H), 7.54 (d, 1H, *J* = 15.5 Hz); ^13^C NMR (75 MHz, CDCl_3_) δ 29.4, 36.3, 41.6, 52.2, 122.2, 127.6, 128.7, 129.3, 135.0, 140.1, 164.9; HRMS (ESI) *m*/*z* calcd. for C_19_H_24_NO [M + H]^+^: 282.18579, found: *m*/*z* 282.18390; *m*/*z* calcd. for C_19_H_23_NONa [M + Na] ^+^: 304.16773, found: *m*/*z* 304.16571.

The compounds **12–20** were prepared from adamantine and the corresponding cinnamic acid derivatives using a procedure similar to that described for the synthesis of **11**. The following data confirm the structures of the compounds.

#### 2.2.2 (*E*)-*N*-(Adamantan-1-yl)-3-(4-methoxyphenyl) Acrylamide (**12**)

TLC: *R*_f_ = 0,64 (hexane/ethyl acetate 2:1 v v^–1^); mp 161.0–163.5 °C; obtained in 78% yield. IR (ATR) ν/cm^–1^ 3314, 2909, 2853, 1655, 1606, 1536, 1510, 1454, 1356, 1305, 1286, 1250, 1218, 1170, 1103, 1029, 986, 827, 744, 707, 636; ^1^H NMR (300 MHz, CDCl_3_) δ 2.08–1.69 (2 × brs, 15H), 3.81 (s, 3H), 5.34 (s, 1H), 6.20 (d, 1H, *J* = 15.5 Hz), 6.86 (d, 2H, *J* = 8.9 Hz), 7.41 (d, 2H, *J* = 8.9 Hz), 7.49 (d, 1H, *J* = 15.5 Hz); ^13^C NMR (75 MHz, CDCl_3_) δ 29.4, 36.3, 41.7, 52.1, 55.3, 114.1, 119.8, 127.7, 129.1, 139.7, 160.6, 165.2; HRMS (ESI) *m*/*z* calcd. for C_20_H_26_NO_2_ [M + H]^+^: 312.19635, found: *m*/*z* 312.9434; *m*/*z* calcd for C_20_H_25_NO_2_Na [M + Na]^+^: 334.17830, found: *m*/*z* 334.17618.

#### 2.2.3 (*E*)-*N*-(Adamantan-1-yl)-3-(3,4-dimethoxyphenyl) Acrylamide (**13**)

TLC: *R*_f_ = 0.62 (dichloromethane/hexane/ethyl acetate 1:1:1 v v^–1^); prepared in 61% yield. IR (ATR) ν/cm^–1^ 3314, 2911, 2851, 1655, 1605, 1510, 1453, 1420, 1358, 1304, 1254, 1214, 1137, 1102, 1026, 986, 827, 810, 764, 741; ^1^H NMR (300 MHz, CDCl_3_) δ 2.07–1.68 (2 × brs, 15H), 3.88 (2 × *s*, 6H), 5.35 (s, 1H), 6.21 (d, 1H, *J* = 15.5 Hz), 6.99 (d, 1H, *J* = 8.7 Hz), 6.99 (d, 1H, *J* = 2.0 Hz), 7.04 (dd, 1H, *J* = 8.7 Hz, *J* = 2.0 Hz), 7.47 (d, 1H, *J* = 15.5 Hz); ^13^C NMR (75 MHz, CDCl_3_) δ 29.4, 36.3, 41.7, 52.1, 55.8, 109.5, 111.0, 120.1, 121.8, 128.0, 139.9, 149.0, 150.0, 165.1; HRMS (ESI) *m*/*z* calcd. for C_21_H_28_NO_3_ [M + H]^+^: 342.20692, found: 342.20483; *m*/*z* calcd for C_21_H_27_NO_3_ [M + Na]^+^: 364.18886, found: 364.18665.

#### 2.2.4 (*E*)-*N*-(Adamantan-1-yl)-3-(4-fluorophenyl) Acrylamide (**14**)

TLC: *R*_f_ = 0.77 (hexane/ethyl acetate 2:1 v v^–1^); mp 225.8–226.7 °C; obtained in 57% yield. IR (ATR) ν/cm^–1^ 3293, 3072, 2907, 2858, 1655, 1613, 1546, 1506, 1453, 1359, 1348, 1309, 1222, 1156, 1102, 1009, 991, 829, 793, 741, 701, 656; ^1^H NMR (300 MHz, CDCl_3_) δ 2.07–1.70 (2 × brs, 15H), 5.40 (s, 1H), 6.26 (d, 1H, *J* = 15.5 Hz), 7.02 (app t, 2H, *J* = 8.7 Hz), 7.47–7.42 (m, 2H), 7.50 (d, 1H, *J* = 15.5 Hz); ^13^C NMR (75 MHz, CDCl_3_) δ 29.4, 36.3, 41.6, 52.2, 115.8 (d, *J*_*C–F*_ = 21.4 Hz), 121.9, 129.4 (d, *J*_*C–F*_ = 8.3 Hz), 131.2 (d, *J*_*C–F*_ = 3.1 Hz), 138.9, 163.3 (d, *J*_*C–F*_ = 248.4 Hz), 164.8; HRMS (ESI) *m*/*z* calcd. for C_19_H_23_FNO [M + H]^+^: 300.17637, found: 300.17441; *m*/*z* calcd for C_19_H_22_FNONa [M + Na]^+^: 322.15831, found: 322.15625.

#### 2.2.5 (*E*)-*N*-(Adamantan-1-yl)-3-(4-chlorophenyl) Acrylamide (**15**)

TLC: *R*_f_ = 0.73 (hexane/ethyl acetate 3:1 v v^–1^); mp 228.3–231.4 °C; obtained in 58% yield. IR (ATR) ν/cm^–1^ 3310, 2903, 2858, 1657, 1618, 1540, 1490, 1452, 1406, 1357, 1347, 1338, 1309, 1217, 1089, 1008, 988, 873, 817, 758, 632; ^1^H NMR (300 MHz, CDCl_3_) δ 2.07–1.69 (2 × brs, 15H), 5.38 (s, 1H), 6.30 (d, 1H, *J* = 15.5 Hz), 7.30 (d, 2H, *J* = 8.5 Hz), 7.39 (d, 2H, *J* = 8.5 Hz), 7.48 (d, 1H, *J* = 15.5 Hz); ^13^C NMR (75 MHz, CDCl_3_) δ 29.4, 36.3, 41.6, 52.2, 122.7, 128.8, 128.9, 133.5, 135.2, 138.8, 164.5; HRMS (ESI) *m*/*z* calcd. for C_19_H_23_ClNO [M + H]^+^: 316.14682, found: 316.14487; *m*/*z* calcd for C_19_H_22_ClNONa [M + Na]^+^: 338.12876, found: 338.12680.

#### 2.2.6 (*E*)-*N*-(Adamantan-1-yl)-3-(benzo­[*d*]­[1,3] dioxol-5-yl) Acrylamide (**16**)

TLC: *R*_f_ = 0.51 (dichloromethane/hexane/ethyl acetate 1:1:1 v v^–1^); mp 163.5–165.7 °C; obtained in 61% yield. IR (ATR) ν/cm^–1^ 3252, 3072, 2903, 2848, 1653, 1615, 1555, 1488, 1445, 1359, 1332, 1246, 1188, 1100, 1040, 1009, 933, 851, 812, 744, 683; ^1^H NMR (300 MHz, CDCl_3_) δ 2.07–1.70 (2 × brs, 15H), 5.33 (s, 1H), 5.97 (s, 2H), 6.15 (d, 1H, *J* = 15.4 Hz), 6.77 (d, 1H, *J* = 7.9 Hz), 6.93–6.97 (m, 2H), 7.44 (d, 1H, *J* = 15.4 Hz); ^13^C NMR (75 MHz, CDCl_3_) δ 29.4, 36.3, 41.7, 52.1, 101.3, 106.2, 108.4, 120.2, 123.6, 129.4, 139.8, 148.1, 148.8, 165.0; HRMS (ESI) *m*/*z* calcd. for C_20_H_24_NO_3_ [M + H]^+^: 326.17562, found: 326.17352; *m*/*z* calcd for C_20_H_23_NO_3_Na [M + Na]^+^: 348.15756, found: 348.15540.

#### 2.2.7 (*E*)-*N*-(Adamantan-1-yl)-3-(4-nitrophenyl) Acrylamide (**17**)

TLC: *R*_f_ = 0.63 (dichloromethane/hexane/ethyl acetate 1:1:1 v v^–1^); mp 187.0–188.9 °C; obtained in 61% yield. IR (ATR) ν/cm^–1^ 3601, 3401, 3279, 3079, 2914, 2850, 1657, 1616, 1552, 1514, 1344, 1225, 1108, 1005, 979, 869, 848, 834, 740, 676; ^1^H NMR (300 MHz, CDCl_3_) δ 2.08–1.70 (2 × brs, 15H), 5.49 (s, 1H), 6.47 (d, 1H, *J* = 15.5 Hz), 7.54–7.62 (m, 3H), 8.20 (d, 2H, *J* = 8.6 Hz); ^13^C NMR (75 MHz, CDCl_3_) δ 29.4, 36.2, 41.6, 52.5, 124.0, 126.4, 128.2, 137.5, 141.3, 147.9, 163.6; HRMS (ESI) *m*/*z* calcd. for C_19_H_23_N_2_O_3_ [M + H]^+^: 327.17087, found: 327.16882; *m*/*z* calcd for C_19_H_22_N_2_O_3_Na [M + Na]^+^: 349.15281, found: 349.15079.

#### 2.2.8 (*E*)-*N*-(Adamantan-1-yl)-3-(3,4,5-trimethoxyphenyl) Acrylamide (**18**)

TLC: *R*_f_ = 0.31 (hexane/ethyl acetate 2:1 v v^–1^); obtained in 49% yield. IR (ATR) ν/cm^–1^ 3601, 3277, 3072, 2911, 2849, 1657, 1616, 1505, 1453, 1417, 1325, 1279, 1239, 1125, 1005, 979, 831, 739, 675; ^1^H NMR (300 MHz, DMSO-*d*_6_) δ 1.63–2.50 (2 × brs, 9H), 3.66 (s, 3H), 3.79 (s, 6H), 6.59 (d, 1H, *J* = 15.6 Hz), 6.83 (s, 2H), 7.24 (d, 1H, *J* = 15.6 Hz), 7.48 (s, 1H); ^13^C NMR (75 MHz, DMSO-*d*_6_) δ 29.2, 36.5, 41.4, 51.2, 56.2, 60.5, 105.1, 123.6, 131.2, 138.2, 138.8, 153.4, 164.5; HRMS (ESI) *m*/*z* calcd. for C_22_H30NO_4_ [M + H]^+^: 372.21748, found: *m*/*z* 372.21509; *m*/*z* calcd for C_22_H_29_NO_4_Na [M + Na]^+^: 394.19943, found: 394.19681.

#### 2.2.9 (*E*)-*N*-(Adamantan-1-yl)-3-(4-(trifluoromethyl) phenyl) Acrylamide (**19**)

TLC: *R*_f_ = 0.63 (hexane/ethyl acetate 2:1 v v^–1^); mp 132.5–134.4 °C; obtained in 57% yield. IR (ATR) ν/cm^–1^ 3271, 3079, 2903, 2851, 1659, 1617, 1551, 1452, 1360, 1319, 1226, 1168, 1127, 1065, 989, 833, 747, 716, 641; ^1^H NMR (300 MHz, DMSO-*d*_6_) δ 1.63−2.50 (2 x brs, 9H), , 6.79 (d, 1H, *J* = 15.7 Hz), 7.38 (d, 1H, *J* = 15.7 Hz), 7.66 (s, 1H), 7.70−7.76 (m, 4H); ^13^C NMR (75 MHz, DMSO-*d*_6_) δ 29.2, 36.4, 41.3, 51.4, 124.5 (q, 270.3 Hz), 126.2 (q, *J* = 3.8 Hz), 127.1, 128.4, 129.4 (q, *J* = 31.7 Hz), 136.5, 139.7, 163.9; HRMS (ESI) *m*/*z* calcd. for C_20_H_23_F_3_NO [M + H]^+^: 350.17317, found: 350.17111; *m*/*z* calcd for C_20_H_22_F_3_NONa [M + Na]^+^: 372.15512, found: 372.15280.

#### 2.2.10 (*E*)-*N*-(Adamantan-1-yl)-3-(4-bromophenyl) Acrylamide (**20**)

TLC: *R*_f_ = 0.79 (dichloromethane/hexane/ethyl acetate 1:1:1 v v^–1^); mp 216.0–218.4 °C; obtained in 69% yield. IR (ATR) ν/cm^–1^ 3314, 3058, 2903, 2848, 1658, 1616, 1536, 1486, 1357, 1347, 1334, 1307, 1218, 1070, 1007, 986, 872, 814, 756, 628; ^1^H NMR (300 MHz, DMSO-*d*_6_) δ 1.62–2.50 (2 × brs, 15H), 6.67 (d, 1H, *J* = 15.7 Hz), 7.27 (d, 1H, *J* = 15.7 Hz), 7.45 (d, 2H, *J* = 8.6 Hz), 7.56–7.61 (m, 3H); ^13^C NMR (75 MHz, DMSO-*d*_6_) δ 29.2, 36.4, 41.4, 51.3, 122.7, 125.1, 129.7, 132.3, 134.9, 136.9, 164.2; HRMS (ESI) *m*/*z* calcd. for C_19_H_23_BrNO [M + H]^+^: 360.09630, found: 360.09436; *m*/*z* calculated for C_19_H_22_BrNONa [M + Na] ^+^: 382.07825, found: 382.07593.

Spectroscopic and spectrometric data used to characterize the compounds are available in the Supporting Information.

### 2.3 Biological Assays

#### 2.3.1 Ethical and Parasite Culture

Experiments were performed in accordance with the International Ethical Guidelines for the care and use of laboratory animals and were approved by the Ethical Committee for Animal Research of the Federal University of Minas Gerais (UFMG), under protocol number 056/2022. Female BALB/c mice, 6 weeks of age, were obtained from the UFMG Bioterium Center and housed under specific pathogen-free (SPF) conditions with controlled temperature, humidity, and a 12 h light/dark cycle. The *Leishmania* strains used in this study included: *L. amazonensis* (IFLA/BR/1967/PH-8), *L. braziliensis* (MHOM/BR/1975/M2903), and *L. infantum* (MHOM/BR/1970/BH46). Parasites were cultured at 24 °C in Schneider’s Drosophila insect medium (Sigma-Aldrich, St. Louis, MO, USA), supplemented with 20% (v v^–1^) heat-inactivated fetal bovine serum (FBS) (Gibco, Waltham, Massachusetts, USA), 20 mmol L^–1^ l-glutamine, penicillin (200 U mL^–1^), streptomycin (100 μg mL^–1^), and gentamicin (50 μg mL^–1^) (Sigma-Aldrich, St. Louis, MO, USA). The culture medium was adjusted to a physiological pH of 7.4, and parasites were maintained in logarithmic growth phase following the protocol previously described.^38^

#### 2.3.2 Antileishmanial Screening of Synthesized Compounds

The synthesized compounds **11**–**20** were initially tested in a single-point screening assay against *Leishmania infantum*, *L. amazonensis*, and *L. braziliensis* promastigotes. Stationary promastigotes (1 × 10^6^ cells/well) were incubated in 96-well flat-bottom plates (Nunc, Thermo Fisher Scientific, USA) in Schneider’s insect medium supplemented with 10% heat-inactivated FBS and 1% penicillin-streptomycin-gentamicin. Each compound was tested at a fixed concentration of 100 μg mL^–1^, with DMSO at ≤ 0.5% (v v^–1^) as the vehicle control. using both positive and negative controls to ensure assay validity. AmpB (2.5 μg mL^–1^; Sigma-Aldrich, USA) was used as a control. After 48 h at 25 °C, parasite viability was assessed by the 3-(4,5-dimethylthiazol-2-yl)-2,5-diphenyltetrazolium bromide (MTT) (St. Louis, MO, USA) method, measuring absorbance at 570 nm using a microplate reader (SpectraMax Plus, Molecular Devices, San Jose, CA, USA). The growth inhibition percentage was calculated relative to the untreated controls, and compounds showing >50% growth inhibition were selected for further evaluation.

#### 2.3.3 Viability Curves of the Parasites Using the Selected Compounds

Compounds **13**, **16**, **18**, **19**, and **20** were identified as the most active in the initial trial, and they were evaluated for their 50% inhibitory concentration (IC_50_) against *L. infantum*, *L. amazonensis*, and *L. braziliensis* stationary promastigotes. Parasites (1 × 10^6^ cells/well) were incubated in 96-well plates with serial dilutions of each compound (0 to 200 μg mL^–1^) for 48 h at 25 °C. AmpB (0 to 10 μg mL^–1^) served as the reference drug. Parasite viability was determined by the MTT method as described above. IC_50_ values were obtained by nonlinear regression analysis (sigmoidal dose–response curve with variable slope) using GraphPad Prism version 10.0.2 (GraphPad Software, San Diego, CA, USA).

#### 2.3.4 Cytotoxicity Assay and Determination of the Selectivity index

Murine peritoneal macrophages were obtained from female BALB/c mice (8 weeks old) via peritoneal lavage with 5 mL cold phosphate-buffered saline (PBS). Cells were centrifuged (1000*g*, 10 min), resuspended in RPMI 1640 medium (Gibco, USA) supplemented with 10% heat-inactivated FBS and 1% penicillin-streptomycin-gentamicin, and seeded at a density of 5 × 10^5^ cells/well in 96-well plates. After 2 h at 37 °C in a humidified atmosphere with 5% CO_2_, nonadherent cells were removed, and adherent macrophages were incubated with serial dilutions of each test compound (**13**, **16**, **18**, **19**, and **20**) (0 to 200 μg mL^–1^) for 48 h under the same conditions. AmpB (0 to 10 μg mL^–1^) served as the reference drug. Cell viability was assessed by the MTT method, and CC_50_ values were calculated as described for IC_50_ determination. The Selectivity Index (SI) was calculated as (SI = CC_50_/IC_50_).

#### 2.3.5 Treatment of Infected Macrophages and Inhibition of Infection

For infection assays, murine peritoneal macrophages (5 × 10^5^ cells/well) were seeded on sterile glass coverslips in 24-well plates and incubated for 24 h in RPMI 1640 medium supplemented with 20% FBS and 20 mmol L^–1^ l-glutamine (pH 7.4) at 37 °C in 5% CO_2_. Stationary-phase promastigotes were added at a parasite-to-cell ratio of 10:1 and incubated for 24 h. Noninternalized parasites were removed by washing three times with warm medium. Infected macrophages were treated with the selected compounds (**13**, **16**, **18**, **19**, and **20**) at the concentrations 10, 20, and 40 μg mL^–1^ (determined based on IC_50_ values) for 48 h. AmpB (1.0 μg mL^–1^) was included as the reference drug. Cells were then fixed with 4% paraformaldehyde (w v^–1^) for 20 min, washed with PBS, and stained using the commercial Panótico Rápido stain (Catalog #620529, Laborclin, Pinhais, Paraná, Brazil), according to the manufacturer’s instructions. Slides were examined under a light microscope (×1000 magnification), counting at least 200 macrophages per coverslip in triplicate. The following parameters were determined: percentage of infected macrophages, mean number of amastigotes per infected macrophage, and reduction of infection rate (%) compared to untreated controls.

#### 2.3.6 Statistical Analysis

The half-maximal inhibitory concentration (IC_50_), cytotoxic concentration (CC_50_), and hemolytic concentration (RBC_50_) values were determined by nonlinear sigmoidal regression of dose–response curves generated using GraphPad Prism software (version 6.0, GraphPad Software Inc., San Diego, CA, USA). Results were expressed as mean ± standard deviation (SD). Statistical comparisons among groups were performed using one-way analysis of variance (ANOVA) followed by Bonferroni’s post hoc test. At least two independent experiments were carried out, yielding reproducible results, and the data presented are representative of one experiment. Differences were considered statistically significant at *P* < 0.05.

### 2.4 Quantum Chemical Calculations

The structures of compounds **13**, **16**, **18**, **19**, and **20** were drawn and preoptimized using Avogadro software (Avogadro: an open-source molecular builder and visualization tool, version 1.93.0, http://avogadro.cc/). Subsequently, their structures were optimized using density functional theory (DFT) with the B3LYP hybrid functional in the gas phase.^39,40^ The minimum energy structures were confirmed by the absence of imaginary frequencies in the vibrational frequency analysis. DFT calculations were performed using the ORCA package, version 5.0.1.^41^

## 3 Results and Discussion

### 3.1 Preparation of Amides Derived from Cinnamic Acids and Amantadine

The synthesis of cinnamic acid–amantadine amides is outlined in Scheme 1. Thus, the amidation reactions of cinnamic acids **1**–**10** with amantadine, using EDC as the coupling reagent,^42^ afforded the target amantadine derivative amides in yields ranging from 49 to 78%. The compounds (*E*)-4-fluorocinnamic acid (**4**), (*E*)-4-chlorocinnamic acid (**5**), (*E*)-3-(benzo­[*d*]­[1,3]­dioxol-5-yl)­acrylic acid (**6**), (*E*)-4-nitrocinnamic acid (**7**), (*E*)-3,4,5-trimethoxycinnamic acid (**8**), (*E*)-4-trifluoromethylcinnamic acid (**9**), and (*E*)-4-bromocinnamic acid (**10**) were synthesized via the Knoevenagel condensation of the corresponding aldehydes with malonic acid.^37^

**Scheme 1:**
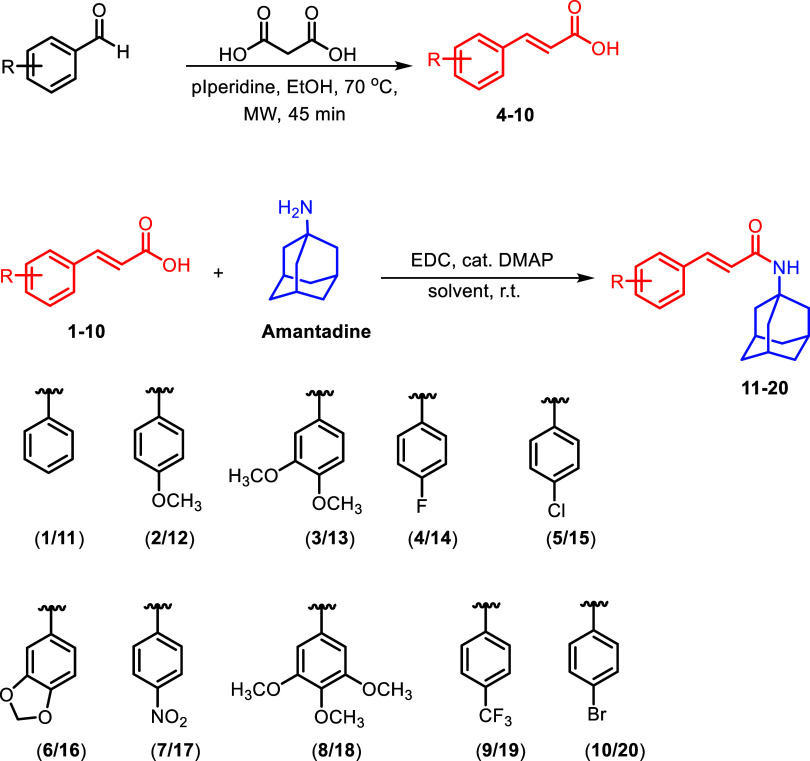
Steps Involved in the Preparation of Amides **11**–**20**

All compounds were characterized by infrared (IR) spectroscopy, nuclear magnetic resonance (NMR) spectroscopy, and mass spectrometry (MS). In the IR spectra, the characteristic carbonyl (C=O) stretching band appeared in the range of 1653–1659 cm^–1^. In the ^13^C NMR spectra, the carbonyl carbon resonated at 164–165 ppm, while signals corresponding to the adamantyl carbons were observed between 25–45 ppm. In the ^1^H NMR spectra, the adamantyl hydrogens appeared at 2–4 ppm, whereas the amide NH hydrogen resonated at approximately 5.30–5.50 ppm in CDCl_3_ and at 7.45–7.70 ppm in DMSO-*d*_6_. The *trans* stereochemistry of the aliphatic double bond was confirmed by the coupling constant (∼16 Hz) between the hydrogens attached to carbons of this bond. The molecular formulas of all synthesized compounds were confirmed by LC-MS analysis.

Two points deserve comment. First, the present work was designed as an initial proof-of-concept investigation. Thus, established and reliable synthetic methodologies, such as EDC-mediated amidation, were intentionally used to ensure reproducibility, efficiency, and chemical robustness. These approaches are standard in both academic and industrial medicinal chemistry and were selected to support the biological investigation rather than to introduce synthetic novelty *per se*. Second, the selection of substituents on the cinnamate aromatic ring was guided by previous studies from our research group on cinnamic acid derivatives. In these earlier investigations, compounds bearing halogen, trifluoromethyl, methoxy, and nitro substituents on the aromatic ring exhibited significant leishmanicidal activity.^25^ Based on these structure–activity relationship (SAR) findings, the same substituent patterns were deliberately selected in the present study, which also focuses on cinnamic acid derivatives, to explore and expand upon the previously observed biological profile.

Once synthesized, the cinnamic acid amantadine amides were evaluated against *Leishmania amazonensis*, *L. braziliensis* (etiologic agents of tegumentary leishmaniasis), and *L. infantum* (etiologic agent of visceral leishmaniasis).

### 3.2 Biological Results

The initial screening of the synthesized compounds against the promastigote stage of the parasites revealed that several compounds exhibited notable inhibitory activity at the tested fixed concentration (Table 1). Among them, compounds **13**, **16**, **18**, **19**, and **20** demonstrated strong leishmanicidal activity, with inhibition rates consistently above 90% for all tested species, comparable to the reference drug AmpB. In contrast, other compounds showed only modest or negligible inhibition, such as compounds **11**, **14**, **15**, and **17**, which did not reach the 50% inhibition threshold. Based on these results, the five most active compounds (**13**, **16**, **18**, **19**, and **20**) were selected for further biological assays.

**Table 1:** Single-Point Screening of the Synthesized Compounds Against Distinct *Leishmania* Promastigotes^a^

**compounds**	**concentration (μM)** ^b^	*L. amazonensis*	*L. braziliensis*	*L. infantum*
**11**	355.4	22%	31%	6%
**12**	321.0	61%	51%	24%
**13**	292.8	99%	99%	99%
**14**	334.0	0%	9%	0%
**15**	316.6	0%	15%	0%
**16**	307.3	93%	100%	99%
**17**	306.3	43%	39%	10%
**18**	269.2	95%	98%	99%
**19**	286.2	100%	99%	99%
**20**	277.5	93%	89%	93%
amphotericin B	2.71	100%	100%	100%

a The viability of *L. infantum*, *L. amazonensis*, and *L. braziliensis* promastigotes was evaluated after incubation with synthesized compounds at a fixed concentration (100 μg mL^–1^). Parasite viability was determined by the MTT method, and results were expressed in terms of percentage inhibition relative to untreated controls. Amphotericin B (2.5 μg mL^–1^) was used as the reference drug. Compounds showing >50% inhibition for the three different species were selected for further evaluation.

b Concentration, in μM, of each compound corresponding to 100 μg mL^–1^.

The selected compounds were evaluated for their half-maximal inhibitory concentration (IC_50_) against promastigote forms of the three *Leishmania* species and for their cytotoxicity (CC_50_) in murine peritoneal macrophages to determine their selectivity index (SI) (Table 2).

**Table 2:** *In Vitro* Antileishmanial Activity, Cytotoxicity, and Selectivity Index of the Selected Compounds^a^

		*L. amazonensis*		*L. braziliensis*		*L. infantum*	
**compounds**	**CC**_**50**_ **(μM)**	**IC**_**50**_ **(μM)**	**SI**	**IC**_**50**_ **(μM)**	**SI**	**IC**_**50**_ **(μM)**	**SI**
**13**	455.3 ± 35.00	92.8 ± 5.0	4.90	42.0 ± 2.2	10.84	82.8 ± 3.1	5.50
**16**	409.4 ± 33.6	41.4 ± 1.8	9.90	15.1 ± 0.8	27.11	39.3 ± 1.9	10.41
**18**	461.5 ± 43.4	63.7 ± 4.1	7.24	38.1 ± 2.5	12.11	35.2 ± 1.9	13.12
**19**	426.6 ± 32.4	33.8 ± 1.9	12.62	17.3 ± 1.1	24.61	69.0 ± 1.0	6.18
**20**	394.1 ± 28.0	16.4 ± 0.6	24.02	11.7 ± 0.6	33.55	43.6 ± 1.2	9.05
amphotericin B	0.89 ± 0.09	0.23 ± 0.01	3.15	0.23 ± 0.02	3.90	0.15 ± 0.03	5.86

a Half-maximal inhibitory concentration (IC_50_) against *L. infantum*, *L. amazonensis*, and *L. braziliensis* promastigote, as well as half-maximal cytotoxic concentration (CC_50_) against murine macrophages, were evaluated. The selectivity index (SI) was also calculated as the ratio of CC_50_ to IC_50_. Amphotericin B (AmpB) was used as the reference drug. Values represent the mean ± standard deviation (SD) from at least two independent experiments performed in duplicate.

All tested compounds exhibited moderate activity against *Leishmania* species, with IC_50_ values in the low micromolar range. Among them, compound **20** showed the highest potency, with IC_50_ values of 16.4 ± 0.6 μM for *L. amazonensis*, 11.7 ± 0.6 μM for *L. braziliensis*, and 43.6 ± 1.2 μM for *L. infantum*. This compound also displayed the most favorable selectivity indices, reaching 24.02, 33.55, and 9.05, respectively, indicating a promising safety and efficacy profile.

Compound **16** and compound **19** also exhibited strong activity, particularly against *L. braziliensis*, with IC_50_ values of 15.1 ± 0.8 μM (SI = 27.11) and 17.3 ± 1.1 μM (SI = 24.61), respectively. On the other hand, compound **13** showed moderate potency, with SI values ranging from 4.90 to 10.84, suggesting a narrower therapeutic window. When compared to AmpB, which showed nanomolar IC_50_ values but also exhibited high cytotoxicity (CC_50_ = 0.89 ± 0.09 μM), the synthesized compounds demonstrated a superior selectivity profile, highlighting their potential as safer alternatives to current treatments.

The treatment of *L. amazonensis*-infected macrophages with the selected compounds demonstrated a dose-dependent reduction in infection parameters (Table 3). Compound **19** showed the most pronounced effect, achieving up to 63.6% reduction in amastigotes at 114.5 μM, while compound **18** also exhibited strong activity, with 55.7% reduction at the same concentration. Compound **20** was the least effective, with a maximum decrease of 43.8%. Although all compounds reduced infection to some extent, their activity remained lower than AmpB, which led to a 72.5% reduction in infected macrophages and a 55.4% decrease in amastigotes per macrophage at 1.08 μM.

**Table 3:** Effect of Selected Compounds on *L. amazonensis*-Infected Macrophages^a^

**compounds**	**concentration (** ***μ*** **M)**	**reduction of infected macrophages (%)**	**number of amastigotes per infected macrophage**	**reduction of amastigotes (%)**
**13**	29.3	1.94	9.5 ± 0,05	41.03
	58.6	9.43	8.9 ± 0.50	44.84
	117.2	16.91	7.2 ± 0.37	55.26
**16**	30.7	2.92	10.5 ± 0.17	35.09
	61.5	8.40	9.9 ± 0.08	38.48
	123.0	14.43	8.8 ± 0.24	45.74
**18**	26.9	6.68	9.8 ± 0.12	39.18
	53.8	5.47	8.1 ± 0.10	50.09
	107.6	11.85	7.2 ± 0.07	55.68
**19**	28.6	1.99	10.2 ± 0.06	37.08
	57.3	8.98	8.1 ± 0.28	49.87
	114.5	16.13	5.9 ± 0.08	63.58
**20**	27.8	2.96	11.2 ± 0.05	30.91
	55.5	9.83	10.1 ± 0.19	37.47
	111.0	15.60	9.1 ± 0.40	43.75
**AmpB**	1.08	72.48	7.2 ± 1.00	55.35

a The infection reduction was evaluated in *L. amazonensis*-infected macrophages after treatment with the selected compounds. Parameters evaluated included the percentage of infected macrophages, the mean number of amastigotes per infected macrophage, and the percentage reduction of amastigotes compared to untreated controls. Amphotericin B (AmpB) was used as the reference drug. Percentage values represent derived parameters calculated from absolute counts of infected macrophages and intracellular amastigotes. As these values are not independent experimental measurements, variability metrics (mean ± range) are not reported. Primary quantitative data were obtained from two independent experiments performed in triplicate, and results are expressed as mean ± standard deviation (SD). Results are expressed as mean ± standard deviation (SD) of at least two independent experiments performed in triplicate.

Evaluations were also performed in *L. braziliensis*-infected macrophages. Results showed that compounds **16**, **19**, and **20** exhibited the highest inhibitory potential (Table 4), with reductions of 72.6, 71.9, and 72.3% in infected macrophages, respectively, at 40 μg mL^–1^, corresponding to 123.0, 114.5, and 111.0 μM, respectively. Compound **13** also showed significant activity, reducing infection by 62.7%. In contrast, compound **18** was less effective, with a maximum reduction of 59.9%. AmpB, used as a control, induced a 42.6% reduction in infected macrophages and over 53% reduction in amastigotes, highlighting that some of the tested compounds demonstrated comparable or superior effects against intracellular *L. braziliensis*. For the treatment of *L. infantum*-infected macrophages, compound **13** exhibited the strongest activity (Table 5), a 64.9% reduction was found when 117.2 μM was used, followed closely by compounds **16** (62.7%) and **19** (61.0%). Compounds **18** and **20** were less effective, showing reductions below 56% at the highest concentration tested. Despite the overall dose-dependent inhibition, AmpB remained more potent, achieving a 69.9% reduction in infected macrophages at just 1.08 μM. It is also important to note that although the compounds significantly reduce parasite burden, they were tested at higher concentrations than amphotericin B.

**Table 4:** Effect of Selected Compounds on *L. braziliensis*-Infected Macrophages^a^

**compounds**	**concentration (μM)**	**reduction of infected macrophages (%)**	**number of amastigotes per infected macrophage**	**reduction of amastigotes (%)**
**13**	29.3	45.80	7.2 ± 0.24	31.31
	58.6	38.81	5.0 ± 0.11	52.54
	117.2	62.66	4.7 ± 0.17	55.18
**16**	30.7	36.54	8.5 ± 0.01	18.88
	61.5	53.13	7.5 ± 0.13	28.79
	123.0	72.64	6.4 ± 0.17	39.31
**18**	26.9	20.41	10.2 ± 0.06	2.94
	53.8	46.85	9.5 ± 0.05	9.78
	107.6	59.97	9.2 ± 0.17	12.81
**19**	28.6	26.43	9.5 ± 0.15	9.81
	57.3	62.55	8.7 ± 0.20	14.37
	114.5	71.90	8.3 ± 0.46	21.38
**20**	27.8	27.64	9.1 ± 0.20	13.91
	55.5	51.92	8.5 ± 0.37	19.50
	111.0	72.29	7.7 ± 0.55	26.74
**AmpB**	1.08	42.64	4.9 ± 0.16	53.85

a The reduction of infection in murine peritoneal macrophages infected with *L. braziliensis* was evaluated after treatment with the selected compounds. Parameters evaluated include percentage of infected macrophages, mean number of amastigotes per infected macrophage, and percentage reduction of amastigotes compared to untreated controls. Amphotericin B (AmpB) was used as the reference drug. Percentage values represent derived parameters calculated from absolute counts of infected macrophages and intracellular amastigotes. As these values are not independent experimental measurements, variability metrics (mean ± range) are not reported. Primary quantitative data were obtained from two independent experiments performed in triplicate, and results are expressed as mean ± standard deviation (SD). Results are expressed as mean ± standard deviation (SD) of at least two independent experiments performed in triplicate.

**Table 5:** Effect of Selected Compounds on *L. infantum*-Infected Macrophages^a^

**compounds**	**concentration (μM)**	**reduction of infected macrophages (%)**	**number of amastigotes per infected macrophage**	**reduction of amastigotes (%)**
**13**	29.3	28.66	8.0 ± 0.06	16.89
	58.6	35.84	6.2 ± 0.24	35.13
	117.2	64.92	3.9 ± 0.24	59.33
**16**	30.7	18.68	7.5 ± 0.08	22.07
	61.5	31.17	5.9 ± 0.34	38.05
	123.0	62.70	5.0 ± 0.11	47.94
**18**	26.9	26.17	8.0 ± 0.74	16.95
	53.8	35.84	7.4 ± 0.03	22.47
	107.6	55.04	7.1 ± 0.36	25.65
**19**	28.6	28.66	9.1 ± 0.04	5.08
	57.3	46.16	6.7 ± 0.25	30.31
	114.5	60.97	5.5 ± 0.34	42.79
**20**	27.8	15.88	9.2 ± 0.27	4.29
	55.5	42.17	7.6 ± 0.07	20.29
	111.0	55.59	5.6 ± 0.16	41.98
**AmpB**	1.08	69.85	9.1 ± 0.12	5.22

a The reduction of infection in murine peritoneal macrophages infected with *L. infantum* was evaluated after treatment with the selected compounds. Parameters evaluated include percentage of infected macrophages, mean number of amastigotes per infected macrophage, and percentage reduction of amastigotes compared to untreated controls. Amphotericin B (AmpB) was used as the reference drug. Results are expressed as mean ± standard deviation (SD) of at least two independent experiments performed in triplicate. Percentage values represent derived parameters calculated from absolute counts of infected macrophages and intracellular amastigotes. As these values are not independent experimental measurements, variability metrics (mean ± range) are not reported. Primary quantitative data were obtained from two independent experiments performed in triplicate, and results are expressed as mean ± standard deviation (SD).

It is important to note that the percentage of infected macrophages and the number of amastigotes per infected cell are distinct biological parameters. A reduction in the percentage of infected cells does not necessarily correlate with a proportional decrease in intracellular parasite burden, as residual infected macrophages may still harbor multiple amastigotes. This distinction explains the observed differences between infection rate reduction and amastigote load reduction, particularly for amphotericin B in L. infantum–infected macrophages.

As previously stated, the present study evaluated a panel of newly synthesized compounds against *L. amazonensis*, *L. braziliensis*, and *L. infantum*, targeting both the promastigote and intracellular amastigote stages. A consistent pattern of biological activity was observed, with compounds **16**, **19**, and **13** emerging as the most promising candidates due to their potent leishmanicidal effects. These molecules reduced both the percentage of infected macrophages and the intracellular parasite burden to levels comparable to or superior to those of AmpB, the reference drug. These results highlight the relevance of their structural features, which likely enhance interactions with parasite-specific molecular targets.

Among them, compound **19** displayed the most striking activity, consistently inhibiting parasite replication across the three species, with reductions ranging from 61.0 to 71.9%. This broad-spectrum efficacy is particularly relevant since therapeutic outcomes in leishmaniasis are often species-dependent.^43^ This compound presents a CF_3_ group in its structure. The introduction of fluorine atoms into bioactive structures is particularly advantageous, as it tends to enhance lipophilicity and, consequently, improve absorption, distribution, and biological performance *in vivo*. The presence of the trifluoromethyl group (−CF_3_) is especially relevant, since this substituent contributes to higher molecular stability and lipophilicity due to its strong electron-withdrawing character.^44^ The presence of a lipophilic substituent within compound **19**’s scaffold may facilitate its passage through host and parasite membranes, favoring accumulation in the parasitophorous vacuole. Lipophilicity is a well-recognized determinant of antileishmanial activity, as it promotes membrane partitioning and can trigger mitochondrial dysfunction.^45,46^

Compound **16** also demonstrated high potency, especially against *L. braziliensis* (72.6%) and *L. infantum* (62.7%), reinforcing its potential as a candidate for tegumentary and visceral leishmaniasis. Its electron-donating substituents may contribute to redox cycling and ROS generation, overwhelming parasite antioxidant defenses and leading to mitochondrial damage and death.^47,48^

By contrast, compound **13** showed species-selective activity, with remarkable efficacy against *L. infantum* (64.9% reduction). Its aromatic substitutions may favor π-π stacking interactions with parasite biomolecules, such as enzymes involved in sterol biosynthesis or DNA topology.^49^ The preferential activity against *L. infantum* suggests that its mechanism is linked to pathways particularly critical for this species’ intracellular survival.

Although compounds **16**, **19**, and **13** produced marked reductions in infection parameters in macrophage assays compared with AmpB (72.5, 42.6, and 69.9% reductions against *L. amazonensis*, *L. braziliensis*, and *L.* *infantum*, respectively), their activity was observed at higher molar concentrations than amphotericin B. Therefore, these compounds cannot be considered more potent than the reference drug on a molar basis; instead, they exhibit promising activity profiles with improved selectivity indices.

In contrast, compounds **18** and **20** showed only moderate reductions (≤59.9%), possibly due to steric hindrance or unfavorable hydrophilic/lipophilic balance, which could impair their ability to cross host and parasite membranes. These findings underscore the importance of structure–activity relationships and suggest that fine-tuning electronic properties, steric accessibility, and lipophilicity may further enhance their biological activity.

The comparative data with AmpB also suggest distinct or complementary mechanisms of action. While AmpB primarily acts by binding to ergosterol-like sterols in parasite membranes,^15^ based on the observed biological activity and *in silico* predictions, compounds **16** and **19** may be associated with mechanisms involving mitochondrial function and redox processes. However, no direct experimental evidence was obtained in this study to confirm a specific mechanism of action, and further mechanistic investigations will be required to validate these hypotheses.

Taken together, the comparative structure–activity analysis indicates that lipophilicity, redox-active substituents, and aromatic groups are key determinants of efficacy within this series. Compounds **16**, **19**, and **13** thus emerge as promising leads for further optimization, with efficacy profiles comparable or superior to AmpB, particularly against *L. braziliensis*, a species notoriously difficult to treat.^50^ Further investigations into their mechanism of action, cytotoxicity profiles, and *in vivo* efficacy will be crucial steps toward their development as novel candidates for leishmaniasis chemotherapy.

Despite these promising findings, this study has some limitations. First, the evaluation was restricted to *in vitro* assays, which may not fully replicate the complexity of host–parasite interactions and pharmacokinetic barriers *in vivo*. Second, while the comparative structure–activity analysis trends provide strong hypotheses for the mechanism of action, these remain speculative and require confirmation through molecular and biochemical assays (e.g., mitochondrial activity, ROS generation, and target identification). Third, cytotoxicity was assessed only in murine macrophages; a broader evaluation using additional mammalian cell types would strengthen the safety of these compounds. Finally, no pharmacokinetic or stability studies were conducted, which are essential for translating these molecules into potential therapeutic candidates.

### 3.3 *In Silico* Results

*In silico* studies were conducted to predict the electronic and ADMET (absorption, distribution, metabolism, excretion, and toxicity) properties of compounds **13**, **16**, **18**, **19**, and **20**. The results are summarized in Table 6.

**Table 6:** Quantum Chemical Descriptors (in eV) and ADMET Properties^a^,^b^

	**quantum chemical descriptors**					**ADMET**				
**compounds**	**HOMO**	**LUMO**	**E**_**g**_	**IP**	**EA**	**LogP**	**TPSA**	**GI**	**P-gp**	**CYP1A2**
**13**	–5.54	–1.33	4.21	5.54	1.33	3.36	47.56	high	no	no
**16**	–5.69	–1.47	4.22	5.69	1.47	3.61	47.56	high	no	no
**18**	–5.28	–1.32	3.96	5.28	1.32	3.29	56.79	high	no	no
**19**	–6.71	–2.09	4.62	6.71	2.09	4.35	29.10	high	no	yes
**20**	–6.35	–1.85	4.50	6.35	1.85	4.23	29.10	high	no	yes

a HOMO (Highest Occupied Molecular Orbital), LUMO (Lowest Unoccupied Molecular Orbital), E_g_ (HOMO – LUMO): energy gap, IP (−HOMO): ionization potential, EA (−LUMO): electron affinity.

b LogP (logarithm of octanol–water partitioning coefficient), TPSA (Topological Polar Surface Area), GI (Gastrointestinal absorption), P-gp (P-glycoprotein substrate), CYP1A2 (Cytochrome P450 1A2) inhibitor.

The chemical reactivity of the compounds was inferred from their energy gap (E_g_) values. Accordingly, compound **18** is the most chemically reactive, with an E_g_ of 3.96 eV, whereas compound **19** is the most stable and least reactive, with an E_g_ of 4.62 eV. As reported in the literature, a lower ionization potential (IP) and a higher electron affinity (EA) indicate that a chemical species behaves as an electron donor and electron acceptor, respectively. Therefore, the lower IP and EA values presented in Table 6 suggest that compounds **13**, **16**, and **18** act as electron donors in chemical reactions, functioning as reducing agents. In contrast, compounds **19** and **20** may act as electron acceptors due to their higher IP and EA values, thus behaving as oxidizing agents in redox processes.

For better visualization of the charge distribution and the HOMO–LUMO densities, Figure 3 presents the Molecular Electrostatic Potential (MEP) map alongside these orbital density plots.

**Figure 3:**
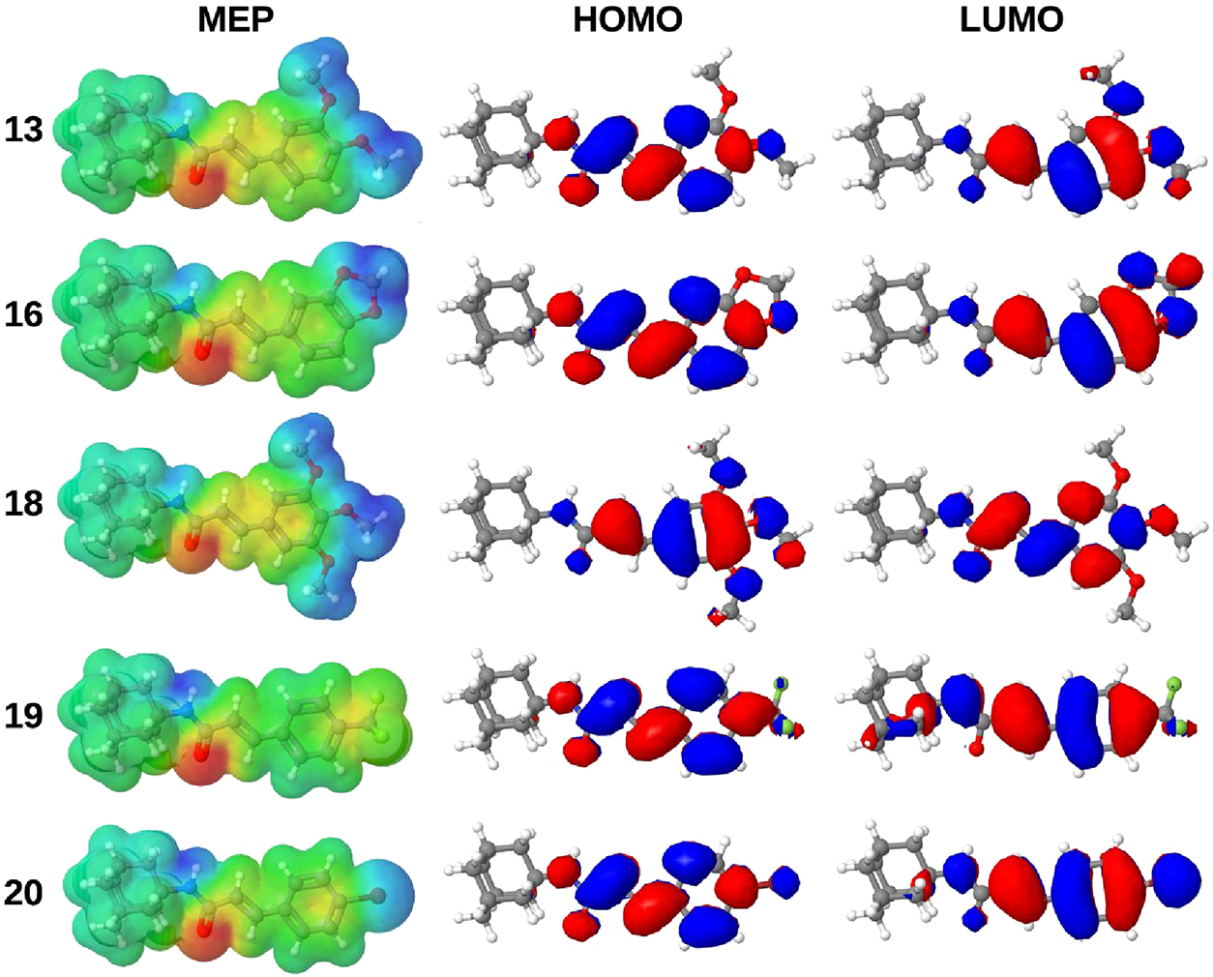
Representation of the molecular electrostatic potential (MEP) and HOMO and LUMO densities of compounds **13**, **16**, **18**, **19**, and **20** at the B3LYP/6–311G­(d,p) level. For MEP, negative and positive charges range from red to blue, respectively.

According to the MEP diagram (Figure 3), in all compounds, regions of high electron density (red) are located around the oxygen atom of the carboxamide group, indicating sites susceptible to electrophilic attack. Compounds **13**, **16**, and **18** display positive regions (blue) around the substituents attached to the phenyl ring and on the nitrogen atom of the carboxamide group, which are likely sites for nucleophilic attack. In compound **19**, the CF_3_ group withdraws electron density from the phenyl ring, rendering it partially neutral (green) and contributing to its hydrophobic character. Compound **20** exhibits a light positive charge density (light blue) around the bromine atom, reflecting the weaker electron-withdrawing effect of Br compared to CF_3_. The regular charge distribution of compound **20** also indicates partial hydrophobicity.

As previously discussed, compounds **13**, **16**, and **18** may act as electron donors, whereas compounds **19** and **20** may function as electron acceptors. Figure 3 shows the HOMO and LUMO density distributions, highlighting the probable regions for oxidation and reduction reactions. Oxidation involves electron removal from the HOMO, while reduction corresponds to electron addition to the LUMO. No HOMO density is observed in the adamantyl group of any compound, but LUMO density appears in this group for compounds **19** and **20**, suggesting a possible reduction in this region. Overall, both HOMO and LUMO densities are primarily localized on the cinnamamide group with a similar pattern across all molecules.

The ADMET properties of the compounds were predicted using the SwissADME Web server (http://www.swissadme.ch/index.php, accessed on October 20, 2025) to better understand their biological activity (Table 5). Compounds **19** (LogP = 4.35) and **20** (LogP = 4.23) exhibit higher lipophilicity and lower TPSA values, indicating greater membrane permeability and absorption. These properties, consistent with the MEP results, likely contribute to their lower IC_50_ values due to enhanced membrane penetration and accumulation. All compounds show high gastrointestinal absorption and are not predicted to be P-glycoprotein (P-gp) substrates, suggesting good oral bioavailability. The CYP1A2 enzyme, involved in drug metabolism, is predicted to be inhibited by compounds **19** and **20** but not by compounds **13**, **16**, and **18**. This aligns with literature reports that hydrophobic molecules with high LogP values are typical CYP1A2 substrates.^51^ Consequently, compounds **19** and **20** may exhibit increased plasma levels, potentially enhancing therapeutic efficacy but also the risk of side effects. Importantly, none of the compounds are predicted to be mutagenic or tumorigenic.

## 4 Conclusion

In this study, a new series of cinnamic acid–amantadine amides was successfully synthesized and characterized. The biological evaluation revealed that several derivatives, particularly compounds **13**, **16**, and **19**, possess moderate and selective activity against *Leishmania* species, notably *L. braziliensis*. The incorporation of the adamantyl moiety, combined with electron-donating or electron-withdrawing substituents on the aromatic ring, proved essential for enhancing leishmanicidal potency and selectivity. The trifluoromethyl-substituted compound **19** emerged as the most promising candidate, exhibiting broad-spectrum activity and favorable selectivity indices. Altogether, the results support the concept that hybridization of cinnamic acid and amantadine pharmacophores can yield potent and safer antileishmanial agents. *In silico* studies based on quantum chemistry and ADMET analyses were conducted to investigate the electronic properties and biological activities of the most promising compounds. The quantum chemical results indicate that all compounds are chemically stable. Compounds **13**, **16**, and **18** act as electron donors, while compounds **19** and **20** function as electron acceptors. The higher LogP (high lipophilicity) and lower TPSA values predicted in ADMET analyses indicate that compounds **19** and **20** permeate the membrane more effectively than the other compounds, which may be related to the biological activity observed at the experimental level. Overall, the ADMET analyses indicate that the compounds exhibit enhanced bioavailability, permeability, and membrane penetration, and are nontoxic. Further studies, including mechanistic investigations and *in vivo* assays, are warranted to confirm their therapeutic potential and to advance these compounds toward preclinical evaluation.

## Supplementary Material



## Notes

Spectroscopic and spectrometric data for the synthesized cinnamic acid–amantadine amides (PDF)

## References

[1] Burza S., Croft S. L., Boelart M. (2018). Leishmaniasis. Lancet.

[2] Serafim T. D., Coutinho-Abreu I. V., Dey R., Kissinger R., Valenzuela J. G., Oliveira F., Kamhawi S. (2021). Leishmaniasis: the
act of transmission. Trends Parasitol..

[3] Leishmaniasis. World Health Organization, https://www.who.int/news-room/fact-sheets/detail/leishmaniasis.

[4] Leishmania: an urgent need for new treatments eBioMedicine, 2023 87 104440 10.1016/j.ebiom.2023.104440. 36653110 PMC9873646

[5] Reithinger R., Dujardin J.-C., Louzir H., Pirme C. (2007). Cutaneous
leishmaniasis. Lancet.

[6] de
Vries H. J. C., Schallig H. D. (2022). Cutaneous leishmaniasis: A 2022 updated
narrative review into diagnosis and management developments. Am. J. Clin. Dermatol..

[7] Cutaneous leishmaniasis . Developing safer and shorter treatments for a disfiguring and stigmatizing disease. Drugs for Neglected Diseases initiative (DNDi). https://dndi.org/diseases/cutaneous-leishmaniasis/.

[8] Scarpini S., Dondi A., Totaro C., Biagi C., Melchionda F., Zama D., Pierantoni L., Gennari M., Campagna C., Prete A., Lanari M. (2022). Visceral leishmaniasis:
Epidemiology,
diagnosis, and treatment regimens in different geographical areas
with a focus on pediatrics. Microorganisms.

[9] Duarte A. G. S., Werneck G. L., Lelis S. F., Mendonça T. S., Vasconcelos D. D., Gontijo T. S., Santos Á. O., Donato L. E., Belo V. S. (2025). An updated systematic review with
meta-analysis and meta-regression of the factors associated with human
visceral leishmaniasis in the Americas. Infect.
Dis. Poverty.

[10] Visceral Leishmaniasis . Symptoms, Transmission, and Current Treatments for Visceral Leishmaniasis. Drugs for Neglected Diseases initiative (DNDi). https://dndi.org/diseases/visceral-leishmaniasis/.

[11] Frézard F., Demichelli C., Ribeiro R. R. (2009). Pentavalent antimonials: New perspectives
for old drugs. Molecules.

[12] Lindoso J. A. L., Costa J. M. L., Queiroz I. T., Goto H. (2012). Review of
the current
treatments for leishmaniasis. Res. Rep. Trop.
Med..

[13] Sundar S., Singhi J., Singh V. K., Agrawal N., Kumar R. (2024). Current and
emerging therapies for the treatment of leishmaniasis. Expert Opin. Orphan Drugs.

[14] Mann S., Frasca K., Scherrer S., Henao-Martínez A. F., Newman S., Ramanan P., Suarez J. A. (2021). A review of leishmaniasis:
Current knowledge and future directions. Curr.
Trop. Med. Rep..

[15] Croft S. L., Coombs G. H. (2003). Leishmaniasis – Current chemotherapy
and recent
advances in the search of novel drugs. Trends
Parasitol..

[16] Shmueli M., Bem-Shimol S. (2024). Review of leishmaniasis treatment: Can we see the forest
through the trees?. Pharmacy.

[17] Majumder N., Banerjee A., Saha S. (2023). A Review on
new natural and synthetic
anti-leishmanial chemotherapeutic agents and current perspective of
treatment approaches. Acta Trop..

[18] Gervazoni L. F. O., Barcellos G. B., Ferreira-Paes T., Almeida-Amaral E. E. (2020). Use of
natural products in leishmaniasis chemotherapy: An Overview. Front. Chem..

[19] Cortes S., Sousa C. B., Morais T., Lago J., Campino L. (2020). Potential
of the natural products against leishmaniasis in Old World –
A Review of *in vitro* studies. Pathog. Global Health.

[20] Afonso R. C., Yien R. M. K., Siqueira L. B. O., Simas N. K., Matos A. P. S., Ricci-Júnior E. (2023). Promising
natural products for the
treatment of cutaneous leishmaniasis: A Review of *in vitro* and *in vivo* studies. Exp.
Parasitol..

[21] Ruwizhi N., Aderibigbe B. A. (2020). Cinnamic
acid derivatives and their biological efficacy. Int. J. Mol. Sci..

[22] De P., Baltas M., Bedos-Belval F. (2011). Cinnamic acid
derivatives as anticancer
agents – A review. Curr. Med. Chem..

[23] França S. B., Correia P. R. S., Castro I. B. D., Silva Júnior E. F., Barros M. E. S. B., Lima D. J. P. (2021). Synthesis, applications and structure-activity
relationship (SAR) of cinnamic acid derivatives: A Review. Res. Soc. Dev..

[24] de
Morais M. C., Medeiros G. A., Almeida F. S., Rocha J. C., Perez-Castillo Y., Kessen T. S. L., Souza D. P. (2023). Antileishmanial
activity of cinnamic acid derivatives against *Leishmania
infantum*. Molecules.

[25] Rodrigues M. P., Tomaz D. C., Souza L. Â., Onofre T. S., Menezes W. A., Almeida-Silva J., Suarez-Fontes A. M., Almeida M. R., Silva A. M., Bressan G. C., Vannier-Santos M. A., Fietto J. L. R., Teixeira R. R. (2019). Synthesis
of cinnamic acid derivatives and leishmanicidal activity against *Leishmania braziliensis*. Eur.
J. Med. Chem..

[26] Davies W. L., Grunert R. R., Haff R. F., McGahen J. W., Neumayer E. M., Paulshock M., Watts J. C., Wood T. R., Hermann E. C., Hoffmann C. E. (1964). Antiviral
activity of 1-Adamantanamine (Amantadine). Science.

[27] Maugh T. H. (1976). Amantadine:
an alternative for prevention of influenza. Science.

[28] Rejdak K., Grieb P. (2020). Adamantanes might be protective from COVID-19 in patients with neurological
diseases: Multiple sclerosis, parkinsonism and cognitive impairment. Mult. Scler. Relat. Disord..

[29] Fink K., Nitsche A., Neuman M., Grossegesse M., Eisele K.-H., Danysz W. (2021). Amantadine inhibits
SARS-CoV-2 *in vitro*. Viruses.

[30] Lim S.-Y., Guo Z., Liu P., McKay L. G. A., Storm N., Griffiths A., Qu M. D., Finberg R. W., Somasundaran M., Wan J. P. (2022). Anti-SARS-CoV-2 activity of adamantanes *in
vitro* and in animal models of infection. COVID.

[31] Hubsher G., Haider M., Okun M. S. (2012). Amantadine:
The journey from fighting
flu to treating Parkinson disease. Neurology.

[32] Wu Y., Pons V., Goudet A., Panigai L., Fischer A., Herweg J.-A., Kali S., Davey R. A., Laporte J., Bouclier C., Yousfi R., Aubenque C., Merer G., Gobbo E., Lopez R., Gillet C., Cojean S., Popoff M. R., Clayette P., Le Grand R., Boulogne C., Tordo N., Lemichez E., Loiseau P. M., Rudel T., Sauvaire D., Cintrat J.-C., Gillet D., Barbier J. (2017). ABMA, a small
molecule that inhibits intracellular toxins and pathogens by interfering
with late endosomal compartments. Sci. Rep..

[33] Pomel S., Cojean S., Pons V., Cintrat J.-C., Nguyen L., Vacus J., Pruvost A., Barbier J., Gillet D., Loiseau P. M. (2021). An adamantamine
derivative as a drug candidate for
the treatment of visceral leishmaniasis. J.
Antimicrob. Chemother..

[34] Papanastasiou I., Prousis K. C., Georgikopoulou K., Pavlidis T., Scoulica E., Kolocouris N., Calogeropoulou T. (2010). Design and synthesis of new adamantyl-substituted
antileishmanial ether phospholipids. Bioorg.
Med. Chem. Lett..

[35] Liu J., Obando D., Liao V., Lifa T., Codd R. (2011). The many faces
of the adamantyl group in drug design. Eur.
J. Med. Chem..

[36] Chianna D., Grace A. C., Allen B., Montgomery A. P., Danon J. J., Kassiou M. (2024). Unlocking Therapeutic Potential:
The role of adamantane in drug discovery. Aust.
J. Chem..

[37] Viana L. P. S., Naves G. M., Medeiros I. G., Guimarães A. S., Souza E. S., Santos J. C. C., Freire N. M. L., Aquino T. M., Modolo L. V., de Fátima Â., da Silva C. M. (2024). Synergizing structure
and function: Cinnamoyl hydroxamic acids as potent urease inhibitors. Bioorg. Med. Chem..

[38] Coelho E. A. F., Tavares C. A., Carvalho F. A., Chaves K. F., Teixeira K. N., Rodrigues R. C., Charest H., Matlashewski G., Gazzinelli R. T., Fernandes A. P. (2003). Immune Responses Induced by the *Leishmania
(Leishmania) donovani* A2 Antigen, but Not by
the LACK Antigen, Are Protective against Experimental *Leishmania
(Leishmania) amazonensis* Infection. Infect. Immun..

[39] Becke A. D. (1988). Density-Functional
Exchange-Energy Approximation with Correct Asymptotic Behavior. Phys. Rev. A.

[40] Lee C., Yang W., Parr R. G. (1988). Development of the Colle–Salvetti
Correlation-Energy Formula into a Functional of the Electron Density. Phys. Rev. B.

[41] Neese, F. ORCA—An Ab Initio, DFT, and Semiempirical SCF–MO Package; Max Planck Institute for Bioinorganic Chemistry: Mülheim an der Ruhr, Germany, 2010.

[42] Santos F. S. D., Freitas R. P., Freitas C. S., Mendonça D. V. C., Lage D. P., Tavares G. S. V., Machado A. S., Martins V. T., Costa A. V., Queiroz V. T., de Oliveira M. B., Oliveira F. M., Antinarelli L. M. R., Coimbra E. S., Pilau E. J., da Silva G. P., Coelho E. A. F., Teixeira R. R. (2023). Synthesis of 1,2,3-triazole-containing
methoxylated cinnamides and their antileishmanial activity against
the *Leishmania braziliensis* species. Pharmaceuticals.

[43] Ponte-Sucre A., Gamarro F., Dujardin J.-C., Barrett M. P., López-Vélez R., García-Hernández R., Pountain A. W., Mwenechanya R., Papadopoulou B. (2017). Drug resistance and treatment failure in leishmaniasis:
A 21st century challenge. PLoS Neglected Trop.
Dis..

[44] Novás M., Matos M. J. (2025). The role of trifluoromethyl
and trifluoromethoxy groups
in medicinal chemistry: Implications for drug design. Molecules.

[45] Bhattacharya S. (2025). Unravelling
antileishmanial mechanisms of phytochemicals: From mitochondrial disruption
to immunomodulation. Future Integr. Med..

[46] Scariot D. B., Staneviciute A., Zhu J., Li X., Scott E. A., Engman D. M. (2022). Leishmaniasis and Chagas disease:
Is there hope in
nanotechnology to fight neglected tropical diseases?. Front. Cell. Infect. Microbiol..

[47] Silva-Silva J. V., Moragas-Tellis C. J., Chagas M. S. S., Souza P. V. R., Moreira D. L., Hardoim D. J., Taniwaki N. N., Costa V. F. A., Bertho A. L., Brondani D., Sá F. A. N., da Silva A. J. R. (2022). Carajurin induces apoptosis
in *Leishmania amazonensis* promastigotes
through reactive oxygen species production and mitochondrial
dysfunction. Pharmaceuticals.

[48] Fonseca-Silva F., Inacio J. D. F., Canto-Cavalheiro M. M., Almeida-Amaral E. E. (2011). Reactive
oxygen species production and mitochondrial dysfunction contribute
to quercetin-induced death in *Leishmania amazonensis*. PLoS One.

[49] de
Souza W., Rodrigues J. C. F. (2009). Sterol biosynthesis pathway as target
for anti-trypanosomatid drugs. Interdiscip.
Perspect. Infect. Dis..

[50] Rugani J. N., Quaresma P. F., Gontijo C. F., Soares R. P., Monte-Neto R. L. (2018). Intraspecies
susceptibility of *Leishmania (Viannia) braziliensis* to antileishmanial drugs: Antimony resistance in human isolates
from atypical lesions. Biomed. Pharmacother..

[51] Zhou S. F., Yang L. P., Zhou Z. W., Liu Y. H., Chan E. (2009). Insights into
the Substrate Specificity, Inhibitors, Regulation, and Polymorphisms
and the Clinical Impact of Human Cytochrome P450 1A2. AAPS J..

