# Supplementary material for: Synthesis and Antileishmanial Activity of Cinnamic Acid–Amantadine Amides
Source: ACS Omega. 2026 Mar 11;11(11):17576–88. doi: 10.1021/acsomega.5c11247 (PMC13019179; doi:10.1021/acsomega.5c11247)
Supplement: Supplementary file 1 [file ao5c11247_si_001.pdf]

SYNTHESIS AND ANTILEISHMANIAL ACTIVITY OF CINNAMIC ACID AMANTADINE AMIDES

Érika Basílio Fernandes,[a] Camila Simões de Freitas,[b] Luciana Pereira Silva Viana,[c] Cleiton Moreira da Silva,[c] Natália Assis Guedes,[d] Wanderson Romão,[d] Valdemar Lacerda Jr.,[d] Nayara Araújo dos Santos,[d] Fabrício Marques de Oliveira,[e] Danilo Aniceto da Silva[a], Cristiane Isaac Cerceau,[a] Mariana Belizario de Oliveira,[f] William dos Santos Belarmino,[f] Quesia Helena Campos Serpa,[f] Osmair Vital de Oliveira,[g] Adílson Vidal Costa,[f] Eduardo Antônio Ferraz Coelho,[b*] Róbson Ricardo Teixeira[a*]*

[a]*Grupo de Síntese e Pesquisa de Compostos Bioativos (GSPCB), Departamento de Química, Universidade Federal de Viçosa, Viçosa, MG, Brazil.*[a]

[b]*Programa de Pós-Graduação em Ciências da Saúde: Infectologia e Medicina Tropical, Faculdade de Medicina, Universidade Federal de Minas Gerais, Belo Horizonte, MG, Brazil.*

[c]*Grupo de Estudos em Química Orgânica e Biológica (GEQOB), Departamento de Química, Instituto de Ciências Exatas, Universidade Federal de Minas Gerais, Belo Horizonte, MG, Brazil.*

[d]*Laboratório de Petroleômica e Forense, Departamento de Química, Universidade Federal do Espírito Santo, Vitória, ES, Brazil.*

[e]*Instituto Federal de Educação, Ciência e Tecnologia de Minas Gerais, Ouro Branco, Minas Gerais, Brazil.*

[f]*Grupo de Estudo Aplicado em Produtos Naturais e Síntese Orgânica (GEAPS), Departamento de Química e Física, Universidade Federal do Espírito Santo, Alegre, ES, Brazil.*

[g]*Instituto Federal de São Paulo, 15808-305 Catanduva-SP, Brazil.*

[*]*Corresponding authors*: robsonr.teixeira@ufv.br (RRT); avcosta@hotmail.com (AVC); eduardo ferrazcoelho@yahoo.com.br (EAFC)

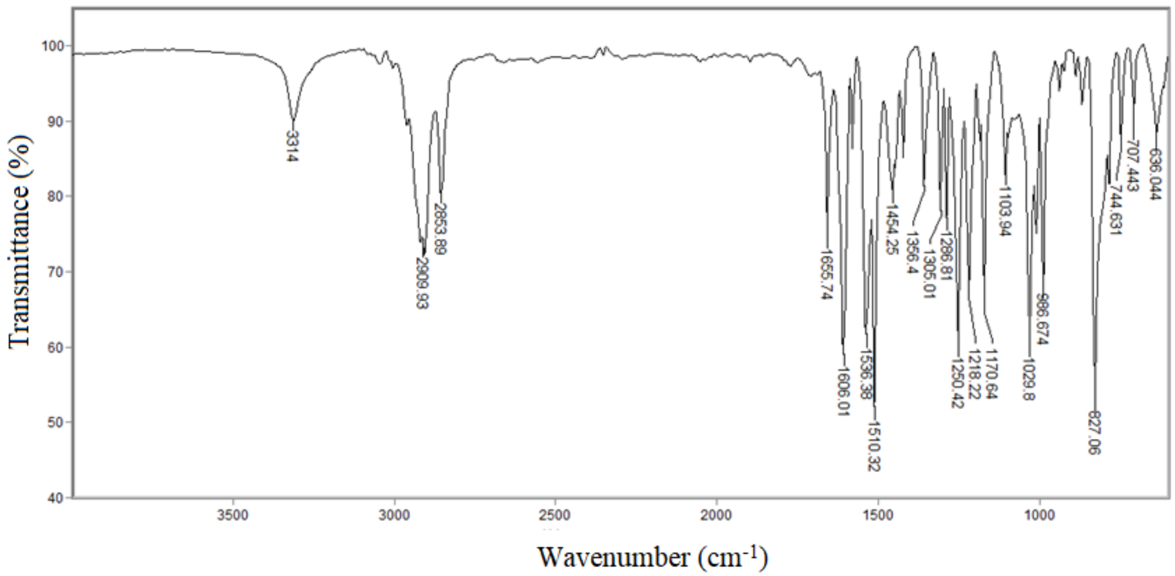

Figure S1. FTIR spectrum (ATR) of (*E*)-*N*-(adamantan-1-yl) cinnamamide (**11**).

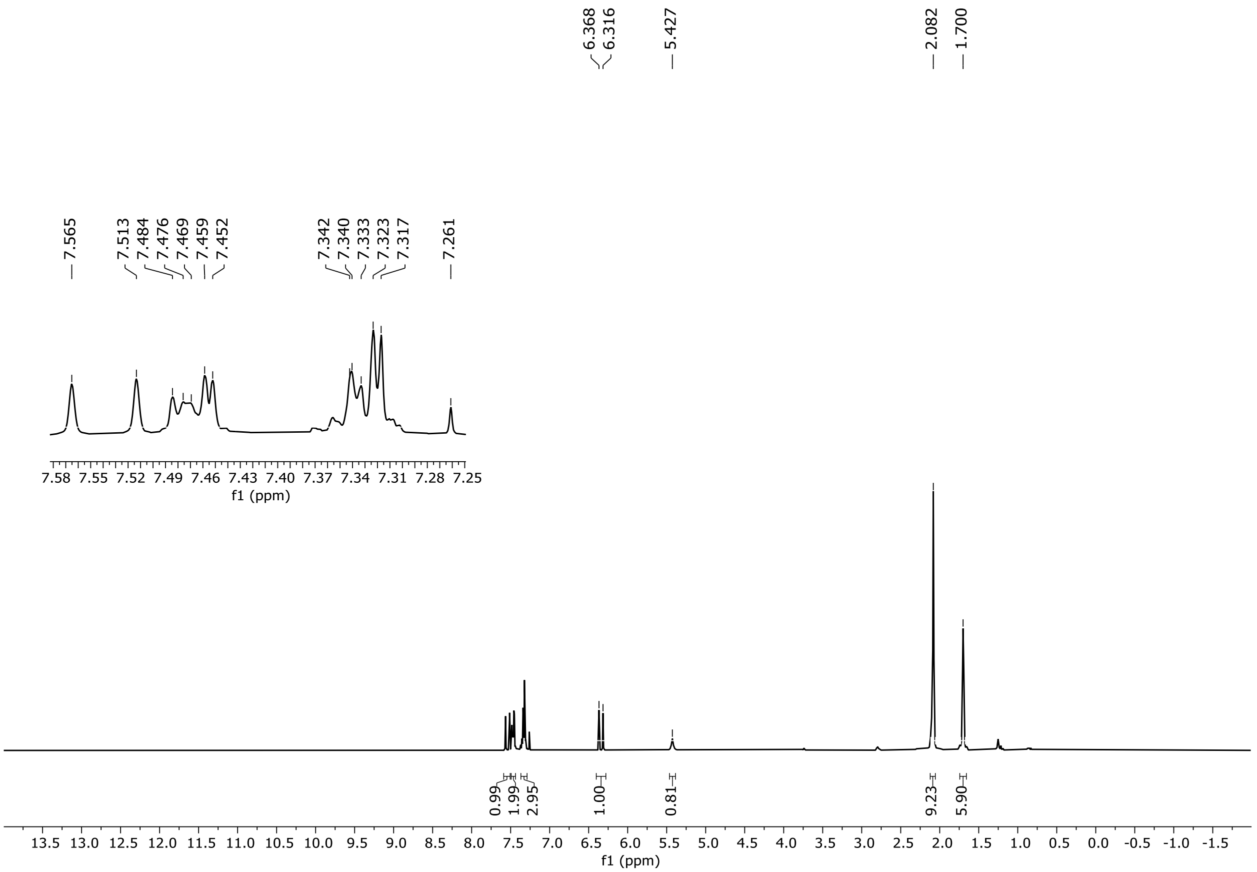

Figure S2. ^{1}H NMR spectrum (300 MHz, $CDCl_3$) of (*E*)-*N*-(adamantan-1-yl) cinnamamide (**11**).

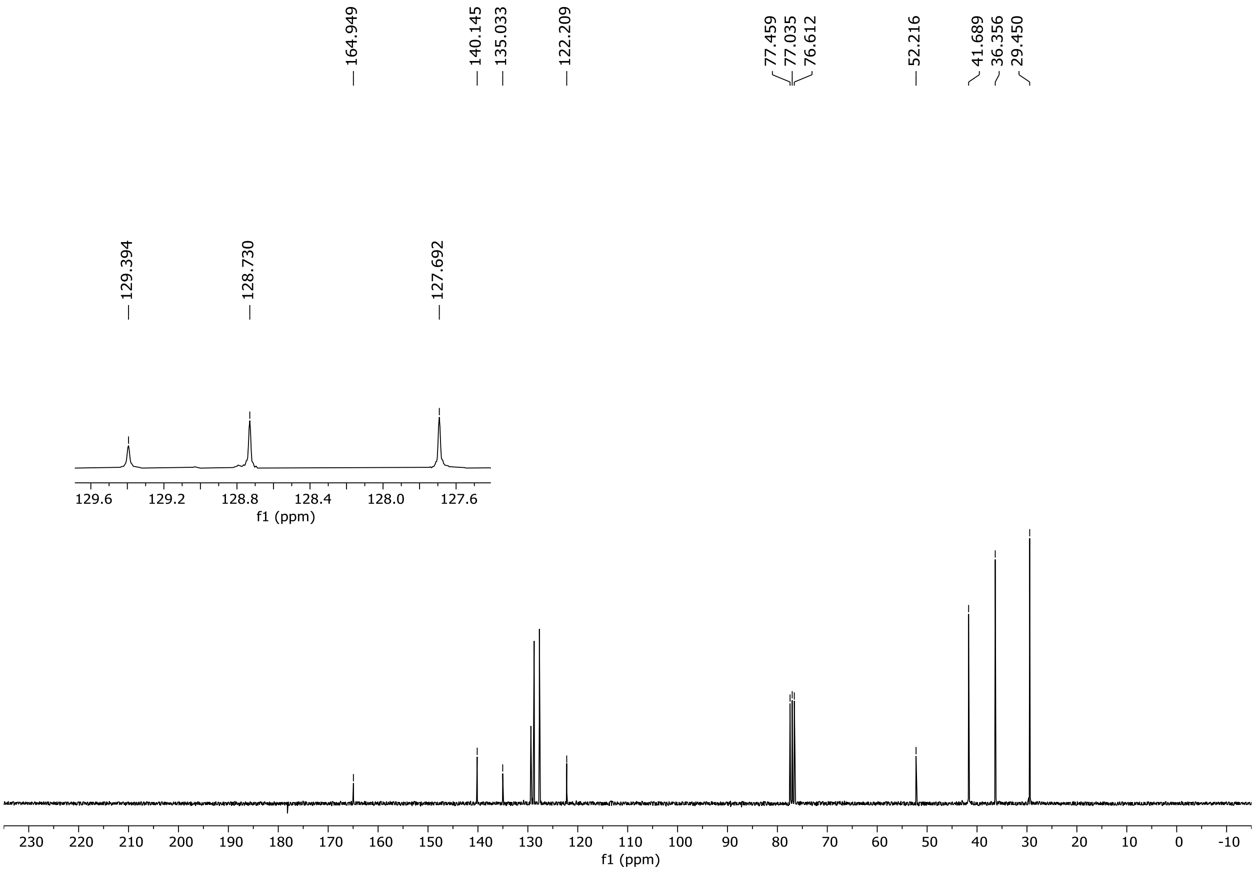

Figure S3. ^{13}C NMR spectrum (75 MHz, $CDCl_3$) of (*E*)-*N*-(adamantan-1-yl) cinnamamide (**11**).

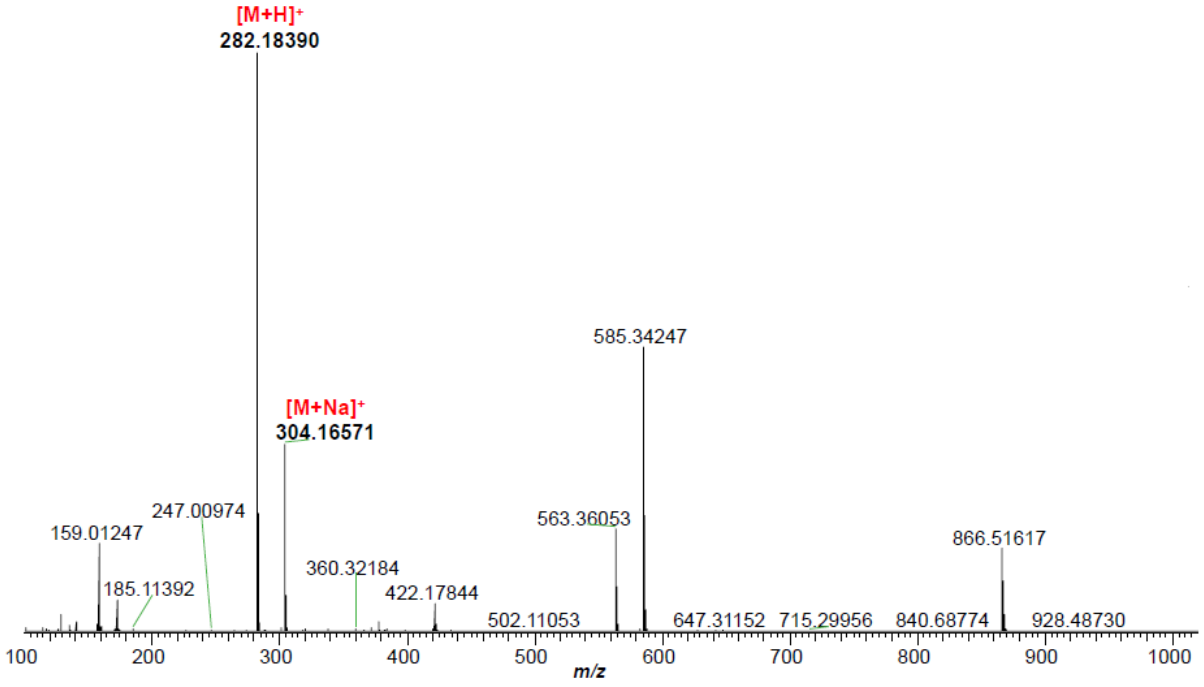

Figure S4. HRMS (ESI) spectrum of (*E*)-*N*-(adamantan-1-yl) cinnamamide (**11**).

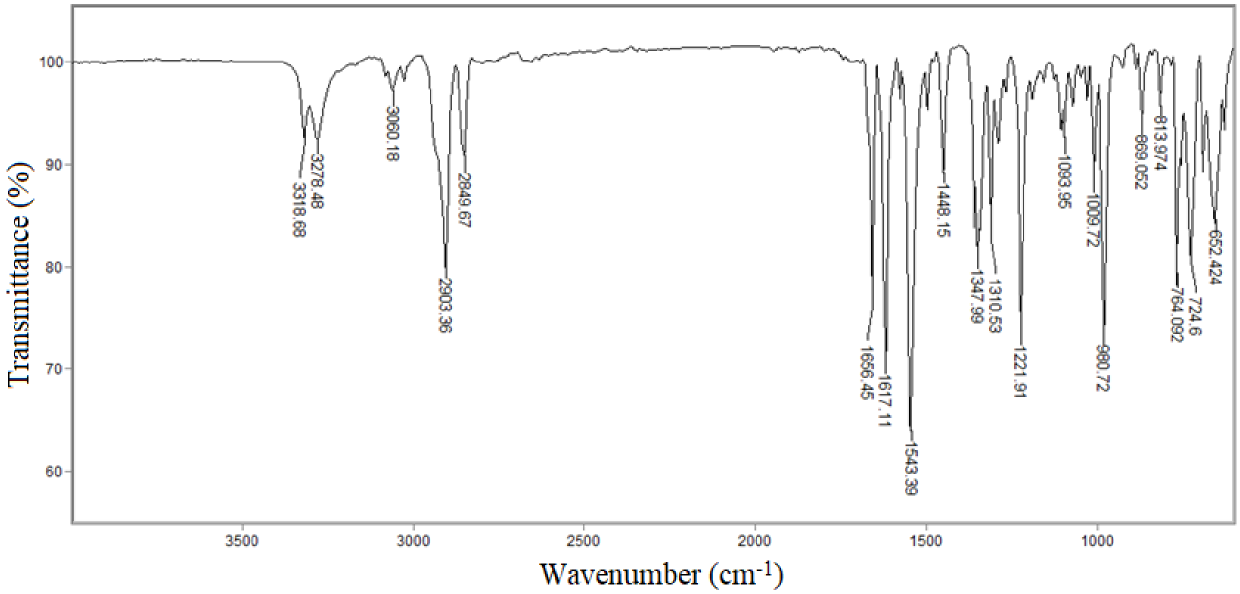

Figure S5. FTIR spectrum (ATR) of (*E*)-*N*-(adamantan-1-yl)-3-(4-methoxyphenyl) acrylamide (**12**).

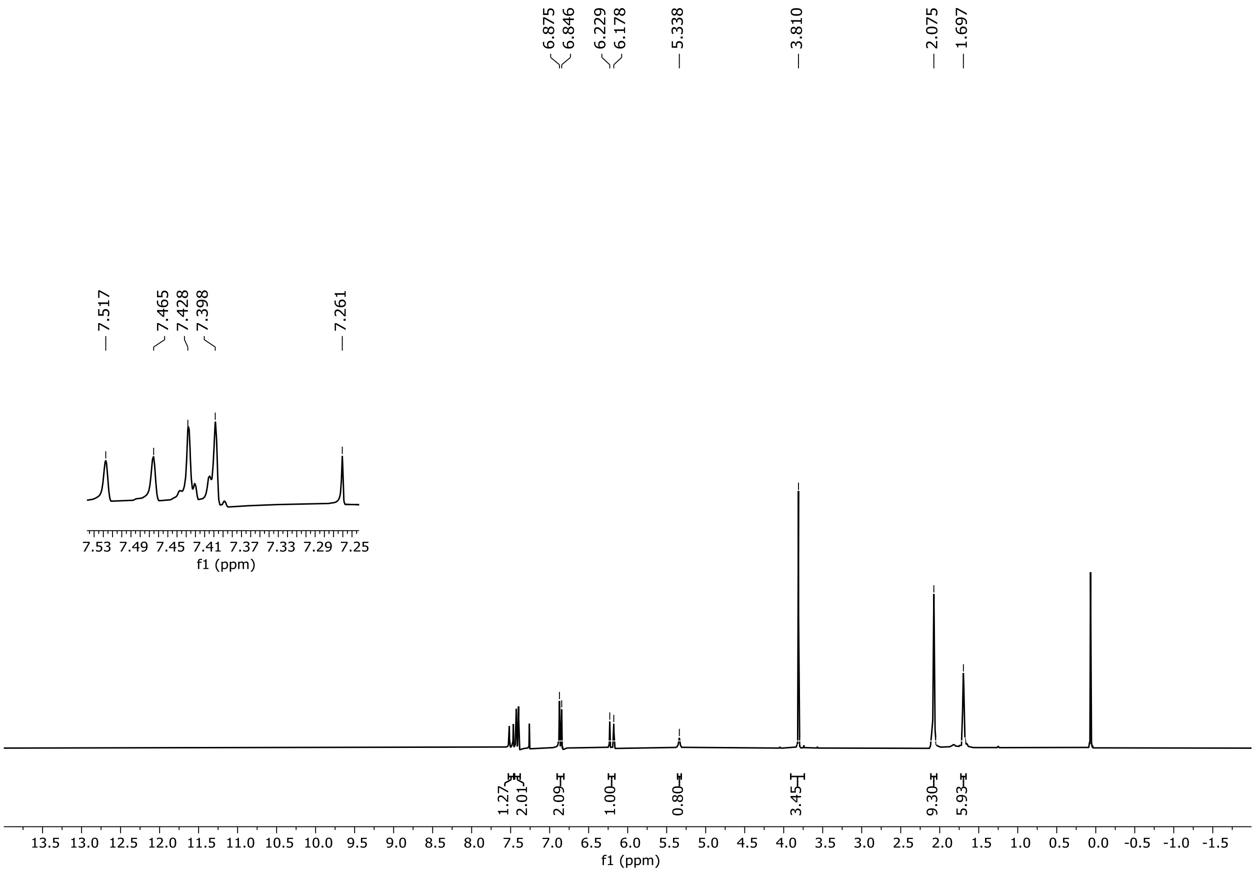

Figure S6. ^{1}H NMR spectrum (300 MHz, $CDCl_3$) of (*E*)-*N*-(adamantan-1-yl)-3-(4-methoxyphenyl) acrylamide (**12**).

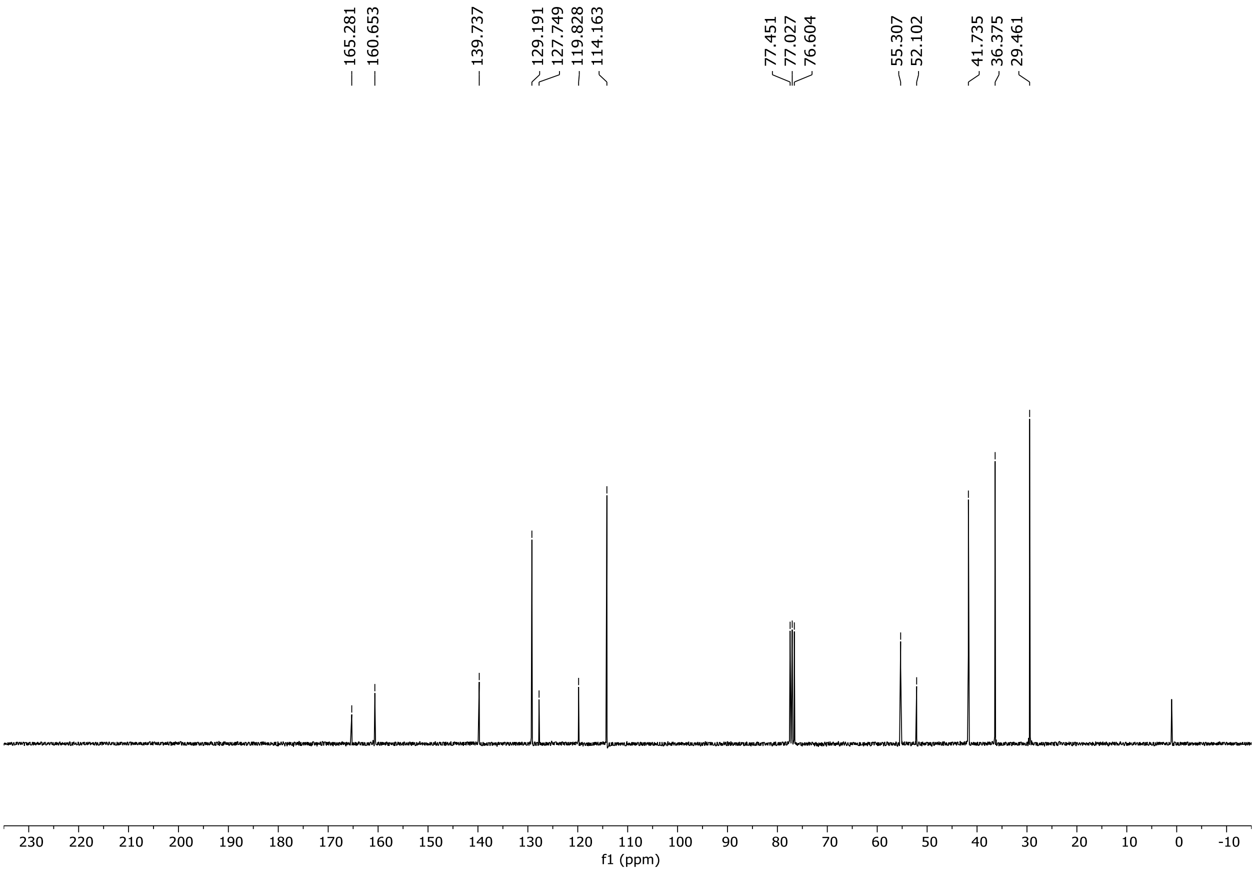

Figure S7. ^{13}C NMR spectrum (75 MHz, $CDCl_3$) of (*E*)-*N*-(adamantan-1-yl)-3-(4-methoxyphenyl) acrylamide (**12**).

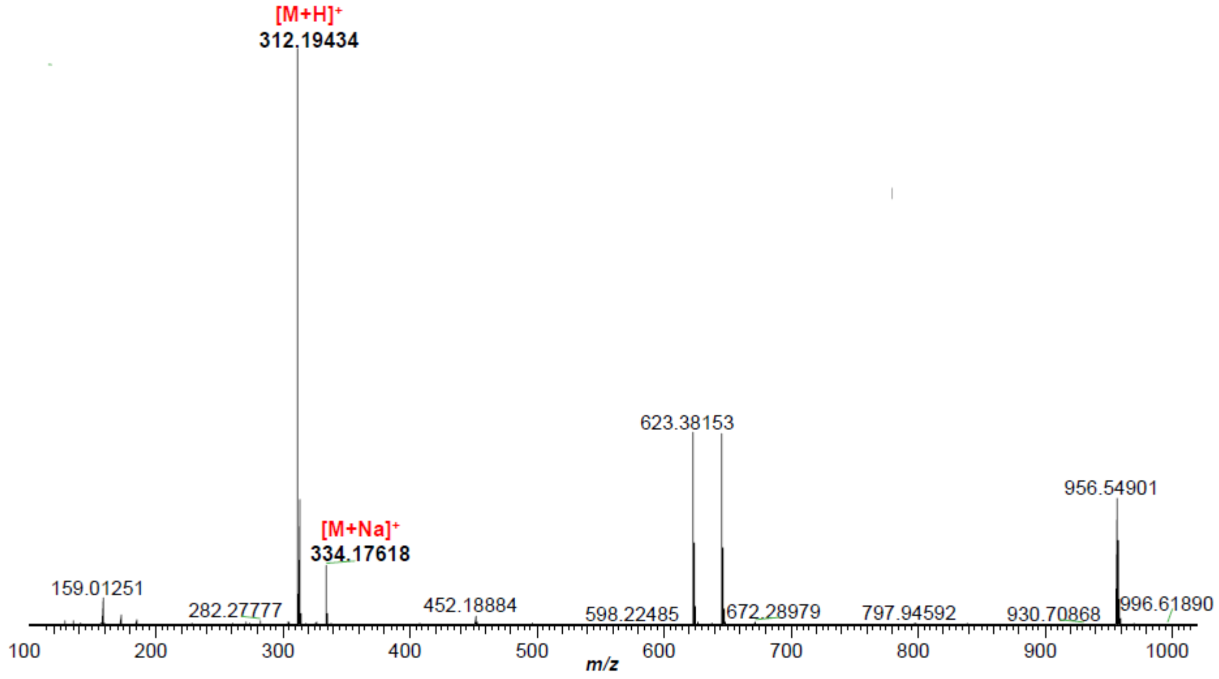

Figure S8. HRMS (ESI) spectrum of (*E*)-*N*-(adamantan-1-yl)-3-(4-methoxyphenyl) acrylamide (**12**).

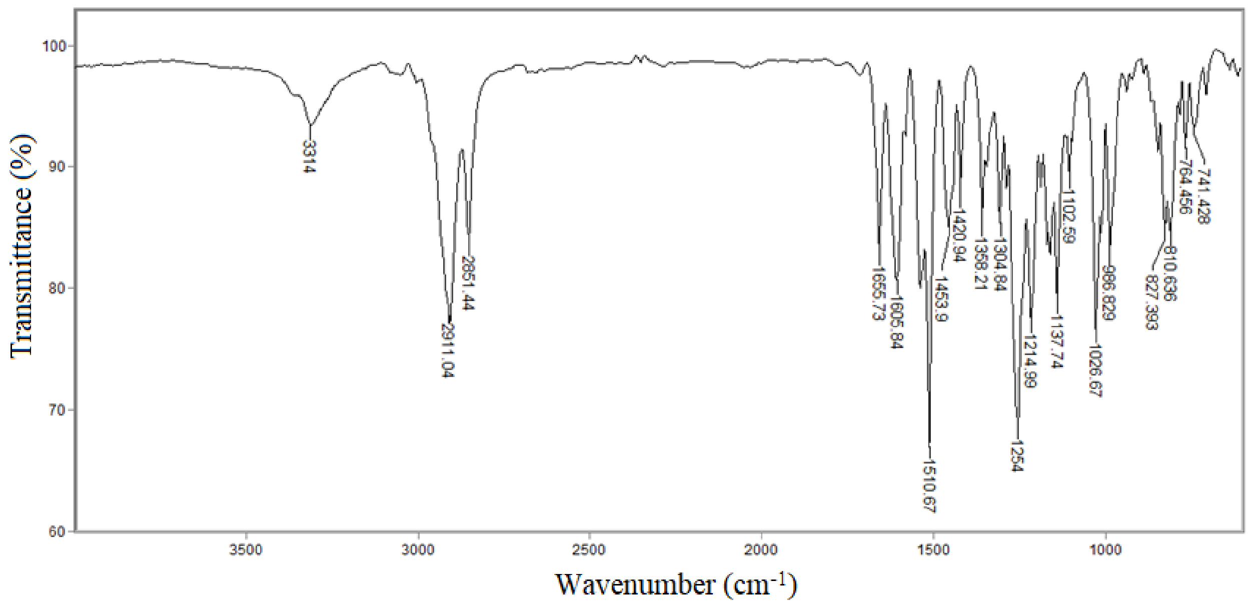

Figure S9. FTIR spectrum (ATR) of (*E*)-*N*-(adamantan-1-yl)-3-(3,4-dimethoxyphenyl) acrylamide (**13**).

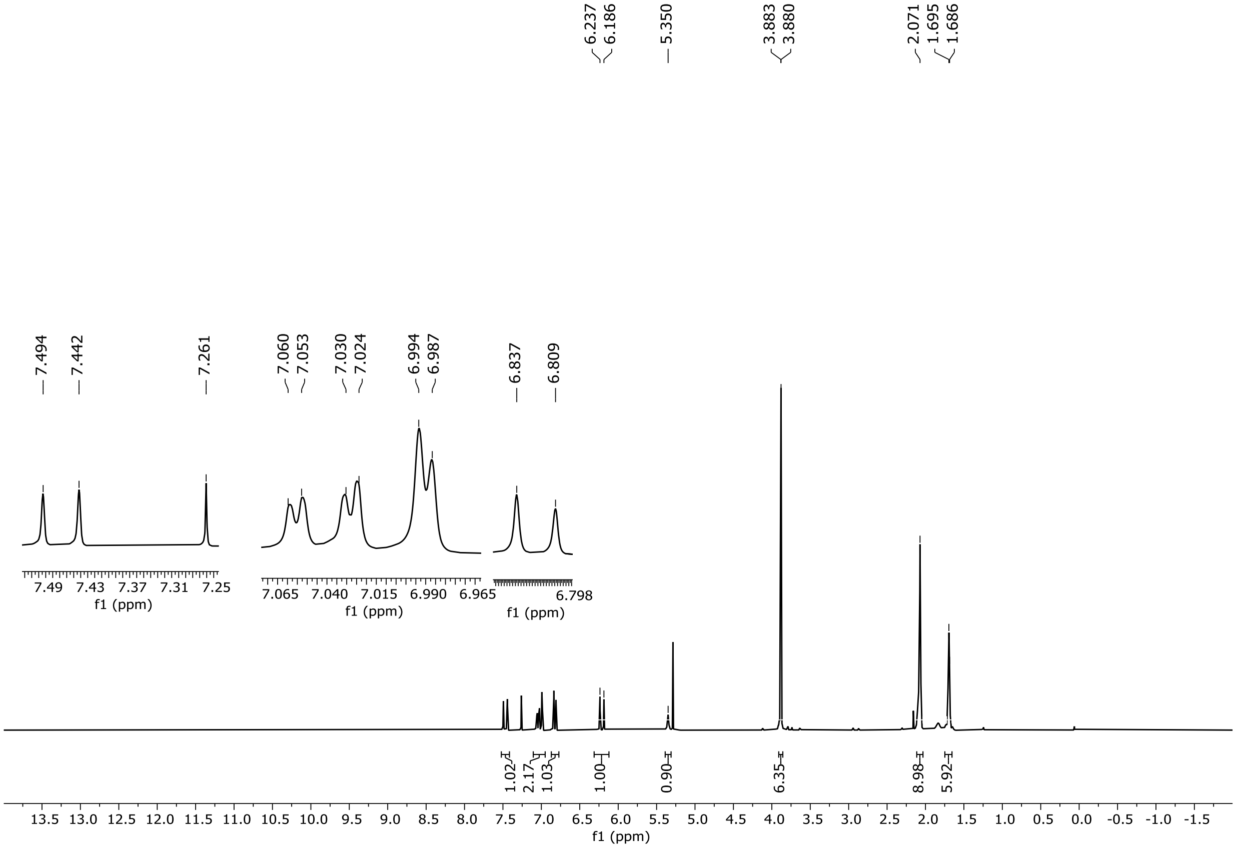

Figure S10. ^{1}H NMR spectrum (300 MHz, $CDCl_3$) of (*E*)-*N*-(adamantan-1-yl)-3-(3,4-dimethoxyphenyl) acrylamide (**13**).

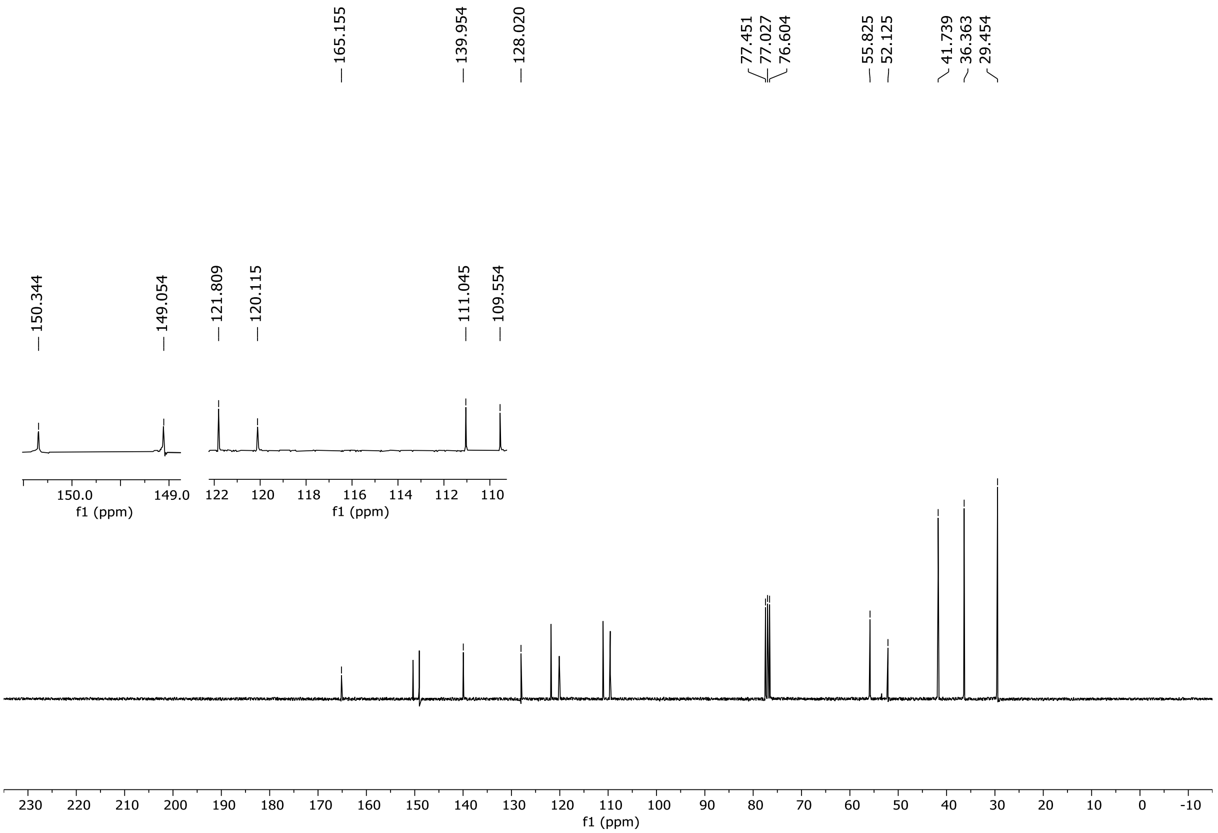

Figure S11. ^{13}C NMR spectrum (75 MHz, $CDCl_3$) of (*E*)-*N*-(adamantan-1-yl)-3-(3,4-dimethoxyphenyl) acrylamide (**13**).

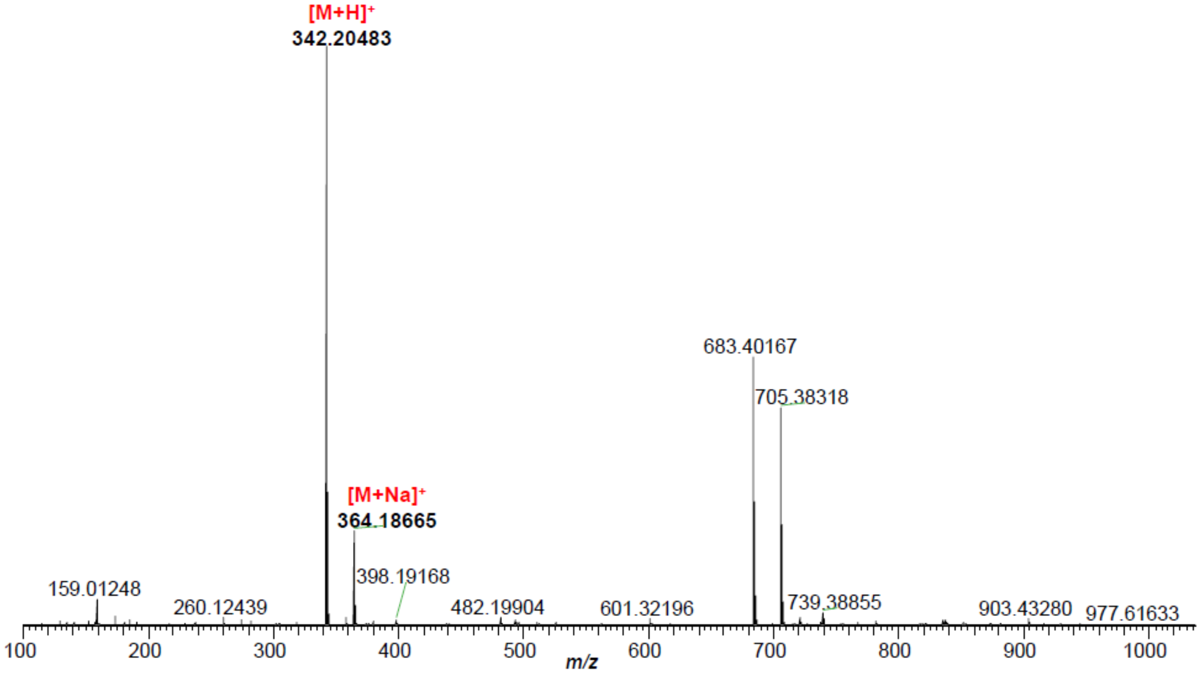

Figure S12. LC-MS spectrum of (*E*)-*N*-(adamantan-1-yl)-3-(3,4-dimethoxyphenyl) acrylamide (**13**).

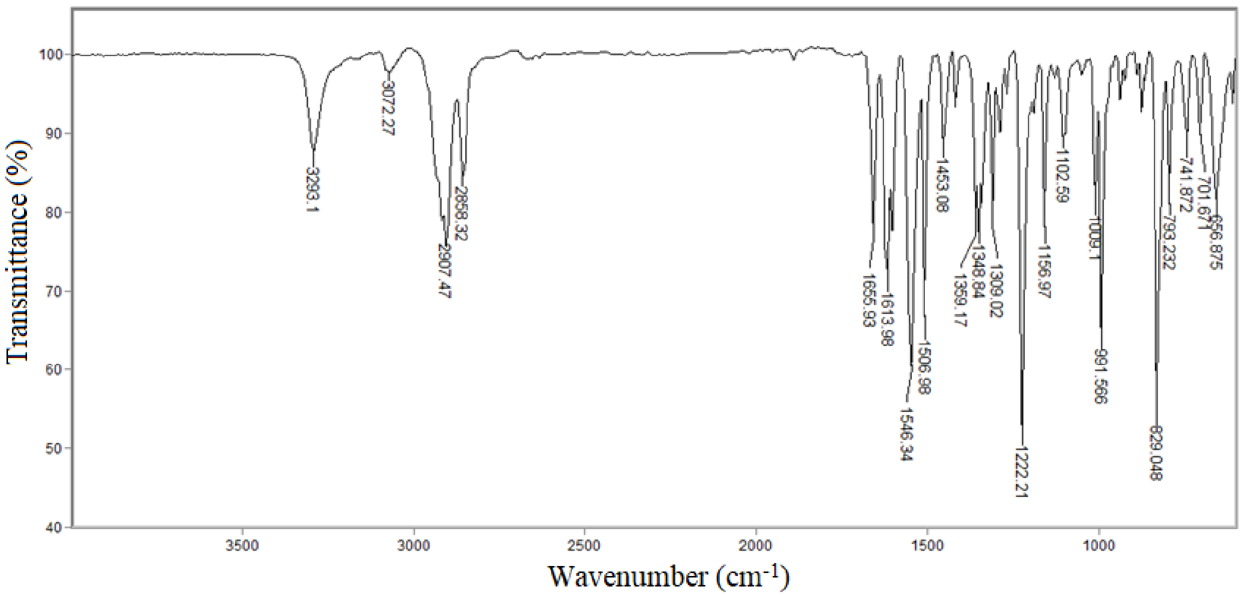

Figure S13. FTIR spectrum (ATR) of (*E*)-*N*-(adamantan-1-yl)-3-(4-fluorophenyl) acrylamide (**14**).

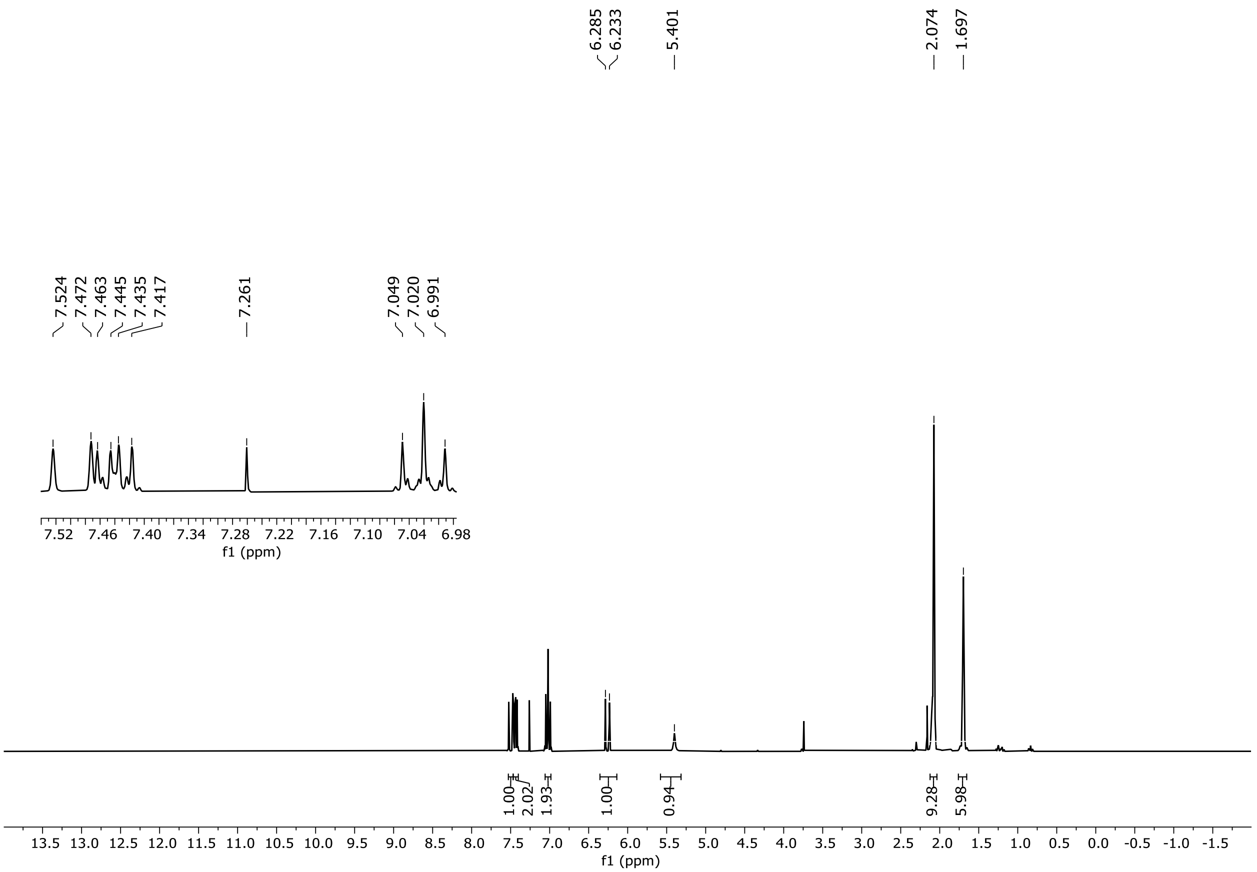

Figure S14. ^{1}H NMR spectrum (300 MHz, $CDCl_3$) of (*E*)-*N*-(adamantan-1-yl)-3-(4-fluorophenyl) acrylamide (**14**).

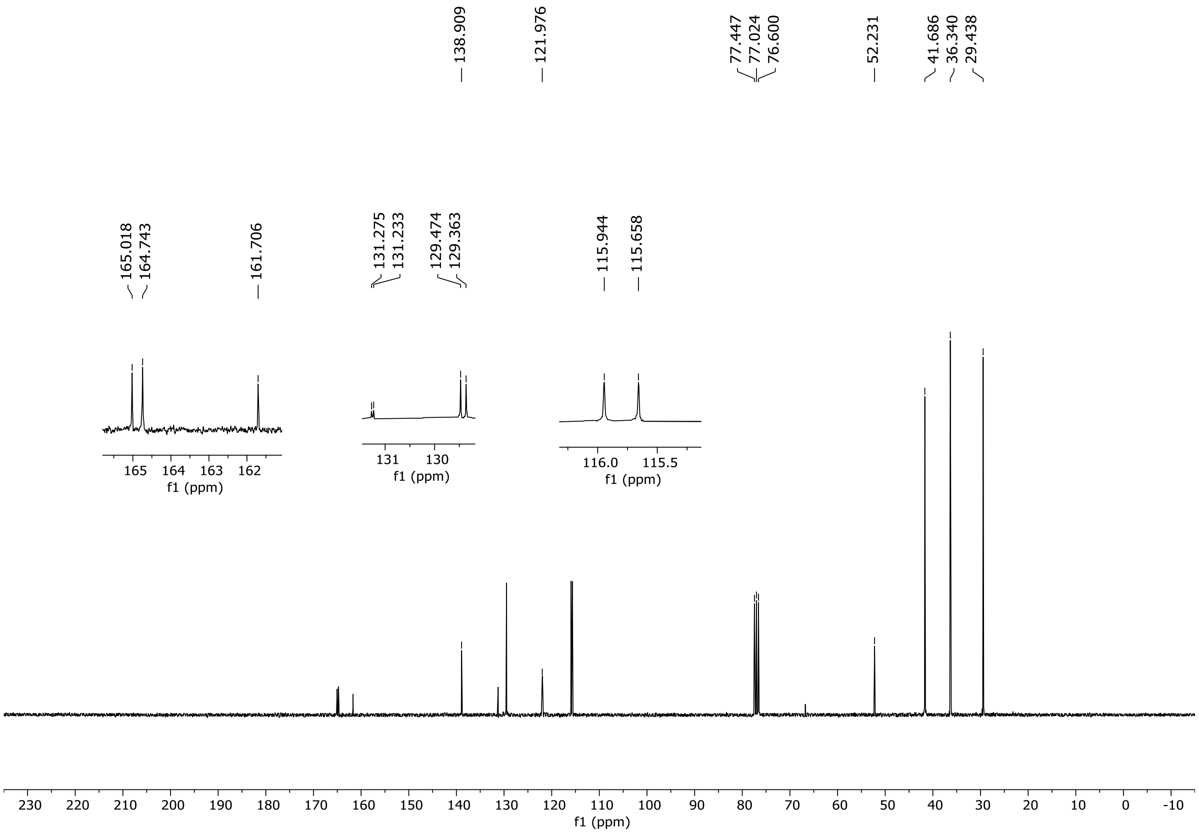

Figure S15. ^{13}C NMR spectrum (75 MHz, $CDCl_3$) of (*E*)-*N*-(adamantan-1-yl)-3-(4-fluorophenyl) acrylamide (**14**).

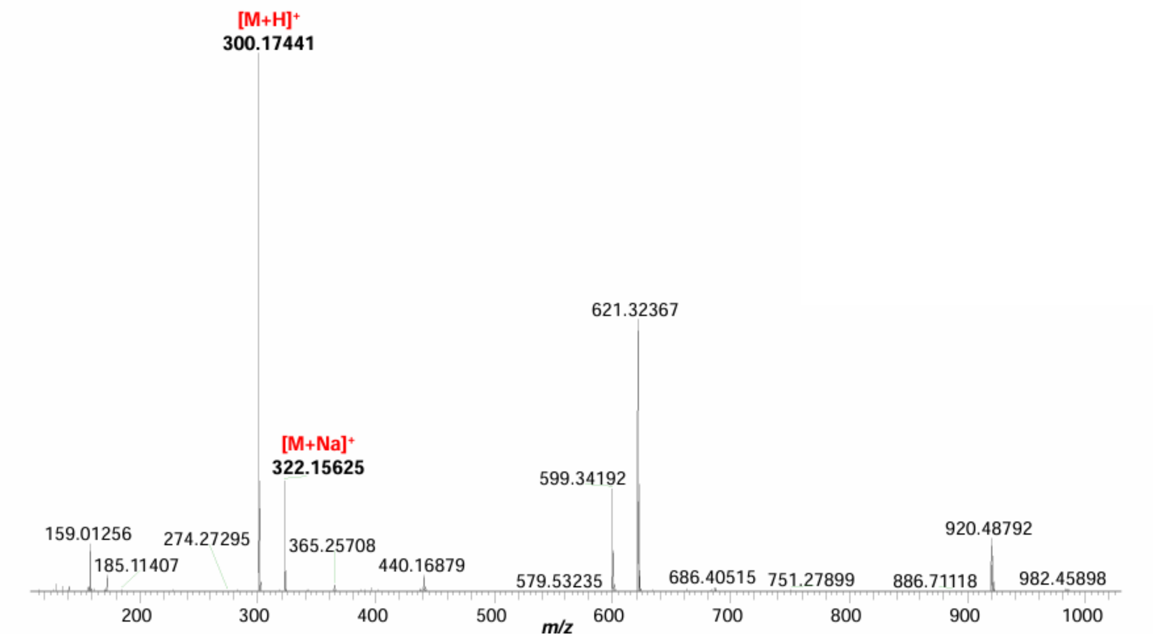

Figure S16. HRMS (ESI) spectrum of (*E*)-*N*-(adamantan-1-yl)-3-(4-fluorophenyl) acrylamide (**14**).

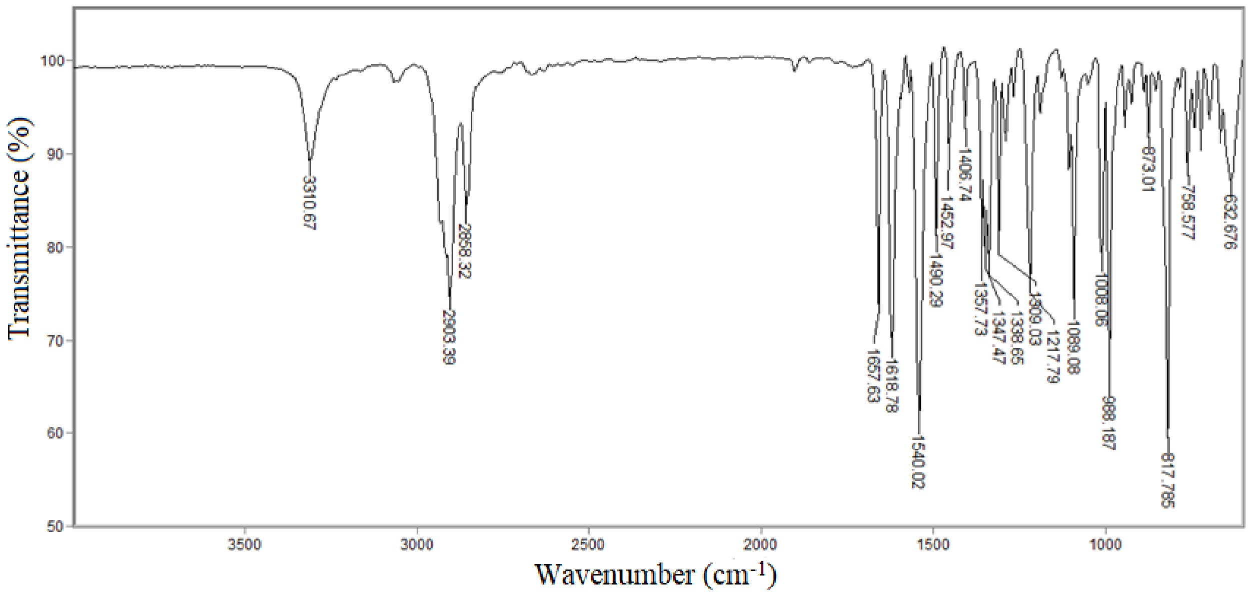

Figure S17. FTIR spectrum (ATR) of (*E*)-*N*-(adamantan-1-yl)-3-(4-chlorophenyl) acrylamide (**15**).

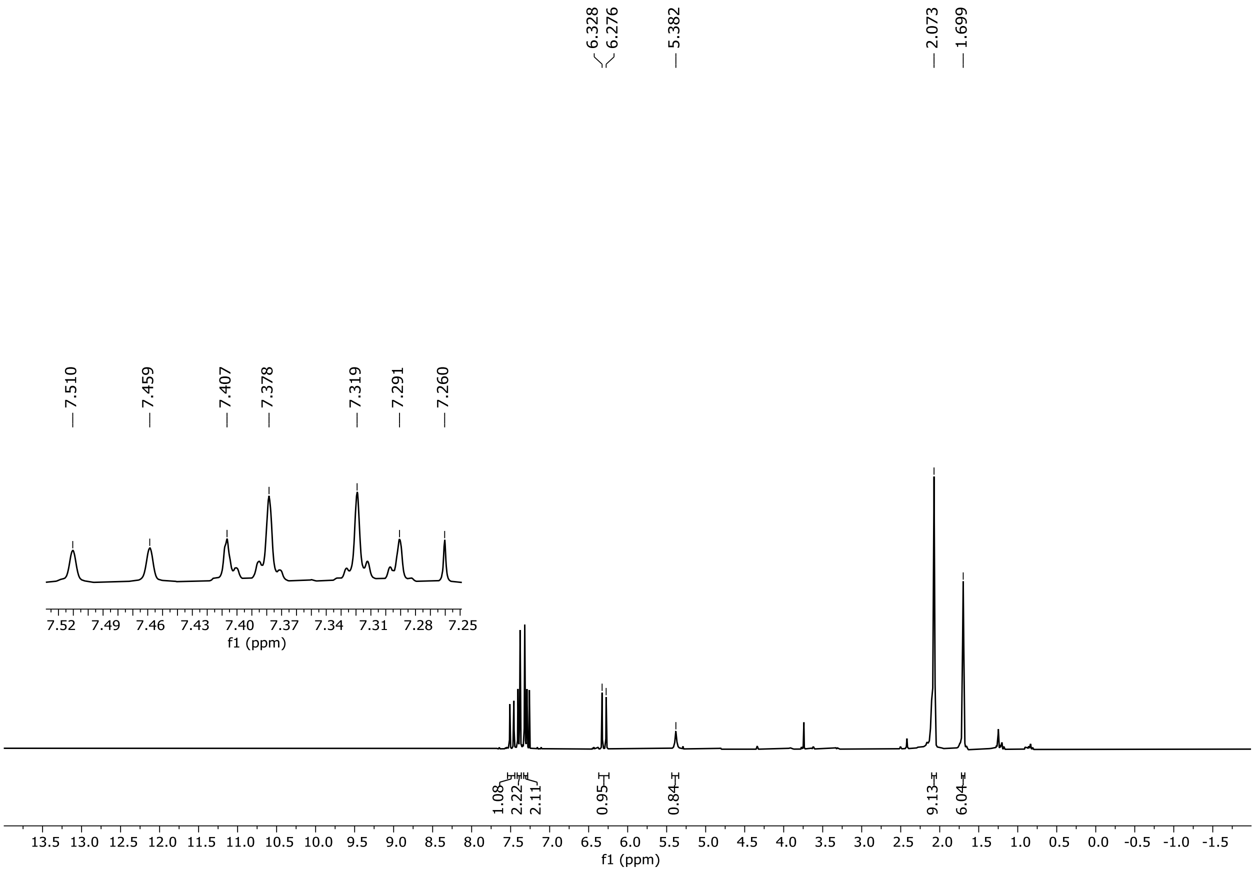

Figure S18. ^{1}H NMR spectrum (300 MHz, $CDCl_3$) of (*E*)-*N*-(adamantan-1-yl)-3-(4-chlorophenyl) acrylamide (**15**).

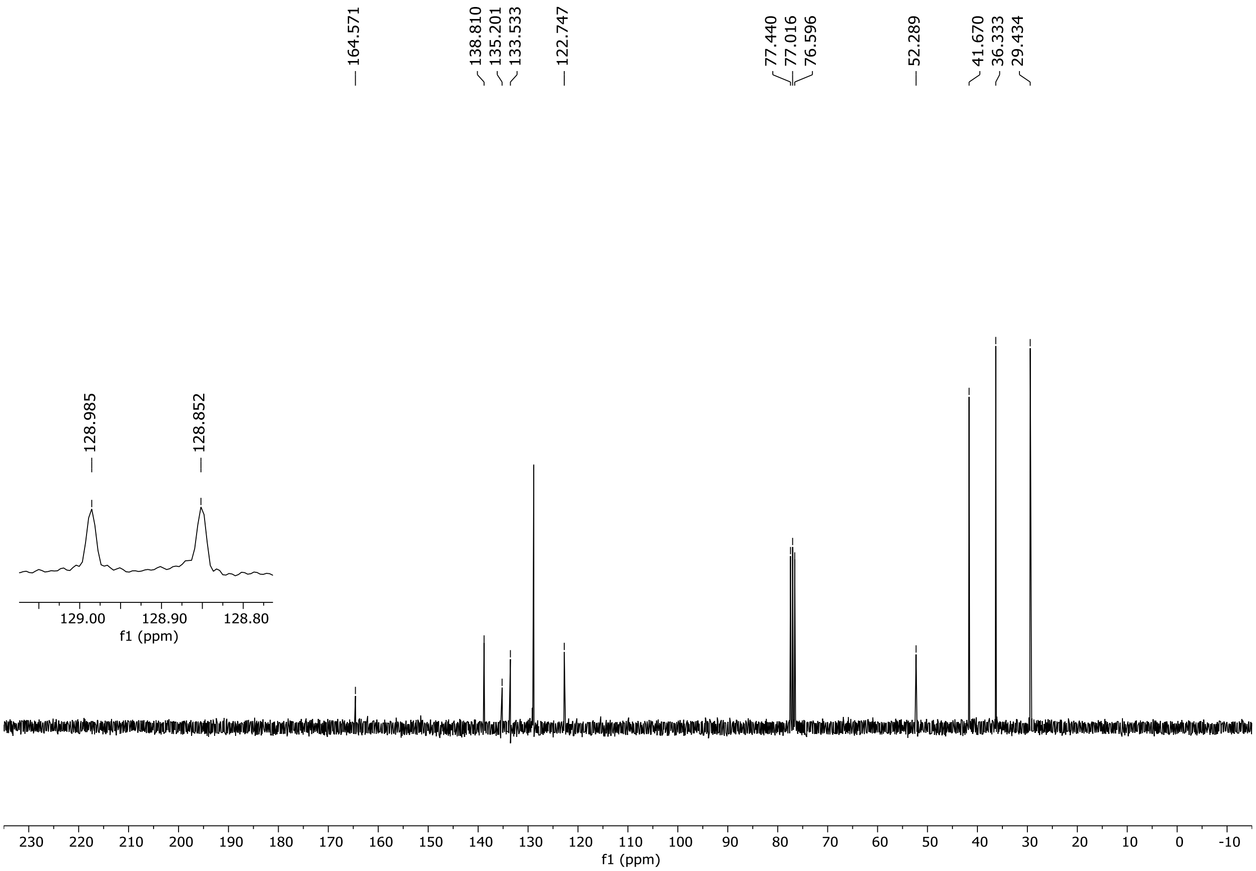

Figure S19. ^{13}C NMR spectrum (75 MHz, $CDCl_3$) of (*E*)-*N*-(adamantan-1-yl)-3-(4-chlorophenyl) acrylamide (**15**).

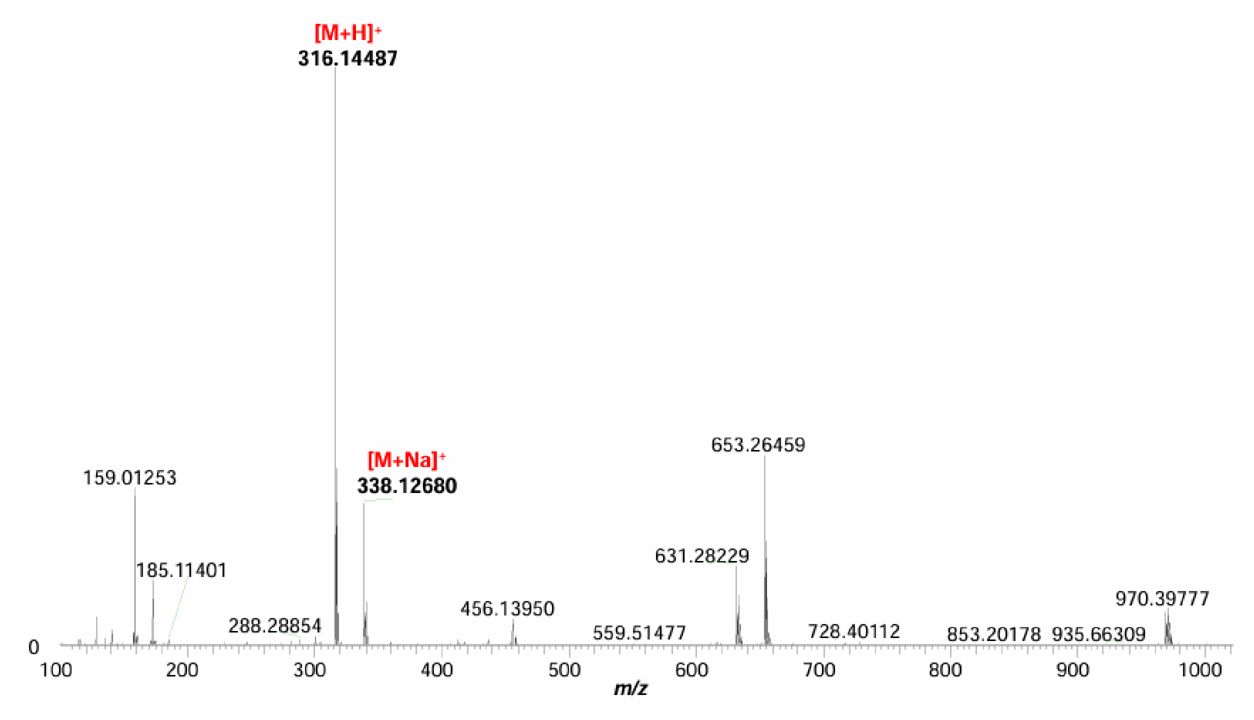

Figure S20. HRMS (ESI) spectrum of (*E*)-*N*-(adamantan-1-yl)-3-(4-chlorophenyl) acrylamide (**15**).

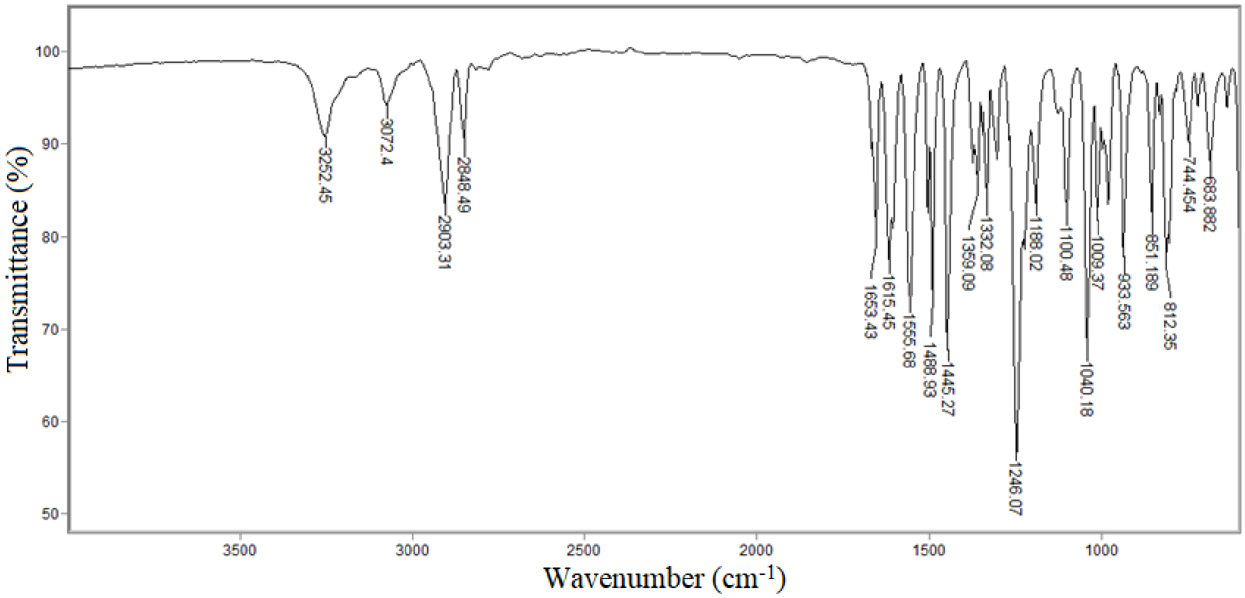

Figure S21. FTIR spectrum (ATR) of (*E*)-*N*-(adamantan-1-yl)-3-(benzo[*d*][1,3] dioxol-5-yl) acrylamide (**16**).

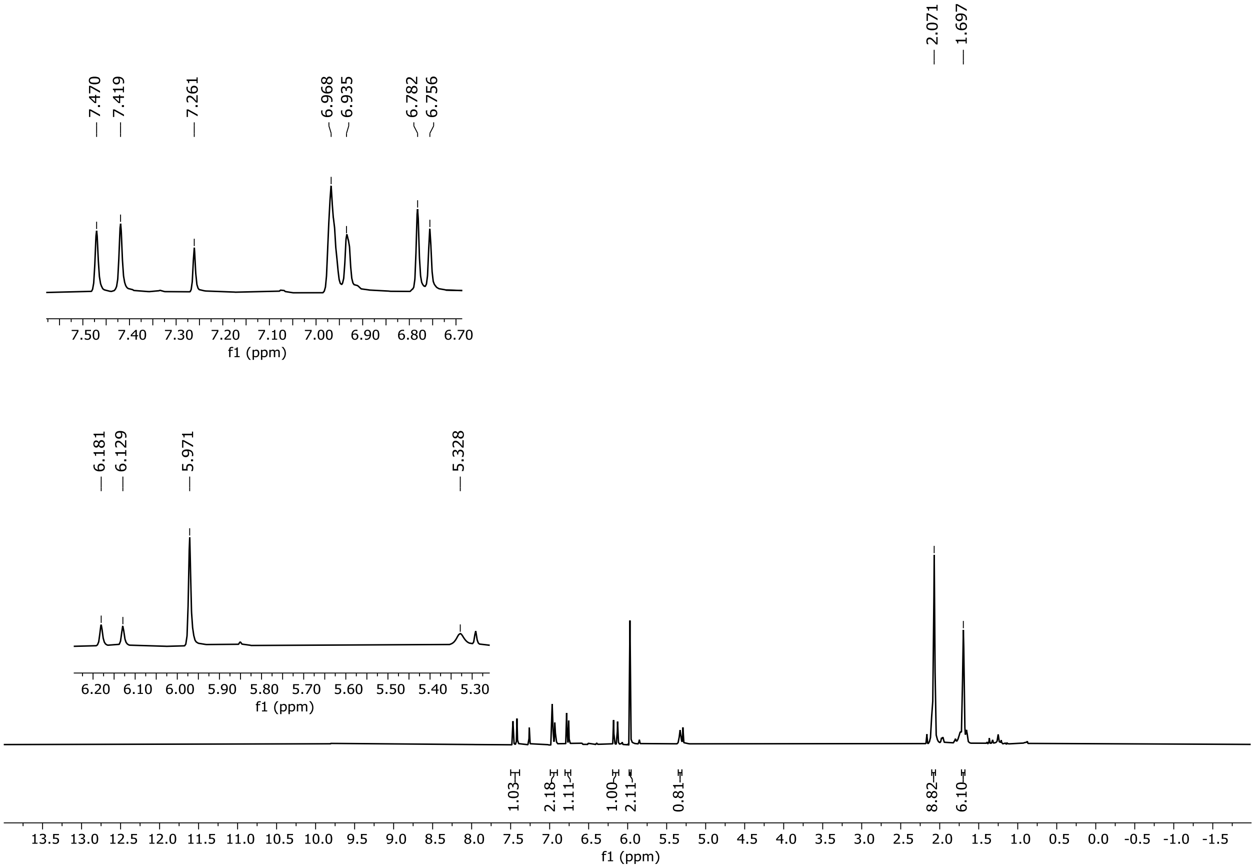

Figure S22. ^{1}H NMR spectrum (300 MHz, $CDCl_3$) of (*E*)-*N*-(adamantan-1-yl)-3-(benzo[*d*][1,3] dioxol-5-yl) acrylamide (**16**).

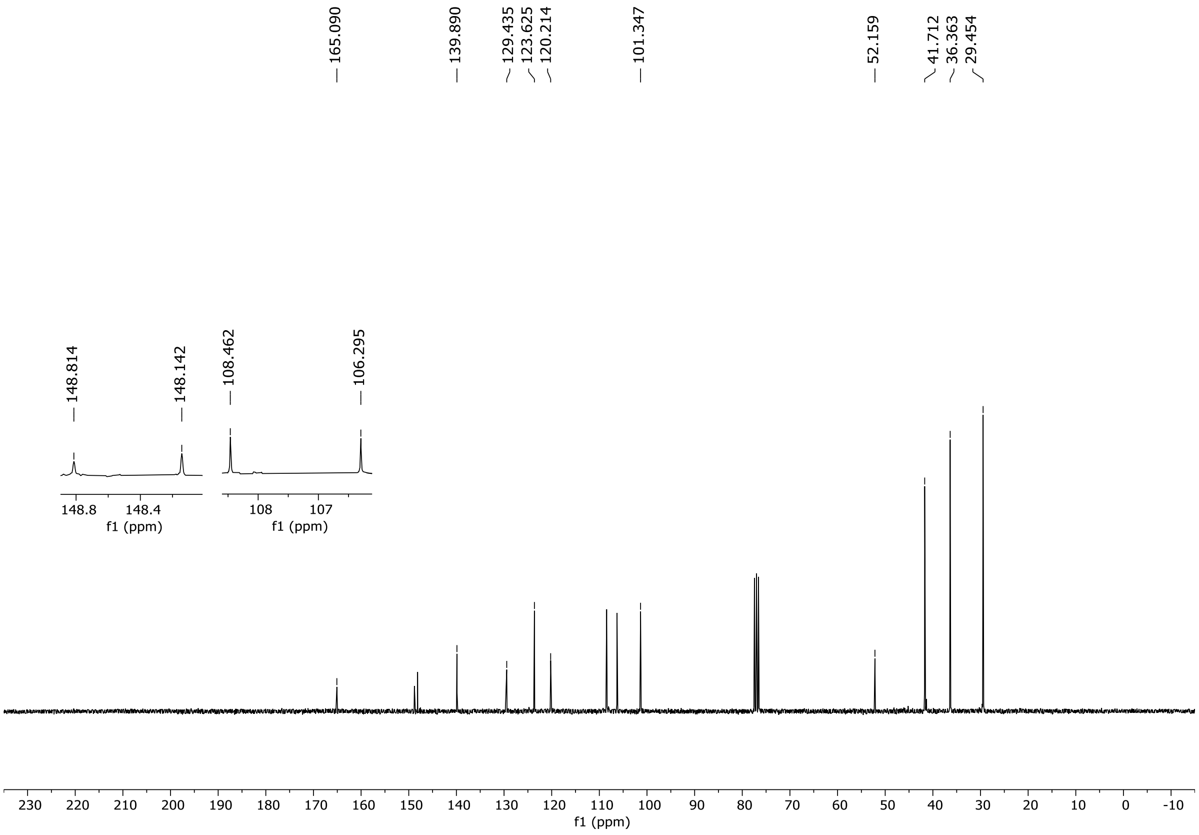

Figure S23. ^{13}C NMR spectrum (75 MHz, $CDCl_3$) of (*E*)-*N*-(adamantan-1-yl)-3-(benzo[*d*][1,3] dioxol-5-yl) acrylamide (**16**).

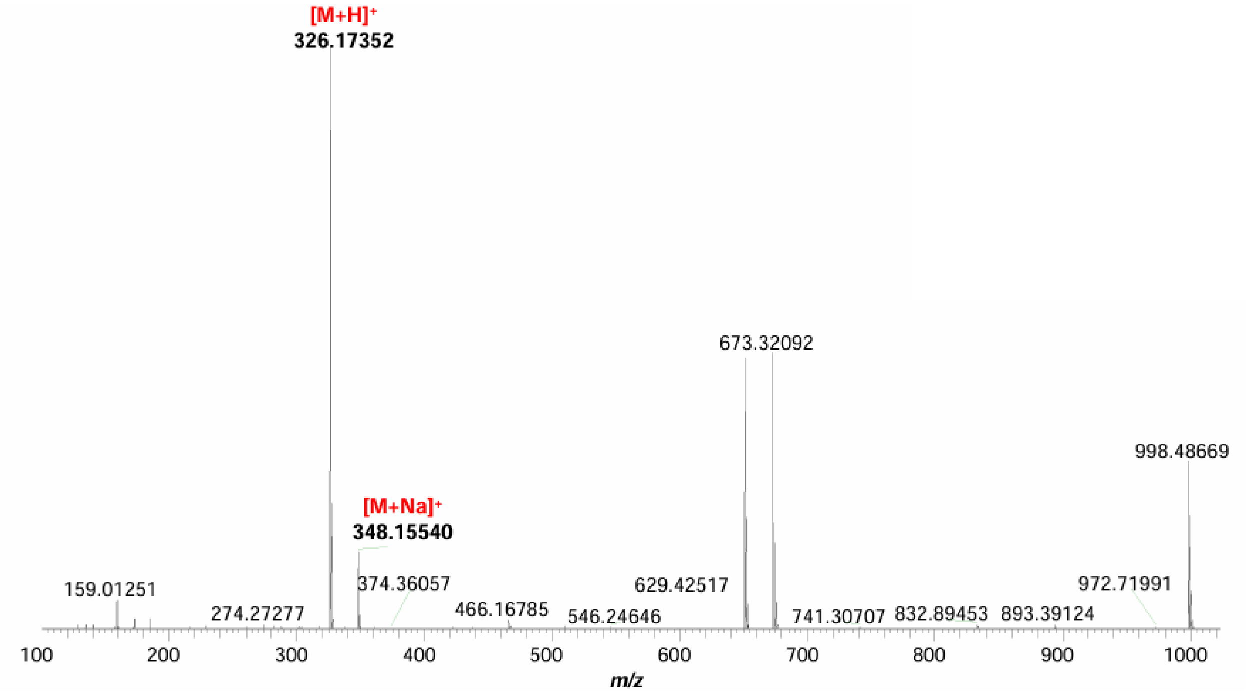

Figure S24. HRMS (ESI) spectrum of of (*E*)-*N*-(adamantan-1-yl)-3-(benzo[*d*][1,3] dioxol-5-yl) acrylamide (**16**).

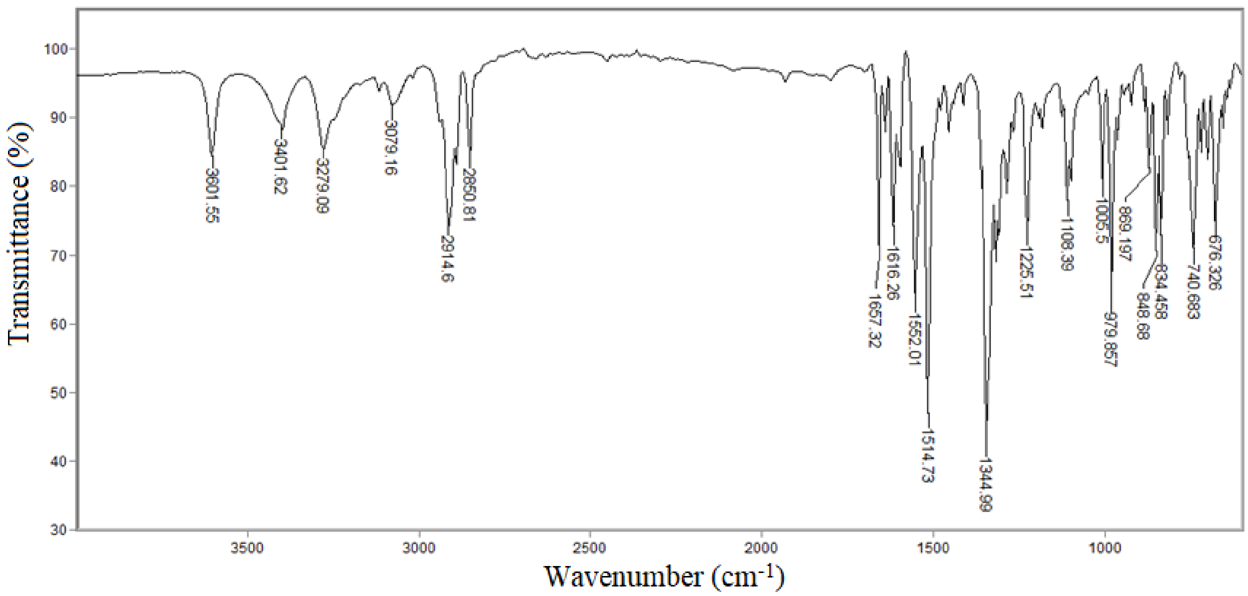

Figure S25. FTIR spectrum (ATR) of (*E*)-*N*-(adamantan-1-yl)-3-(4-nitrophenyl) acrylamide (**17**).

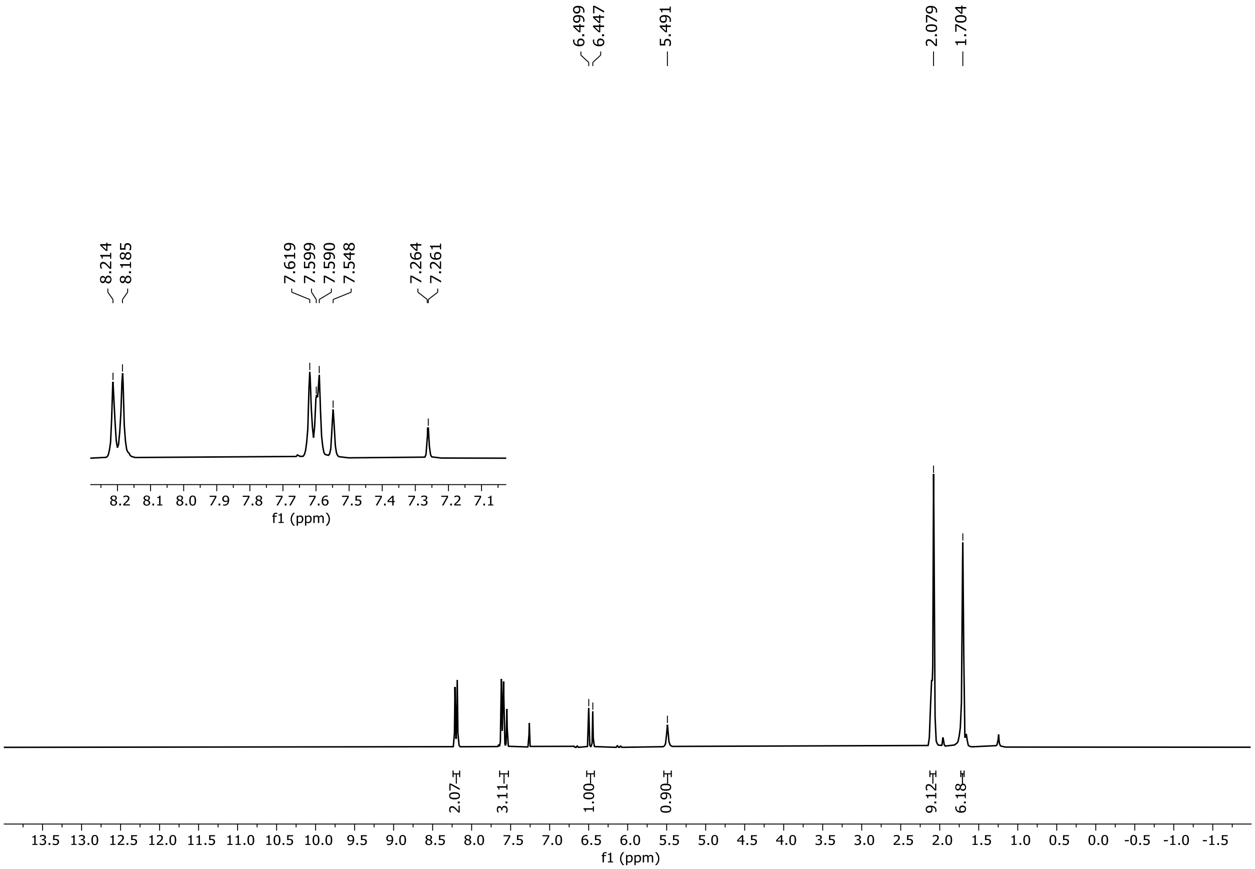

Figure S26. ^{1}H NMR spectrum (300 MHz, $CDCl_3$) of of (*E*)-*N*-(adamantan-1-yl)-3-(4-nitrophenyl) acrylamide (**17**).

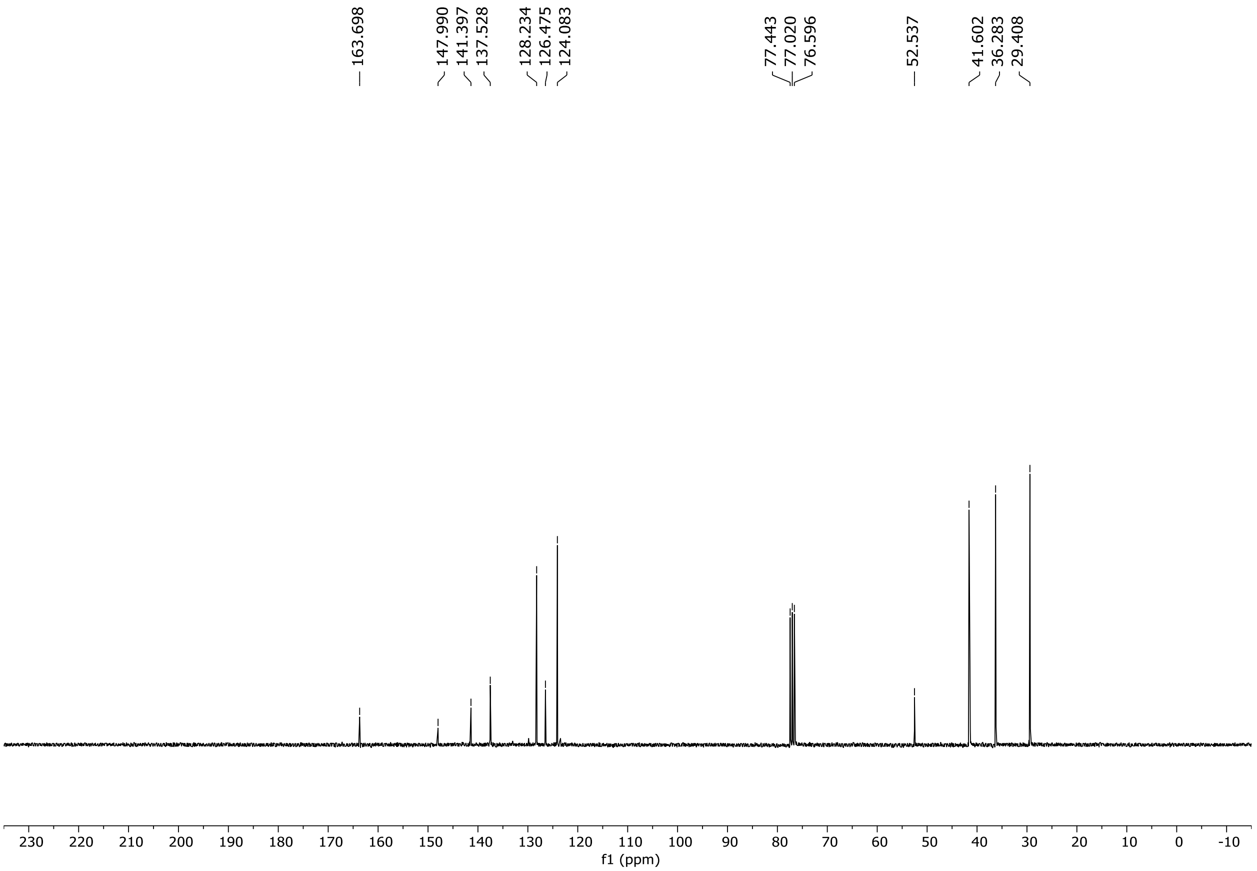

Figure S27. ^{13}C NMR spectrum (75 MHz, $CDCl_3$) of (*E*)-*N*-(adamantan-1-yl)-3-(4-nitrophenyl) acrylamide (**17**).

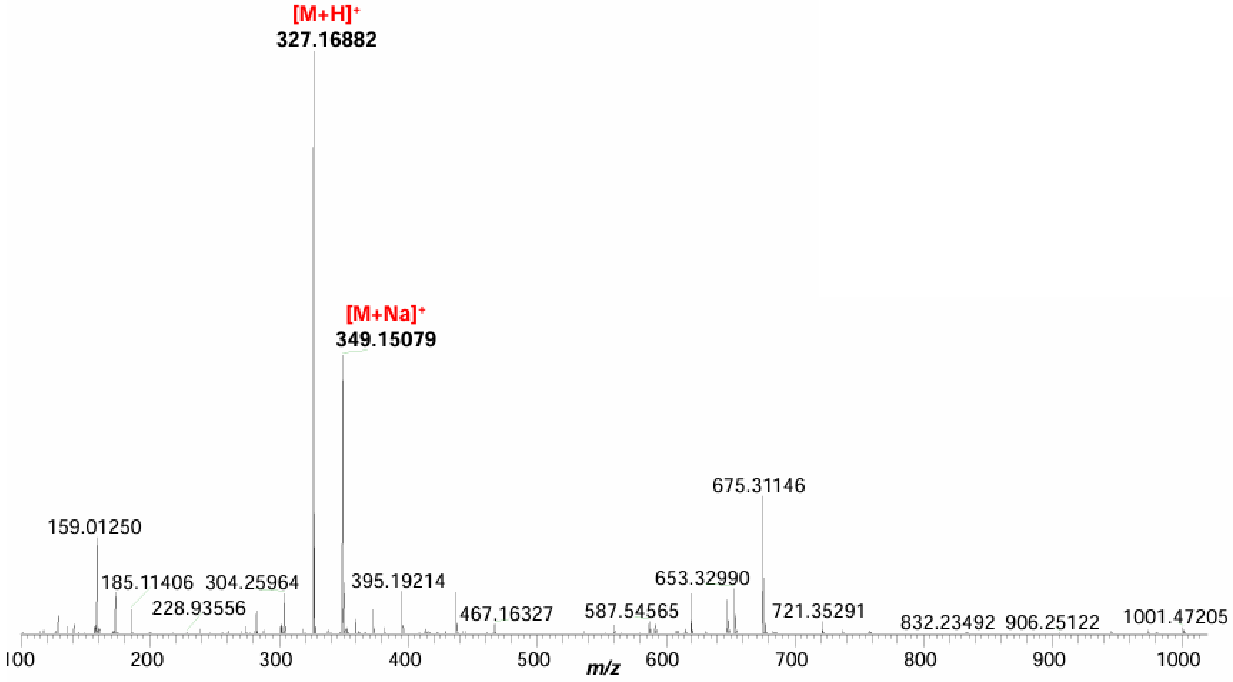

Figure S28. HRMS (ESI) spectrum of (*E*)-*N*-(adamantan-1-yl)-3-(4-nitrophenyl) acrylamide (**17**).

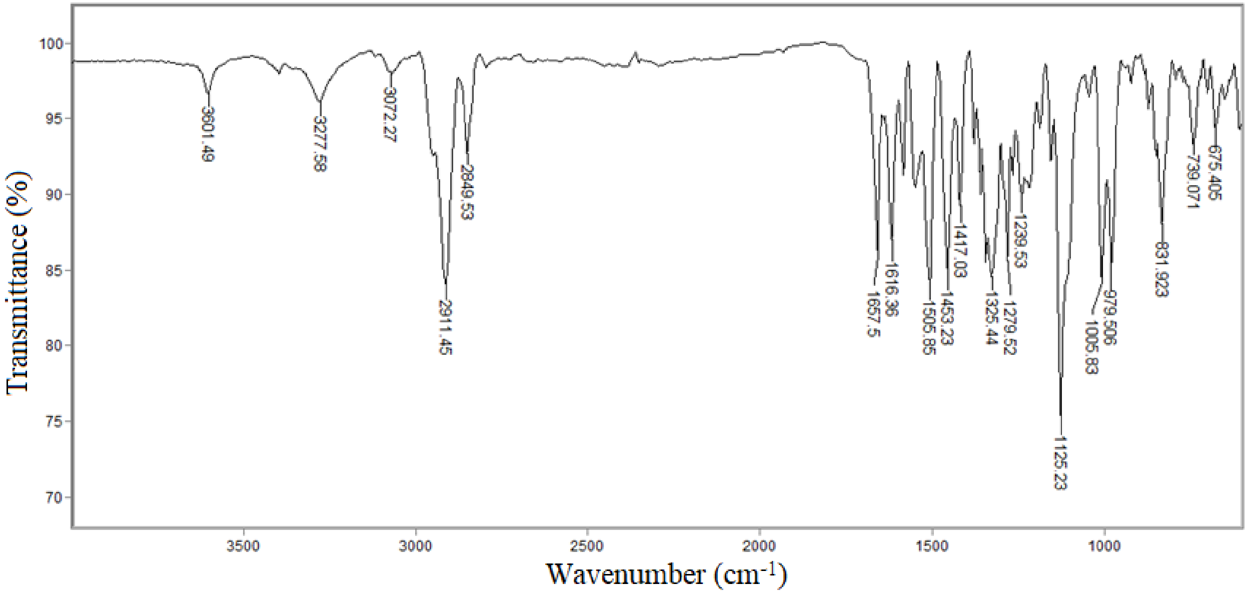

Figure S29. FTIR spectrum (ATR) of (*E*)-*N*-(adamantan-1-yl)-3-(3,4,5-trimethoxyphenyl) acrylamide (**18**).

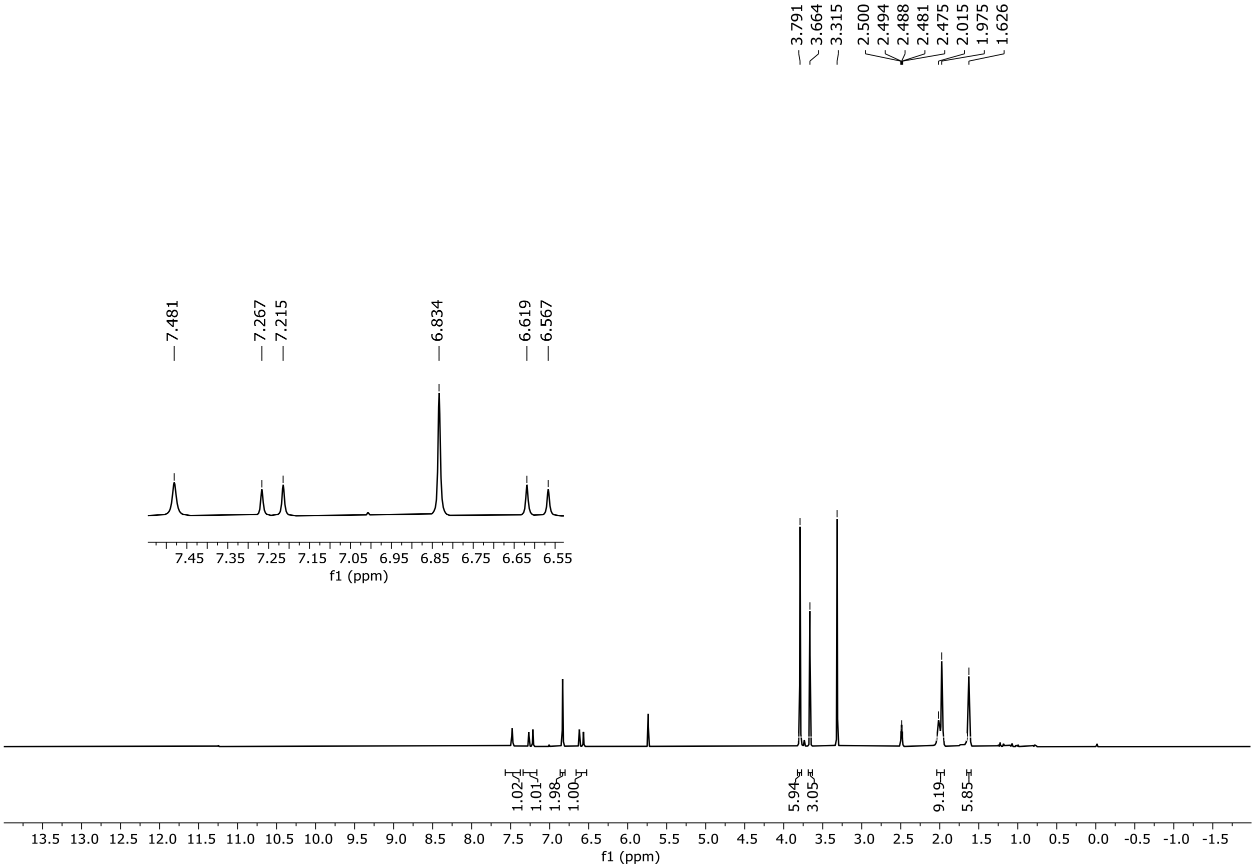

Figure S30. ^{1}H NMR spectrum (300 MHz, DMSO-d_6) of (*E*)-*N*-(adamantan-1-yl)-3-(3,4,5-trimethoxyphenyl) acrylamide (**18**).

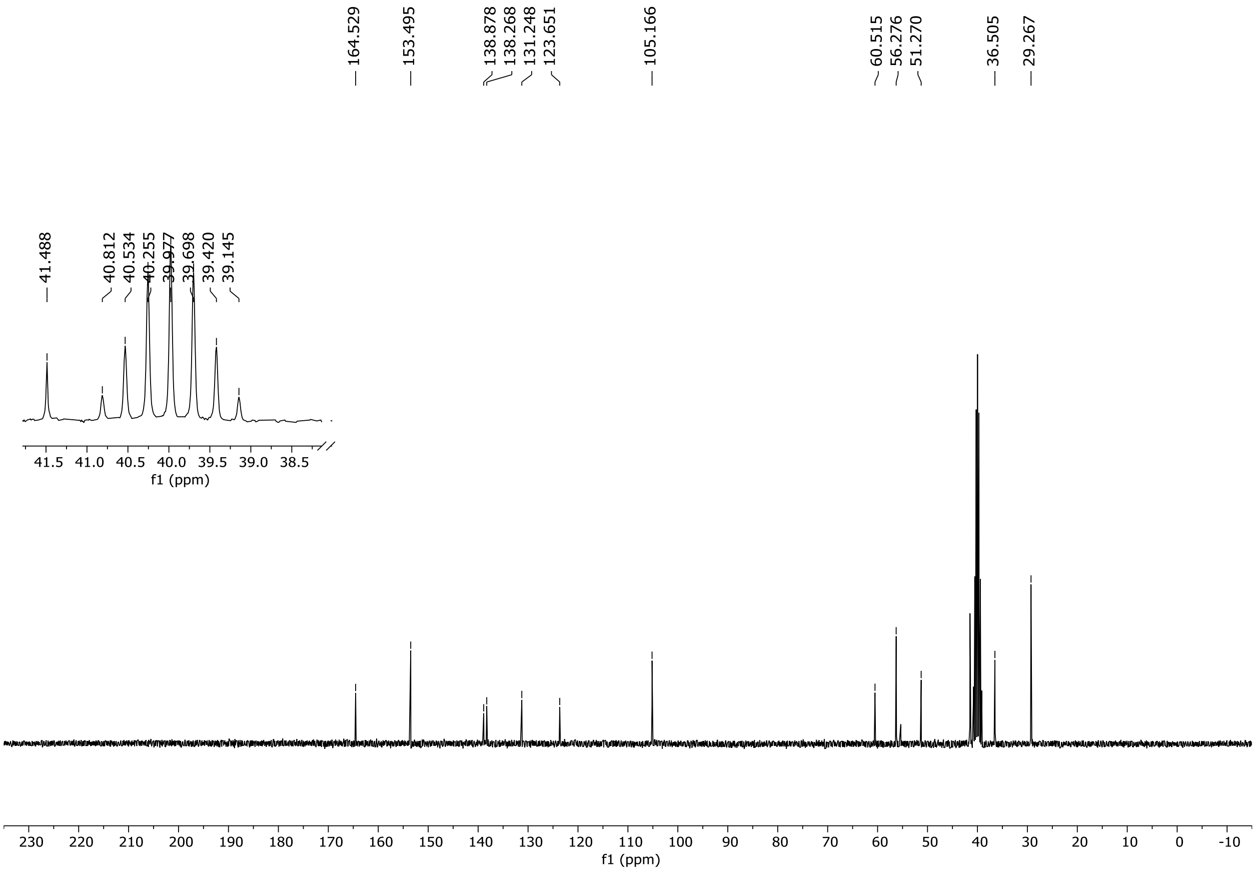

Figure S31. ^{13}C NMR spectrum (75 MHz, DMSO-d_6) of (*E*)-*N*-(adamantan-1-yl)-3-(3,4,5-trimethoxyphenyl) acrylamide (**18**).

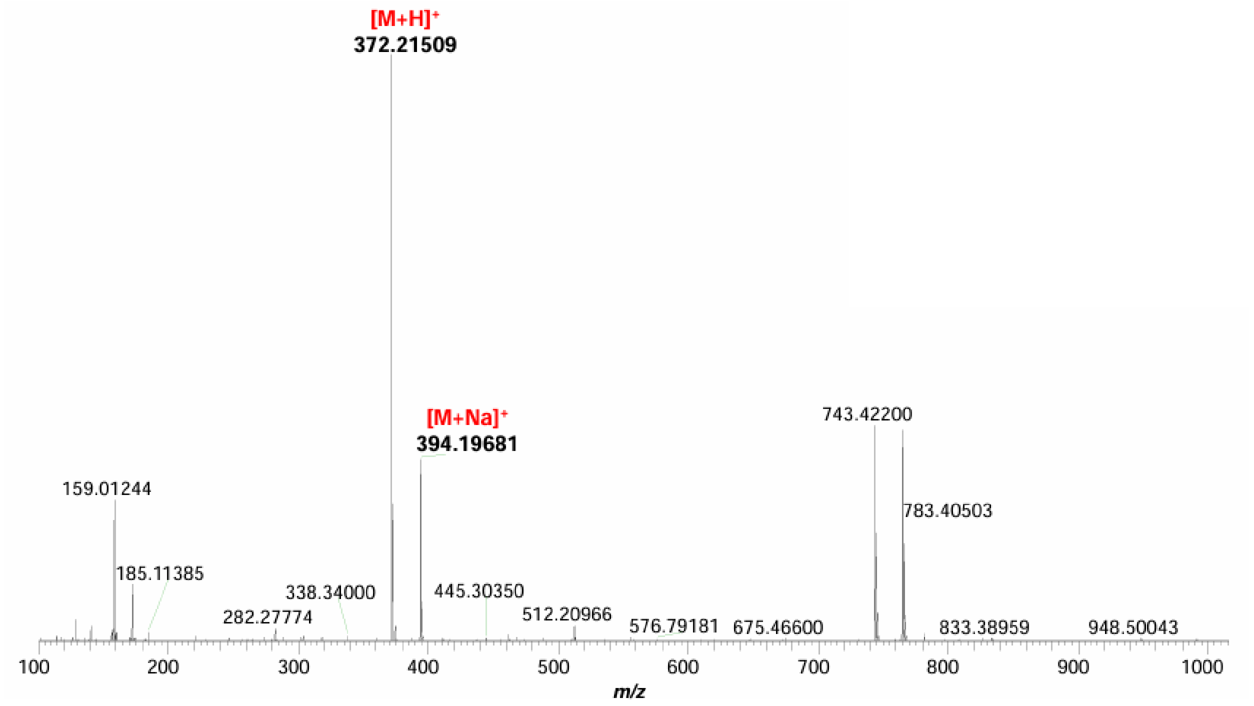

Figure S32. HRMS (ESI) spectrum of (*E*)-*N*-(adamantan-1-yl)-3-(3,4,5-trimethoxyphenyl) acrylamide (**18**).

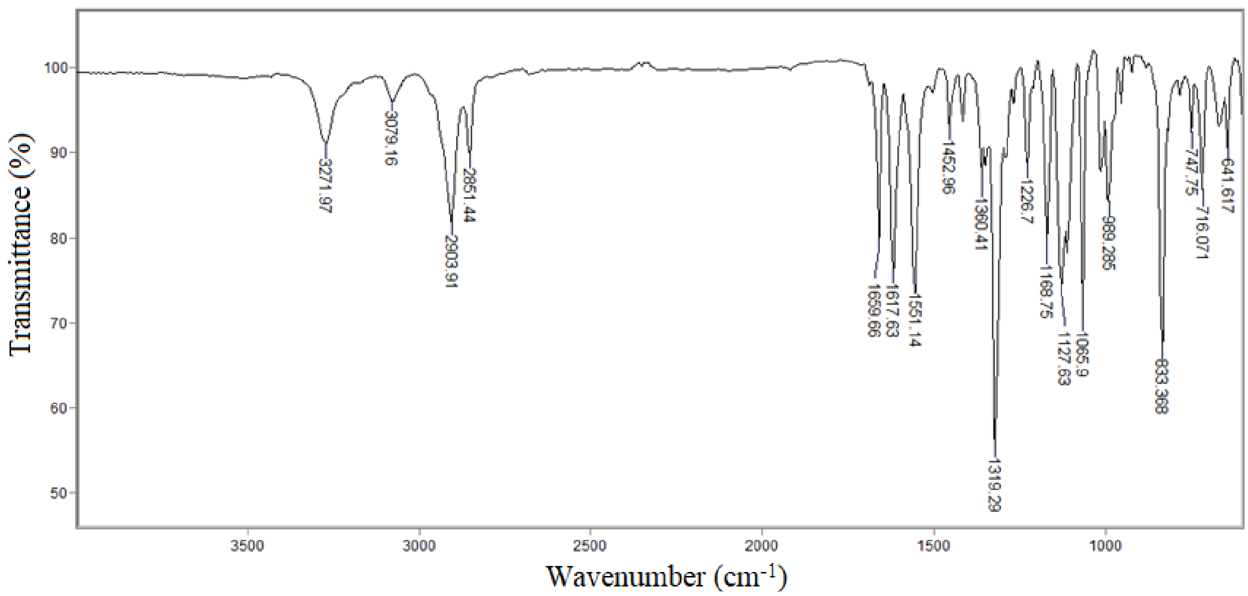

Figure S33. FTIR spectrum (ATR) of(*E*)-*N*-(adamantan-1-yl)-3-(4-(trifluoromethyl) phenyl) acrylamide (**19**).

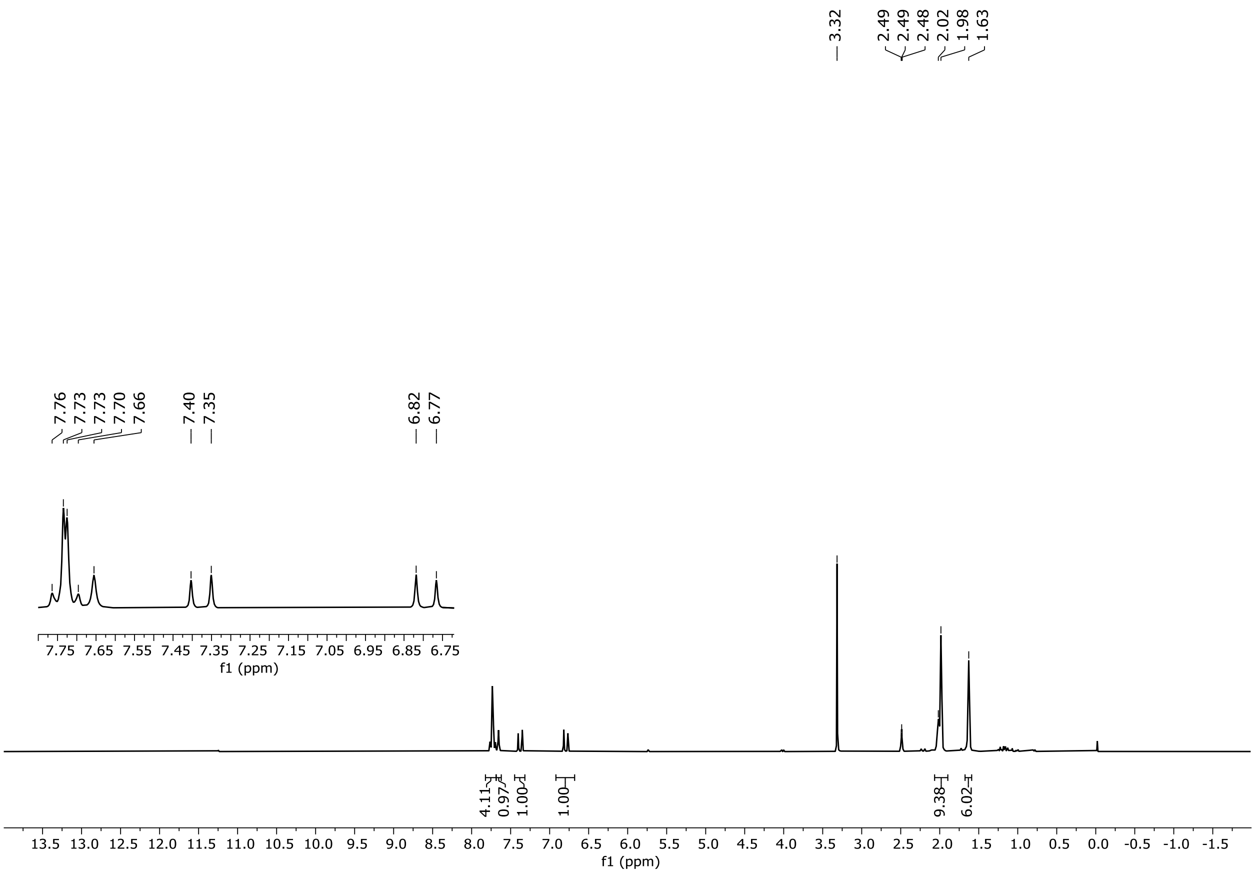

Figure S34. ^{1}H NMR spectrum (300 MHz, DMSO-d_6) of (*E*)-*N*-(adamantan-1-yl)-3-(4-(trifluoromethyl) phenyl) acrylamide (**19**).

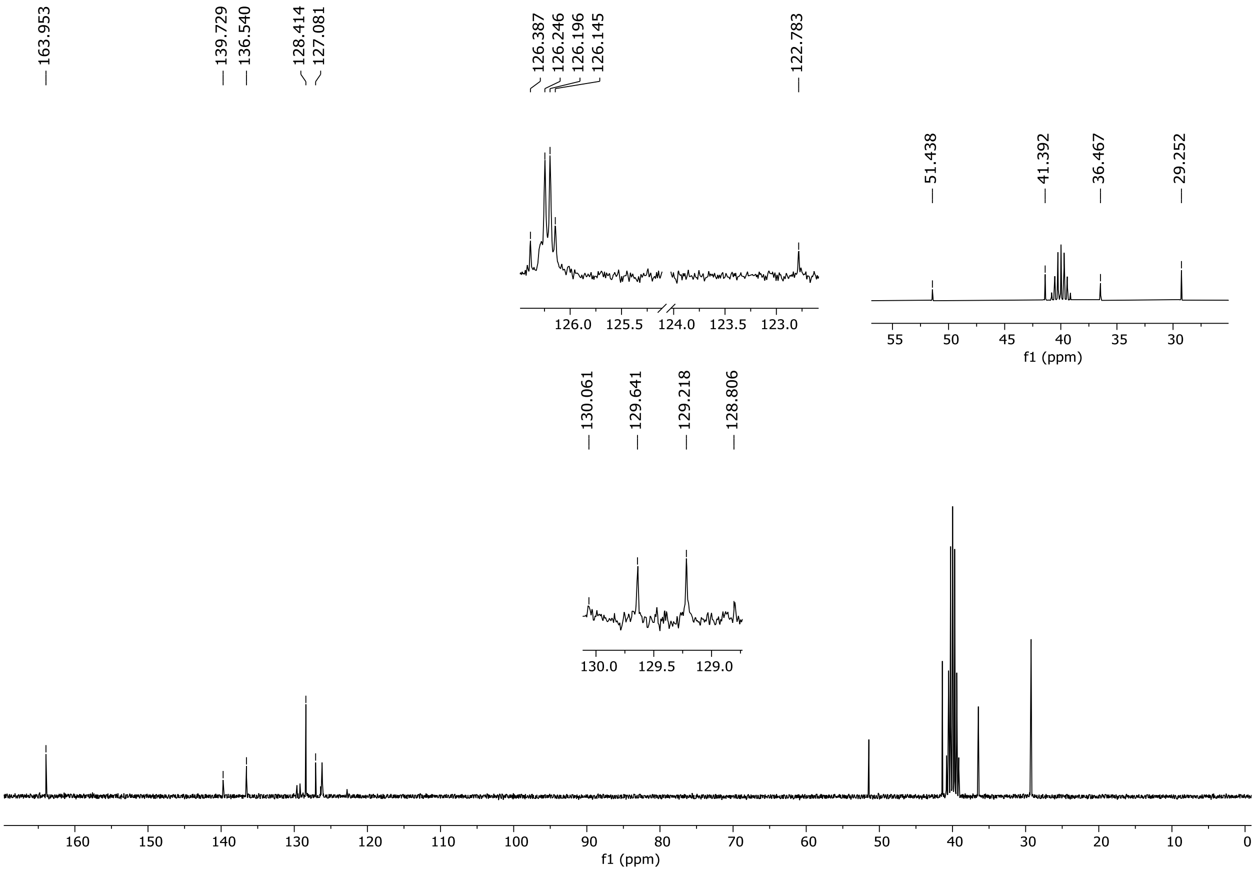

Figure S35. ^{13}C NMR spectrum (75 MHz, DMSO-d_6) of (*E*)-*N*-(adamantan-1-yl)-3-(4-(trifluoromethyl) phenyl) acrylamide (**19**).

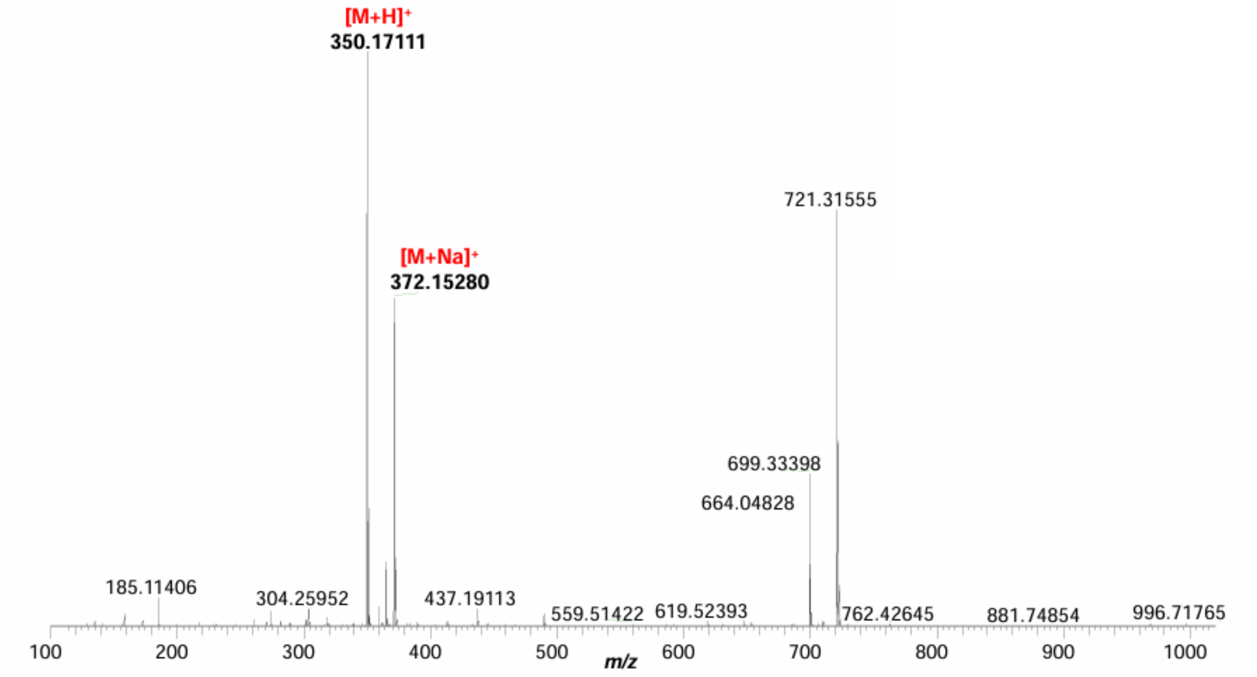

Figure S36. HRMS (ESI) spectrum of (*E*)-*N*-(adamantan-1-yl)-3-(4-(trifluoromethyl) phenyl) acrylamide (**19**).

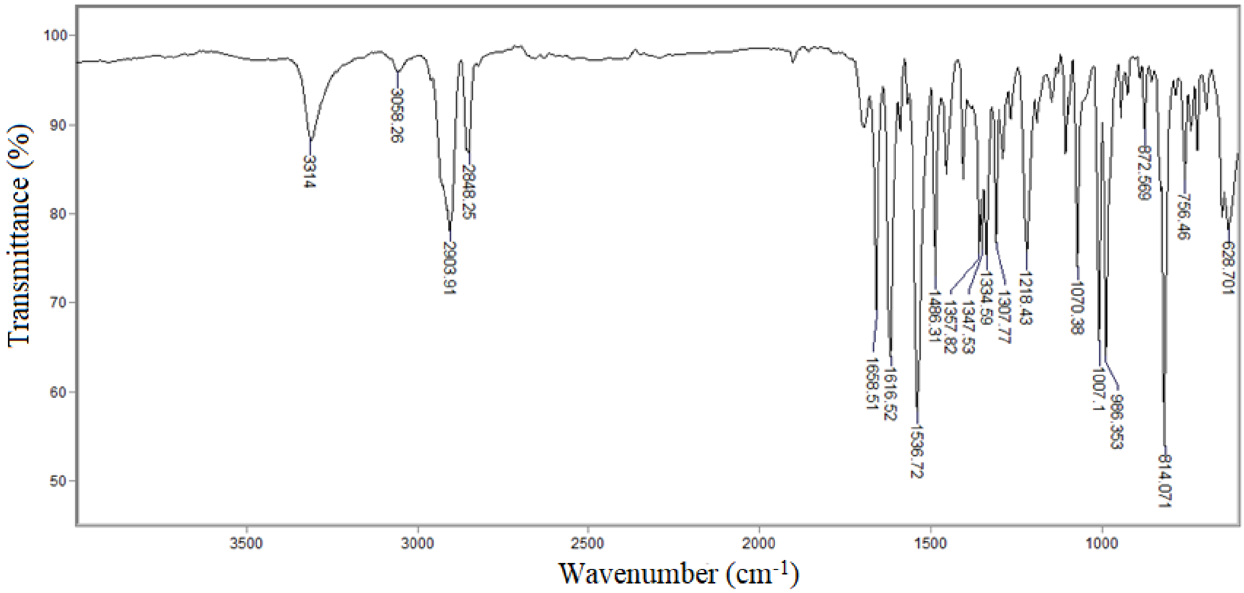

Figure S37. FTIR spectrum (ATR) of (*E*)-*N*-(adamantan-1-yl)-3-(4-bromophenyl) acrylamide (**20**).

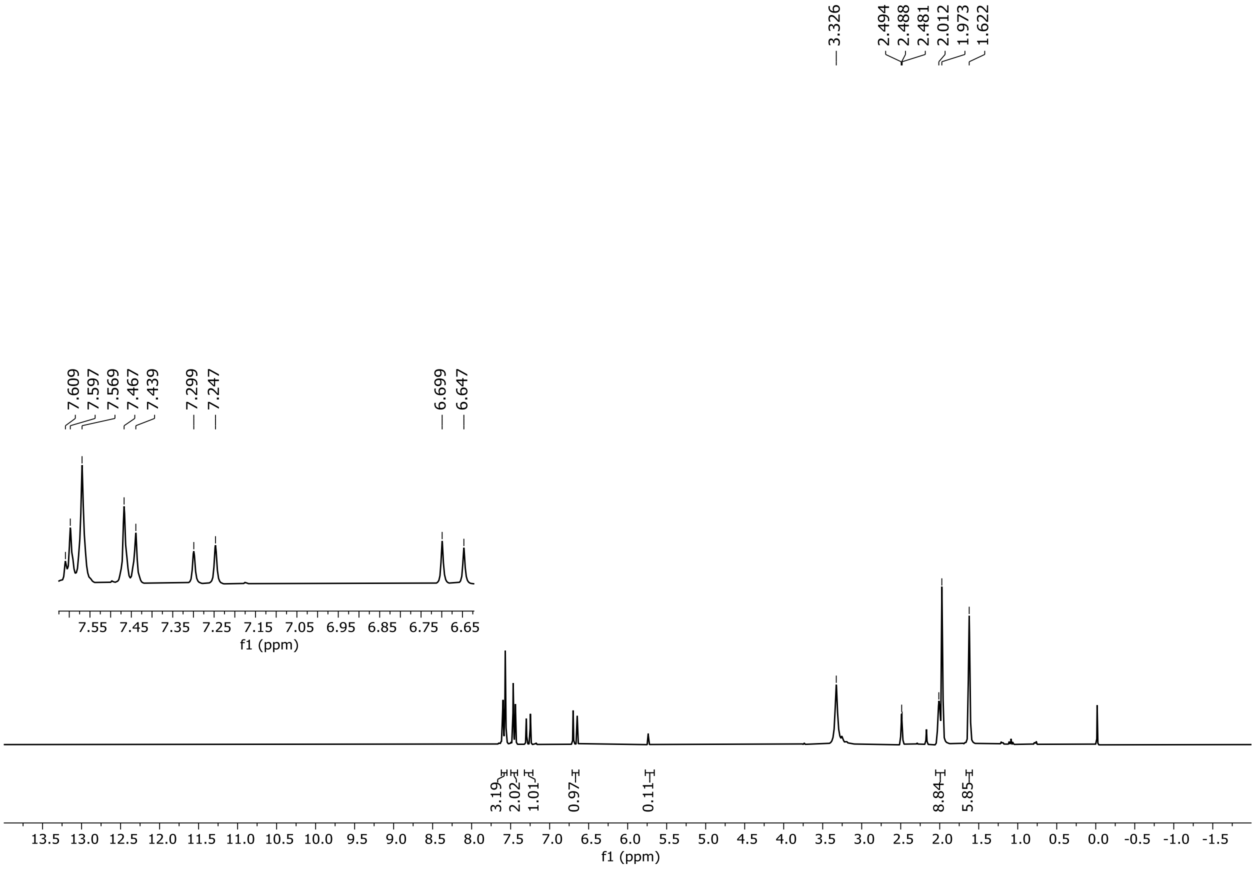

Figure S38. ^{1}H NMR spectrum (300 MHz, DMSO-d_6) of (*E*)-*N*-(adamantan-1-yl)-3-(4-bromophenyl) acrylamide (**20**).

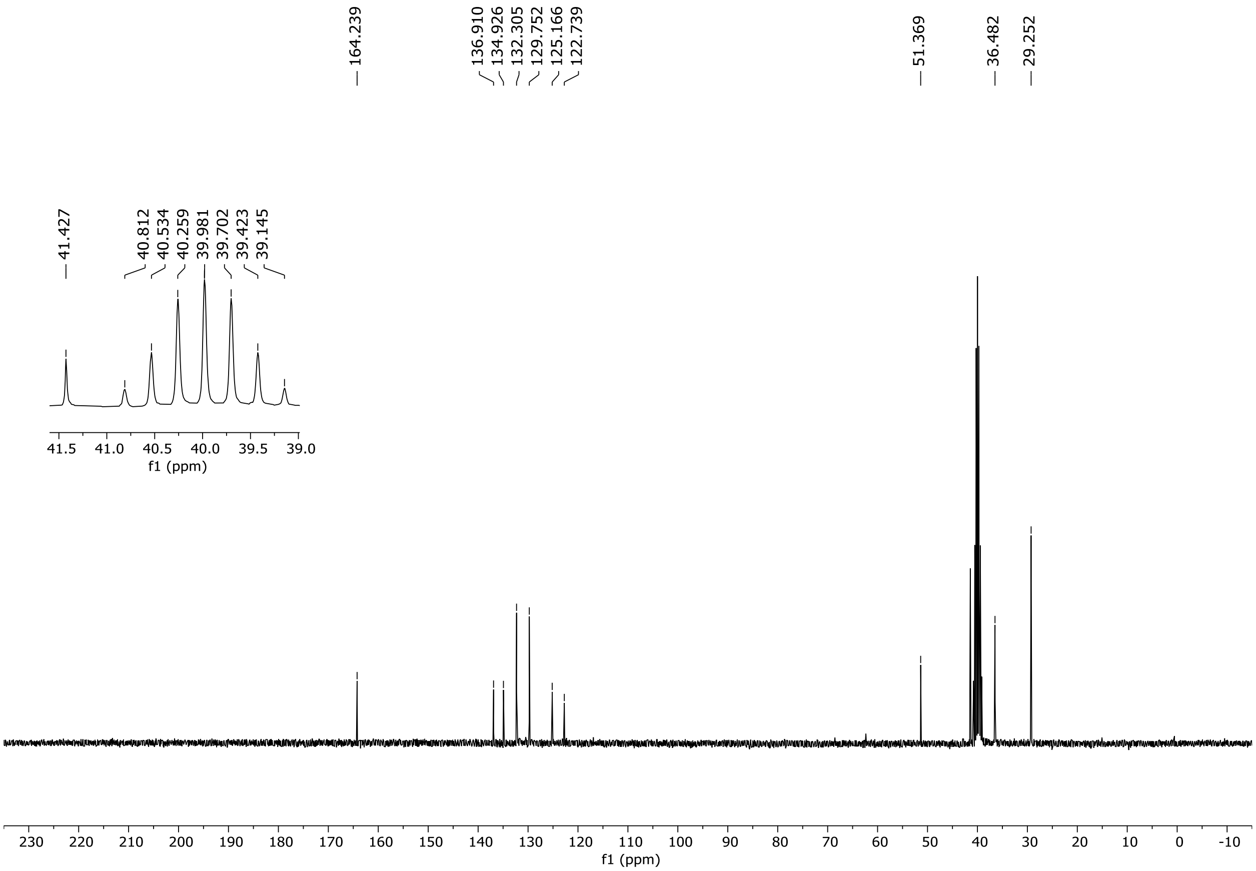

Figure S39. ^{13}C NMR spectrum (75 MHz, DMSO-d_6) of (*E*)-*N*-(adamantan-1-yl)-3-(4-bromophenyl) acrylamide (**20**).

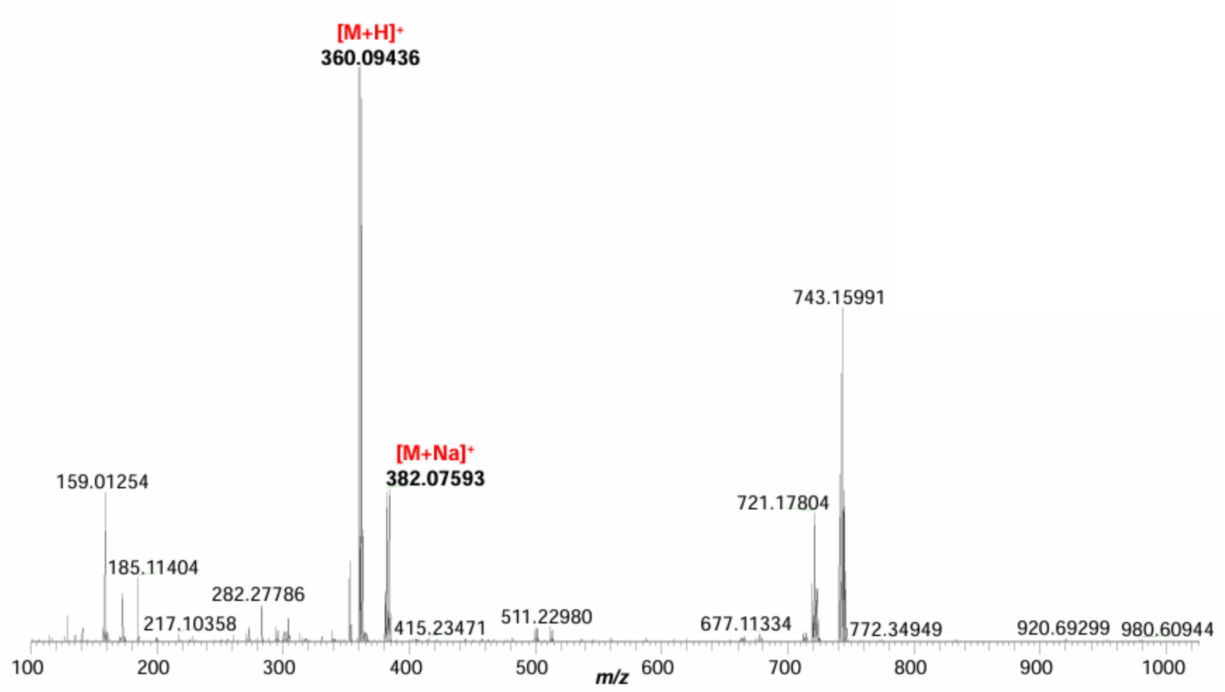

Figure S40. HRMS (ESI) spectrum of (*E*)-*N*-(adamantan-1-yl)-3-(4-bromophenyl) acrylamide (**20**).
